# A new candidate epitope-based vaccine against PspA PhtD of *Streptococcus pneumoniae*: a computational experimental approach

**DOI:** 10.3389/fcimb.2023.1271143

**Published:** 2023-11-15

**Authors:** Mona Shafaghi, Zohreh Bahadori, Seyed Mahmoud Barzi, Elnaz Afshari, Hamid Madanchi, Seyed Fazlollah Mousavi, Ali Akbar Shabani

**Affiliations:** ^1^ Department of Medical Biotechnology, faculty of Medicine, Semnan University of Medical Sciences, Semnan, Iran; ^2^ Department of Bacteriology, Pasteur Institute of Iran, Tehran, Iran; ^3^ Department of Biology, Science and Research Branch, Islamic Azad University, Tehran, Iran; ^4^ Drug Design and Bioinformatics Unit, Department of Medical Biotechnology, Biotechnology Research Center, Pasteur Institute of Iran, Tehran, Iran

**Keywords:** *Streptococcus pneumoniae*, pneumococcal surface protein A (PspA), pneumococcal histidine triad protein D (PhtD), pneumococcal epitope-based vaccine, immunodominant B and T cell epitopes, immunoinformatics, actual animal experiments, fusion protein

## Abstract

**Introduction:**

Pneumococcus is an important respiratory pathogen that is associated with high rates of death in newborn children and the elderly. Given the disadvantages of current polysaccharide-based vaccines, the most promising alternative for developing improved vaccines may be to use protein antigens with different roles in pneumococcus virulence. PspA and PhtD, highly immunogenic surface proteins expressed by almost all pneumococcal strains, are capable of eliciting protective immunity against lethal infections.

**Methods:**

In this study using immunoinformatics approaches, we constructed one fusion construct (called PAD) by fusing the immunodominant regions of PspA from families 1 & 2 (PA) to the immunodominant regions of PhtD (PD). The objective of this project was to test the immunogenicity of the fusion protein PAD and to compare its protective activity against *S. pneumoniae* infection with PA or PD alone and a combination of PA and PD. The prediction of physicochemical properties, antigenicity, allergenicity, toxicity, and 3D-structure of the constructs, as well as molecular docking with HLA receptor and immune simulation were performed using computational tools. Finally, mice were immunized and the serum levels of antibodies/cytokines and functionality of antibodies *in vitro* were evaluated after immunization. The mice survival rates and decrease of bacterial loads in the blood/spleen were examined following the challenge.

**Results:**

The computational analyses indicated the proposed constructs could be antigenic, non-allergenic, non-toxic, soluble and able to elicit robust immune responses. The results of actual animal experiments revealed the candidate vaccines could induce the mice to produce high levels of antibodies and cytokines. The complement-mediated bactericidal activity of antibodies was confirmed and the antibodies provided favorable survival in immunized mice after bacterial challenge. In general, the experimental results verified the immunoinformatics studies.

**Conclusion:**

For the first time this report presents novel peptide-based vaccine candidates consisting of immunodominant regions of PspA and PhtD antigens. The obtained findings confirmed that the fusion formulation could be relatively more efficient than the individual and combination formulations. The results propose that the fusion protein alone could be used as a serotype-independent pneumococcal vaccine or as an effective partner protein for a conjugate polysaccharide vaccine.

## 1 Introduction

*Streptococcus pneumoniae* (pneumococcus) has remained one of the main causes of lethal diseases such as pneumonia and meningitis, and of secondary infections following respiratory viral diseases including influenza and COVID-19 (Bahadori et al., 2022). The high morbidity and mortality rates in more than 1 million cases, particularly in children under 2 years old and the elderly above 65 years old, are the major driving force for the development of pneumococcal vaccines (Shafaghi et al., 2023). There are currently two types of commercial pneumococcal vaccines comprising of unconjugated or protein-conjugated polysaccharides, both of which have advantages and disadvantages (Goulart et al., 2017). The 23-valent polysaccharide vaccine has the potential to cover many serotypes. However, this vaccine is weakly immunogenic in high-risk groups of young children due to the poor immunogenicity of the T cell-independent antigens (Nguyen et al., 2011). The protein–polysaccharide conjugate vaccines have been in use for many years and could lead to more effective immune responses even in the high-risk populations, but there are many limitations including high cost, limited serotype coverage, and serotype replacement following vaccination with these vaccines (Lu et al., 2015). The antigenic proteins of pneumococcus that are highly conserved and are expressed on the bacterial surface at different stages of pathogenesis are expected to be particularly useful as vaccine antigens. Two of the well-characterized protein candidates are pneumococcal surface protein A (PspA) and pneumococcal histidine triad protein D (PhtD) (Plumptre et al., 2013a; Lu et al., 2015).

PspA is one of the key virulence factors in all clinical isolates of pneumococcus, which plays important roles including preventing the deposition of complement and the bactericidal activity of lactoferrin (Shaper et al., 2004; Mukerji et al., 2012). The PspA protein possesses 3 main domains: alpha helix domain consisting of the regions A, Aˊ, and B (α-HD, residues 1-288), proline-rich domain (region C, residues 289-370), and choline binding domain (residues 371-571) (Hollingshead et al., 2000). The N-terminal alpha helix domain and proline-rich domain are exposed on the surface and are capable of interacting with the host’s immune system (Daniels et al., 2010; Vadesilho et al., 2014). The alpha helical domain is highly immunogenic and variable in sequence among different strains. The protection appears to be mediated by epitopes within 100 residues at the N-terminus (region A) and about 100 residues at the C-terminus (region B) of this domain (McDaniel et al., 1994; Roche et al., 2003; Vadesilho et al., 2014). PspA is divided into 3 families including 6 clades according to the sequence diversity in the B region or clade-defining region (CDR) (Hollingshead et al., 2000). Family one includes clades 1 & 2, family two includes classes 3, 4 & 5, and family three includes the rare clade 6 (Hollingshead et al., 2000). The studies have shown that more than 95% of the isolated pneumococcal strains belong to families 1 & 2, which is why efforts for developing PspA-based vaccines are focused on these 2 families (Converso et al., 2017b; Mukerji et al., 2018). The cross-reactivity level among different PspAs depends on the degree of sequence similarity so that there is a higher degree of cross-reactivity in the same clade (Miyaji et al., 2002). It has found that the immunity elicited by the 2 main PspA families are clade dependent (Miyaji et al., 2002; Darrieux et al., 2008; Goulart et al., 2011), thus it is suggested that the highly antigenic fragments of all clades should be included in the PspA-based vaccine (Akbari et al., 2019). Moreover, the A and C regions of PspA, which possess the more conserved domains, could help to expand the cross protection (Darrieux et al., 2008; Kristian et al., 2016).

PhtD is one of the known polyhistidine triad proteins expressed by all pneumococcal strains and is distinguished by the presence of 5 copies of the His triad motif (Plumptre et al., 2012). It has been shown that this protein mediates bacterial adhesion due to its high affinity with zinc, and also can inhibit complement deposition (Lagousi et al., 2019). The C-terminal segment of protein PhtD (PhtD-C) is exposed on the bacterial surface, making it a more immunogenic region (Malekan et al., 2019; Bahadori et al., 2022). The studies have revealed that vaccination with the truncated derivatives of PhtD-C was more effective in inducing antibodies and protective immune responses in comparison to vaccination with the full length protein (Plumptre et al., 2013b).

Bioinformatics techniques could help scientists in different biological areas (Shafaghi et al., 2019; Nabizadeh et al., 2020; Bahadori et al., 2021), especially for anticipation of potential immunoprotective epitopes to develop vaccine candidates (Tapia et al., 2016; Fereshteh et al., 2022). In fact, the tools of immunoinformatics provide benefits in comparison with traditional vaccinology methods such as lower costs and faster outcomes (Afshari et al., 2023a). In this study, immunoinformatics and computational tools were used to design a novel fusion protein with several peptides from different domains of PspA and PhtD; peptides from PspA (region A, region B for both families 1 & 2, and region C) and PhtD-C. In addition to the fusion construct, two individual constructs corresponding to PspA or PhtD were also constructed. Then, we investigated whether vaccination with the peptide-based fusion construct is able to induce more effective protective responses than vaccination with each of the individual constructs alone or a combination of them.

## 2 Materials and methods

**Figure 1:**
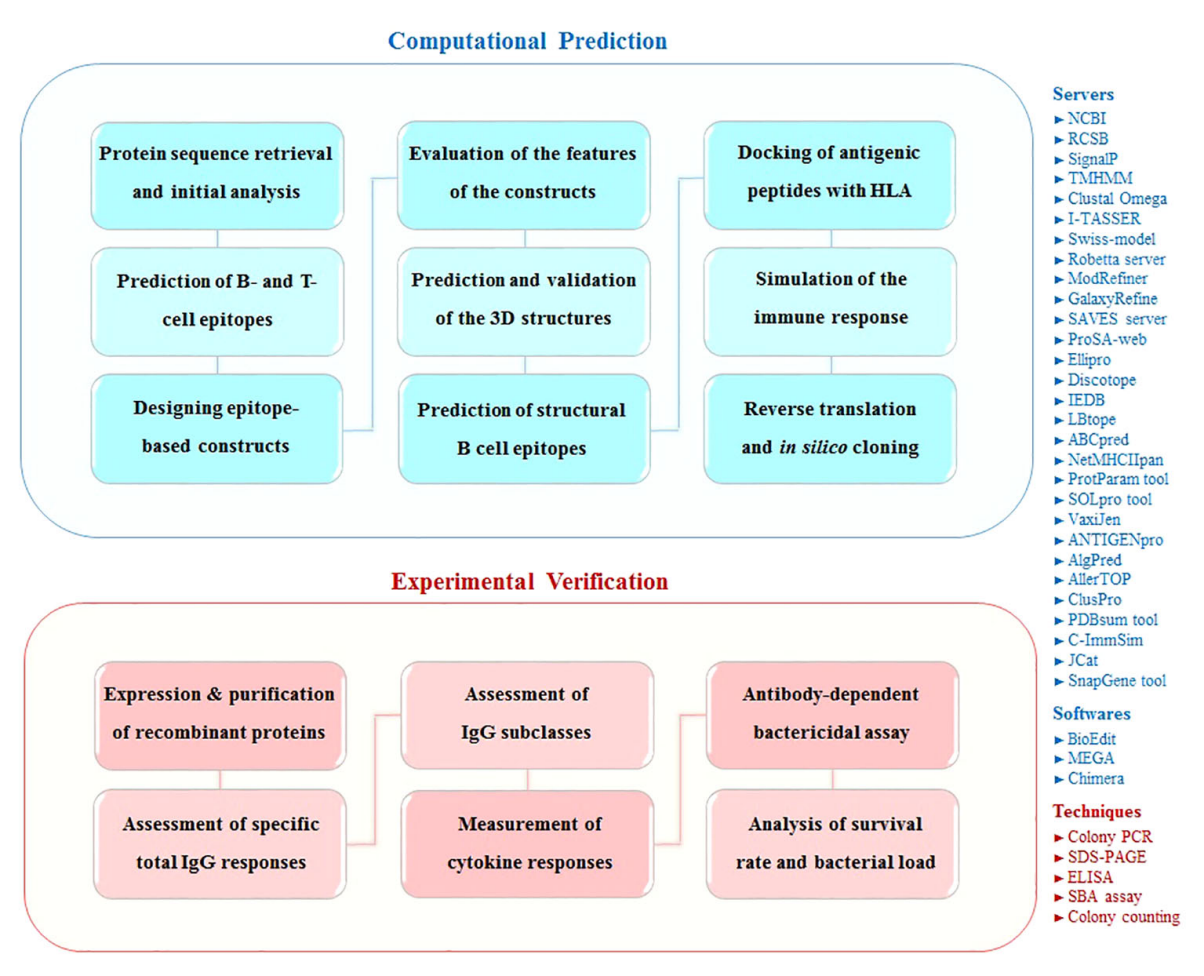
Schematic flowchart of this study for designing and evaluation epitope-based constructs against *S. pneumoniae*. The overall approach of this study is represented in two computational and experimental phases. The used servers/softwares/techniques are mentioned in the right side of the schematic figure.

The present study was performed in two phases: computational prediction and experimental verification. **Figure 1** shows the schematic flowchart of this study.

### 2.1 Computational prediction

#### 2.1.1 Retrieval of amino acid sequences of PspA and PhtD, and preliminary analysis

The amino acid sequences of PspA and PhtD proteins were extracted from the National Center for Biotechnology Information (NCBI) database (www.ncbi.nlm.nih.gov/protein). Due to the diversity of the PspA molecule in different pneumococcal strains, the amino acid sequences of PspA were retrieved from 123 pneumococcal strains which had unique sequences (Mukerji et al., 2018). The retrieved accession numbers of PspA protein sequences are given in **Supplementary Table S1**. The PhtD protein sequence from strain R6 (accession number: AAK99711) was also stored in FASTA format. The possible signal peptide of the proteins was identified using SignalP server (http://www.cbs.dtu.dk/services/SignalP/) (Nielsen, 2017). Moreover, the presence of transmembrane regions in PspA and PhtD-C proteins were investigated using the TransMembrane Hidden Markov Models (TMHMM) server (www.cbs.dtu.dk/services/TMHMM-2.0) (Krogh et al., 2001).

The PspA sequences from 123 strains were classified and aligned using the Clustal Omega web server (https://www.ebi.ac.uk/Tools/msa/clustalo/) (Sievers et al., 2011) to identify sequence similarities and differences. The alignment results were edited in BioEdit software version 7.1.3.0 and ambiguous aligned points were corrected. The phylogenetic tree was drawn using Molecular Evolutionary Genetics Analysis (MEGA) software with Maximum likelihood and Neighbor-Joining methods (Saitou and Nei, 1987) to investigate the relationship between different strains. Before computational predictions to design a potential epitope-based vaccine, the Immune Epitope Database (IEDB; https://www.iedb.org/) was used to find the experimentally identified epitopes.

#### 2.1.2 Prediction of conformational (discontinuous) B cell epitopes

Prediction of conformational B cell epitopes requires the three-dimensional structure of a protein. Since there is no 3D structure for PspA and PhtD-C proteins in the PDB database, the 3D models of the proteins were predicted by different servers. These proteins were modeled in the best way using the iterative threading assembly refinement (I-TASSER) server (https://zhanglab.ccmb.med.umich.edu/I-TASSER/) (Yang et al., 2015) or SWISS-MODEL server (http://swissmodel.expasy.org) (Waterhouse et al., 2018). The protein structure was constructed with the help of homology/comparative modeling according to the existence of homologous templates with sequence identity less than 30% using I-TASSER server and sequence identity more than 30% with SWISS-MODEL. The low-quality modeled structures were refined by the servers ModRefiner (https://zhanglab.ccmb.med.umich.edu/ModRefiner/) (Xu and Zhang, 2011) and GalaxyRefine (http://galaxy.seoklab.org/cgi-bin/submit.cgi?type=REFINE) (Shin et al., 2014). The validation of 3D structures was done using PROCHECK (Laskowski et al., 1993) and ERRAT (Colovos and Yeates, 1993) programs in SAVES server (https://saves.mbi.ucla.edu/). BIOVIA Discovery Studio Visualizer was used to display the 3D structure of proteins. Eventually the conformational epitopes were anticipated based on the 3D protein structure using the servers Ellipro (http://tools.iedb.org/ellipro/) (Ponomarenko et al., 2008) and DiscoTope (http://www.cbs.dtu.dk/services/DiscoTope/) (Kringelum et al., 2012). The Ellipro server uses the solvent accessibility and flexibility to identify linear and structural epitopes, and the higher score indicates more solvent accessibility. The DiscoTope server uses half-sphere exposure and propensity scores to identify structural B-cell epitopes.

#### 2.1.3 Prediction of linear B cell and linear T cell epitopes

Unlike conformational epitopes, which are only recognized by B cells, linear epitopes are recognized by both B and T lymphocytes. The linear or continuousB cell epitope is characterized based on properties such as hydrophilicity, accessibility, flexibility and antigenicity in a beta-turn region. In the present study, prediction of linear B-cell epitopes in PspA and PhtD-C proteins were done using web servers LBTope (http://crdd.osdd.net/raghava/lbtope/protein.php) (Singh et al., 2013), ABCpred (http://crdd.osdd.net/raghava/abcpred/) (Saha and Raghava, 2006), IEDB Emini surface accessibility tool (http://tools.immuneepitope.org/bcell/) (Emini et al., 1985) and Ellipro. The LBtope server with a prediction accuracy of 81% and the ABCpred server with an overall accuracy of 65.93% predict linear B cell epitopes. The LBtope assigns a value ranging from 0 to 100% for each predicted epitope, and a higher value indicates a higher probability of being an epitope. In the ABCPred, a score closer to 1 indicates a higher probability that the peptide sequence is an epitope.

Since helper T cells play a role in immunity against extracellular pathogens such as pneumococci, the prediction of CD4+ T cell epitopes (MHC-II binding peptides) was performed using MHC-II prediction tool in IEDB server (http://tools.immuneepitope.org/mhcii) (Wang et al., 2008) and NetMHCIIpan 4.0 server (http://www.cbs.dtu.dk/services/NetMHCIIpan/) (Jensen et al., 2018). To evaluate the immune response against the vaccine candidate in a mouse model, mouse alleles were investigated in addition to human alleles. The predictions were performed for 8 common human alleles HLA-DRB1 (*01:01, *03:01, *04:01, *07:01, *08:01, *11:01, *13:01, *15:01) and mouse alleles H-2-IAb, H-2-IAd and H-2-IEd. The IEDB peptides with percentile rank <10.0 and the NetMHCIIpan peptides with rank value <1.0 were considered for further analysis.

#### 2.1.4 Analysis of epitopic areas of B region of PspA in different strains: to obtain consensus epitope sequences

In the consensus sequence approach, the homologous sequences are analyzed to reach the target sequence. Substitution of amino acids with similar chemical features (such as positively charged Lys & Arg or hydrophobic Leu & Val) often does not significantly affect the structure/function of a protein (Zhang et al., 2013; Jones et al., 2020). The epitopic areas of B region of PspA in different families or clades were evaluated to obtain consensus sequences providing epitopes that facilitate the induction of cross-reactive antibodies. Consensus sequences for each clade or family were obtained using the threshold and majority-based selection methods in BioEdit software. In the threshold-based method, the residue that has a greater frequency than the user’s chosen threshold is selected while in the majority-based method, the most common residue at each position is selected regardless of any threshold. Swiss-Model server was used for modeling of the initial peptide and the final peptide containing the consensus sequences. Superimposition and Root Mean Square Deviation (RMSD) calculations were carried out with UCSF Chimera software (Pettersen et al., 2004) to evaluate possible errors in the models.

#### 2.1.5 Designing peptide-based constructs consisting of PspA and PhtD epitopes

The peptides predicted by different servers consisting of higher scoring B cell epitopes overlapped with immunodominant T-helper (Th) cell epitopes were analyzed and eventually the selected peptide sequences were linked together using flexible linkers (Gly-Gly-Gly-Ser and Gly-Gly-Ser-Ser-Gly-Gly). In order to efficiently separate and preserve the independent folding and immunological activities, flexible linkers were considered to connect adjacent epitopes (Tian et al., 2013). In addition, the 6xHis-Tag was added at the C-terminal of the constructs to aid protein purification. Finally, three constructs were designed and named PAD (rPspA-PhtD, a recombinant fusion peptide consisting of PspA and PhtD epitopes), PA (rPspA, the recombinant peptide consisting of PspA epitopes), PD (rPhtD, the recombinant peptide consisting of PhtD epitopes).

#### 2.1.6 Evaluation of the characteristics of the final constructs

Following designing peptide-based constructs PAD, PA, and PD, their various physicochemical properties, such as molecular weight (MW), total number of positive and negative amino acids, theoretical isoelectric point, instability index, *in vitro*/*in vivo* half-life, average aliphatic index, and average hydropathicity, were evaluated using ProtParam tool in ExPASy server (http://web.expasy.org/protparam/) (Gasteiger et al., 2005). Moreover, antigenicity, solubility, allergenicity, and toxicity were investigated for the designed constructs. The recombinant protein solubility on overexpression in *Escherichia coli* was predicted by SOLpro tool in SCRATCH server (https://scratch.proteomics.ics.uci.edu/) (Magnan et al., 2009). VaxiJen v2.0 (http://www.ddg-pharmfac.net/vaxijen/VaxiJen/VaxiJen.html) (Doytchinova and Flower, 2007) and ANTIGENpro tool of SCRATCH server (http://scratch.proteomics.ics.uci.edu/) (Magnan et al., 2010) were utilized for prediction of the antigenicity of peptides. The servers AllerTOP (http://www.ddg-pharmfac.net/AllerTOP/) (Dimitrov et al., 2014) and AlgPred (https://www.imtech.res.in/raghava/algpred/) (Saha and Raghava, 2007) were used for prediction of the allergenicity of the vaccine candidates. ToxinPred server was employed to predict toxicity of the constructs (https://webs.iiitd.edu.in/raghava/toxinpred/index.html) (Gupta et al., 2013).

#### 2.1.7 Prediction and validation of the tertiary structure of vaccine candidates

3D structure modeling of the protein constructs was performed in the best way by Robetta server (http://robetta.bakerlab.org/) (Kim et al., 2004) based on comparative and ab-initio modeling. The GalaxyRefine server (http://galaxy.seoklab.org/cgi-bin/submit.cgi?type=REFINE) (Shin et al., 2014) was used to refine the structure of the best model. The validation of the models was carried out by PROCHECK (Laskowski et al., 1993) and ERRAT (Colovos and Yeates, 1993) at the SAVES server (https://saves.mbi.ucla.edu/), as well as the ProSA web server (https://prosa.services.came.sbg.ac.at/prosa.php) (Wiederstein and Sippl, 2007).

#### 2.1.8 Identification of conformational B cell epitopes in peptide-based constructs

The structural epitopes which are recognized by antibodies play a major role in the humoral immunity. Therefore, the developed protein constructs should have effective conformational epitopes in their protein domains to induce stronger immunity. The ElliPro server was employed to identify the structural B-cell epitopes in the refined models of the epitope-based vaccine candidates, keeping the default parameters.

#### 2.1.9 Molecular docking of immunogenic peptides with HLA-DRB1_01:01

To confirm the binding affinity of the selected immunogenic regions with MHC-II, molecular docking was performed by ClusPro web server (https://cluspro.org/login.php) (Kozakov et al., 2017). The 3D structures of ≤30-mer peptides corresponding to the selected epitope-rich regions as the ligand and the crystal structure of one of the most common alleles, HLA-DRB1*01:01 (PDB ID: 1AQD), as the receptor were submitted to the server. The docked complexes were visualized by UCSF Chimera software. The PDBsum tool (http://www.ebi.ac.uk/thornton-srv/databases/pdbsum/Generate.html) (Laskowski et al., 2018) was utilized to obtain details of the interactions between the peptides and HLA.

#### 2.1.10 Simulation of the immune response against epitope-based constructs

To evaluate the immune responses to the designed constructs, the immune-simulation was done with the help of the C-ImmSim server (https://kraken.iac.rm.cnr.it/C-IMMSIM/) (Rapin et al., 2010). This server predicts immune interactions in three parts of the mammalian immune system (bone marrow, thymus, and tertiary lymphatic organs), using machine learning techniques and a position-specific scoring matrix (PSSM). Four injections of the construct were performed at 2-week intervals (on days 0, 14, 28, and 42). Each time step is 8 hours and the first injection step is at zero time. The production of cytokines and antibodies as well as the responses of Th1 and Th2 cells were predicted by the server.

#### 2.1.11 Reverse translation, codon optimization and *in silico* cloning of the constructs in pET28a vector

The codon optimization approach was used to enhance the expression of PAD, PA and PD recombinant proteins. The Java Codon Adaptation Tool (JCat) (http://www.jcat.de/) (Grote et al., 2005) online server was used for reverse translation and codon optimization based on codon preference of the expression host *E. coli* (K12 strain). In addition, this server prevents unwanted sites for restriction enzymes, Rho-independent transcription termination, and prokaryotic ribosome binding. Finally, the optimized gene sequences for the PAD, PA, and PD, with restriction sites *Nco*I and *Xho*I respectively added to the 5’ and 3’ ends, were cloned in pET28a vectors using SnapGene tool (https://www.snapgene.com/try-snapgene/).

### 2.2 Experimental verification

#### 2.2.1 *E. coli* transformation, expression and purification of recombinant proteins

The pET28a vector harboring the sequence encoding PAD, PA or PD was synthesized by Biomatik Corporation (Cambridge, Ont., Canada). The recombinant expression vectors (pET28a-PAD, pET28a-PA, and pET28a-PD) were transferred into *E. coli* BL21 (DE3) cells, and recombinant clones were identified by colony PCR using T7 universal primers (https://www.addgene.org/mol-bio-reference/sequencing-primers/). Expression of the recombinant proteins was induced by the addition of 1 mM isopropyl-β-thiogalactopyranoside (IPTG) for 18 h, and examined by running on 15% SDS–PAGE and Coomassie Brilliant Blue staining R-250. The expressed recombinant proteins PAD, PA and PD were purified using Ni-NTA chromatography under native conditions, according to the manufacturer’s manual (Qiagen, Hilden, Germany). Finally, the purified proteins were dialyzed overnight and then quantified by NanoDrop spectrophotometer at 280 nm.

#### 2.2.2 Immunization of mice

**Figure 2:**
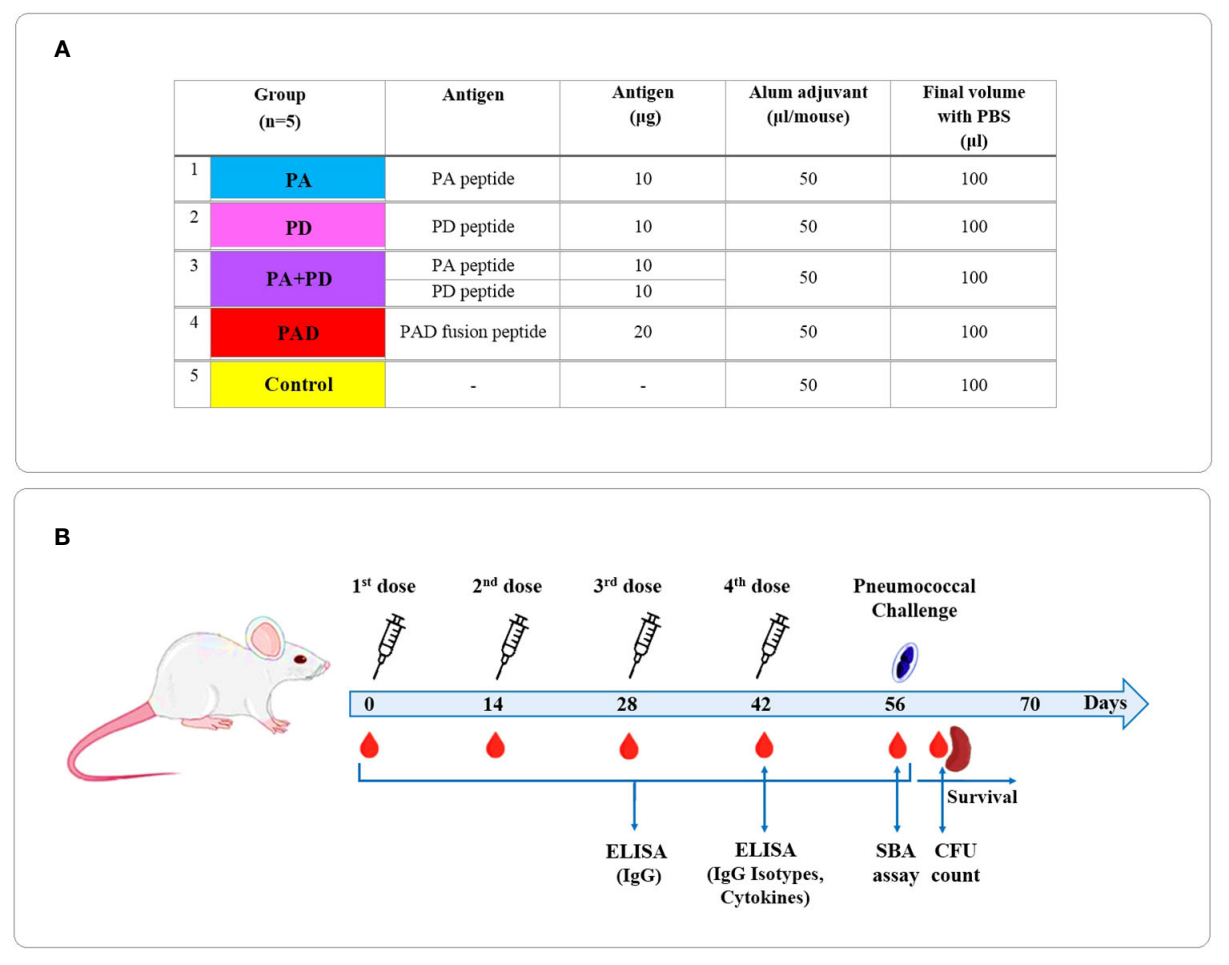
Vaccination schedule and timeline of experimental procedures. **(A)** BALB/c mice were divided into 5 groups PA, PD, PA+PD, PAD, and control (n = 5 per group). **(B)** The mice were immunized subcutaneously on days 0, 14, 28 and 42. The sera were collected before each injection and 2 weeks after the last injection and used for further analysis.

Vaccination schedule and timeline of experimental procedures are presented in **Figure 2**. Six- to eight-week old female BALB/c mice were randomly divided into 5 groups PA, PD, PA+PD, PAD, and control (n = 5 per group). The mice were immunized subcutaneously on days 0, 14, 28 and 42 with 10 μg PA or PD peptide (PA or PD group), 10 μg PA combined with 10 μg PD peptide (PA+PD group), or 20 μg PAD fusion peptide (PAD group) formulated in PBS containing Alum adjuvant (1:1 v/v) in a final volume 100 μl per mouse. The negative control group was injected with the Alum adjuvant in PBS (1:1 v/v). The sera were collected before each immunization and two weeks after the last immunization (on days 0, 14, 28, 42, and 56) and stored for further analysis.

#### 2.2.3 Assessment of the levels of specific total immunoglobulin G (IgG) and IgG isotypes

The levels of anti-recombinant protein IgG on days 0, 14, 28, 42, and 56, and the levels of immunoglobulin G1 and G2a (IgG1 and IgG2a) isotypes two weeks after the 3^rd^ injection were evaluated by indirect enzyme-linked immunosorbent assay (ELISA) (Fereshteh et al., 2023). In brief, ELISA microtiter plates (Nanc MaxiSorp, Thermo Fisher, USA) were coated with 100 μl of purified recombinant protein PA or PD (0.2 μg/well) and incubated at 4 °C overnight. After blocking the plates, 100 μl of diluted anti-sera (1:100) were added to the wells and then the 1:100,000 dilution of HRP-conjugated secondary antibodies (Sigma Chemical, St. Louis, MO, USA) were applied and incubated for one hour. Finally, TMB substrate (Seramun, Dolgenbrodt, Germany) was added to each well and the absorbance was read by an ELISA reader (BioTek Company) at 450 nm.

#### 2.2.4 Evaluation of the levels of cytokines induced by the vaccine candidates

The titers of cytokines interferon-gamma (IFN-γ), interleukin-4 (IL-4), and interleukin-17A (IL-17A) in the mouse sera two weeks after the 3^rd^ injection were determined using ELISA kits (Mabtech, Sweden) in accordance with manufacturers’ guidelines.

#### 2.2.5 Evaluation of serum bactericidal activity

Two weeks after the 4^th^ injection, the SBA assay was used to evaluate the activity of anti-recombinant protein antibodies in killing of pneumococcal strain ATCC 6303. At first the sera of immunized mice were decomplemented at 56°C for 30 min. Then, 12.5 μl of the bacterial suspension at 10^5^ CFU/ml and 12.5 μl of infant rabbit serum as a source of the complement were added to the 96-well microtiter plates containing serially diluted inactivated serum sample (1:2 to 1:64). After 1 h incubation at 37°C in 5% CO_2_, 10 μl of each well was cultured on a blood agar plate and incubated for 24 h at 37°C in 5% CO_2_. The SBA titer was reported as the reciprocal of the serum dilution at which ≥50% of bacteria were lysed in comparison to the negative control without Ab (Fereshteh et al., 2023).

#### 2.2.6 Challenge of immunized mice with *S. pneumoniae*

After the final immunization, challenge was performed intraperitoneally with a lethal dose of pneumococcal strain ATCC 6303 and the mortality of mice was monitored for about one week. One day after the challenge, two mice from each experimental group were euthanized and blood and spleen samples were collected to subject to bacterial colony counting on blood agar plates.

#### 2.2.7 Statistical investigations

GraphPad Prism 8 software was used to perform statistical analysis. Differences in antibody levels were analyzed using two-way ANOVA and cytokine concentration, bacterial loads, and serum bactericidal activity were analyzed using one-way ANOVA followed by Tukey’s multiple comparison test. Kaplan–Meier survival curves was used for comparison between the control and the immunized groups (Guyot et al., 2012). P-values of ≤0.05 were considered significant.

## 3 Results

### 3.1 Immunoinformatics studies

#### 3.1.1 Protein sequences retrieval and initial analysis

The amino acid sequences of the target proteins were extracted from the database for computational epitope mapping. Due to the diversity of the PspA molecule in different strains, PspA amino acid sequences were retrieved from 123 strains in FASTA format. The signal peptide of the proteins was separated from the sequences after prediction using the SignalP server. The TMHMM server results showed that the candidate sequences did not have any transmembrane domain. Following aligning of the sequences of A region of PspAs and drawing the phylogenetic tree, the A region from the reference strain AC122 was selected as a representative to determine epitopes of the A region (**Supplementary Figure S1**, **Supplementary Table S2**). CDR sequences of 123 strains were grouped into clades 1 to 5, and after alignment of the sequences of each clade, the required corrections were made using BioEdit software. Strains with duplicated CDRs were excluded and the remaining strains with non-duplicated CDRs were used for further evaluations (**Supplementary Table S3**). The amino acid sequence of PhtD strain R6 was extracted in FASTA format (**Supplementary Table S4**), and its C-terminal from aa 383 to 853 was chosen for the prediction of epitopes. The investigations showed that there are experimental epitopes for N-terminal of PspA protein, while there is no experimental data for C-terminal of PhtD. The epitopes of the A, B, and C regions of PspAs previously experimentally identified (Daniels et al., 2010; Singh et al., 2010; Vadesilho et al., 2014; Tamborrini et al., 2015) were extracted from IEDB database (**Supplementary Tables S5–S7**, respectively).

#### 3.1.2 Prediction of B-cell and helper T-cell epitopes

Before the prediction of the conformational epitopes, the 3D structure of PspA from strain AC122 was modeled through I-TASSER server and refined with GalaxyRefine server. Validation of the model was performed using ProSA Z-score and the result showed that the Z-score of the structure is -3.88 near to natural proteins of similar size (**Supplementary Figure S2**). Strains DBL6A (Clade1), R6 (Clade2), AC122 (Clade3), EF5668 (Clade4) and ATCC 6303 (Clade5) were selected as representatives of the 123 strains to determine the range of B region epitopes. Tertiary structure modeling of B regions of PspA clades 1-5 was performed using Swiss-Model server based on PMS2 template with sequence similarity more than 30% (**Supplementary Figure S3**). The 3D structure of PhtD-C was predicted through I-TASSER server and refined with ModRefiner and GalaxyRefine servers (**Supplementary Figure S4**). Ramachandran plot demonstrated that 87.2% of amino acids were located in favorable regions, and the ERRAT result showed the overall quality factor was 85.23% (**Supplementary Figure S5**).

The predicted linear/conformational B cell epitopes and MHC-II binding epitopes for A region of PspA are shown in **Supplementary Tables S8-S9**, respectively. For B region of PspAs in clades 1 to 5, B cell epitopes (**Supplementary Tables S10-S14**) and MHC-II binding epitopes (**Supplementary Table S15**) were predicted and considered for further analysis. The predicted linear and structural B-cell epitopes, and MHC class II epitopes for PhtD-C are listed in **Supplementary Tables S16-S18**, respectively. In this way, the epitopes were collected to check and finally select the immune-dominant epitopic regions.

#### 3.1.3 Obtaining the consensus epitope sequences for B region of PspA in different pneumococcal strains

Since the B region of PspA varies in different families/clades, the recognition of consensus epitope sequences capable to cover all strains from each family or clade could be an ideal immunization strategy. These consensus antigens could provide epitopes that facilitate the elicitation of cross-reactive Abs (Nachbagauer and Palese, 2020). For this purpose, the sequences of B region in different families/clades were aligned using Clustal Omega software and the results were edited in BioEdit software. The aligned and modified sequences were evaluated to identify common epitope regions and the 3D structures were considered to select the final suitable domains. As an example, the selected epitope locations of the B region of Clade 1 (strain DBL6A) on the three-dimensional structure are shown in **Supplementary Figure S6**. The selection of consensus epitope sequence of region B of Clade 1 and 2 with the help of BioEdit software is given as an example in **Supplementary Figure S7A**. Since there was overlap between different strains of clades 1 and 2 in the amino acids 1-29 of region B, the consensus sequence was selected for strains of clades 1 and 2 (Cons-Clade 1 + 2; **Supplementary Table S19**). Another consensus sequence of the B region only for strains in Clade 1 is shown as another example in **Supplementary Figure S7B**. Since there was no overlap between strains of clades 1 and 2 in the amino acids 51-76 of B region, the consensus sequence was chosen only for Clade 1 strains (Cons-Clade 1; **Supplementary Table S19**). In the same way, for the other clades, the consensus epitope sequences of B region were selected, and in the area where there was overlap between the strains of two clades, the consensus sequence was considered common (**Supplementary Table S19**).

The initial peptide and the final peptide containing the consensus sequence were modeled using the Swiss-Model server and then structurally checked by the UCSF Chimera program. The superimposition of the primary structure and the structure containing consensus sequence was done to avoid the structural problems of the final peptide (**Supplementary Figure S8**). The RMSD between primary and consensus sequence structures was close to zero, which indicates that despite the amino acid differences there is an overall structural similarity between the peptides and the structure and folding of the resulting antigen is reliable.

#### 3.1.4 Designing epitope-based constructs PAD, PA, and PD

**Figure 3:**
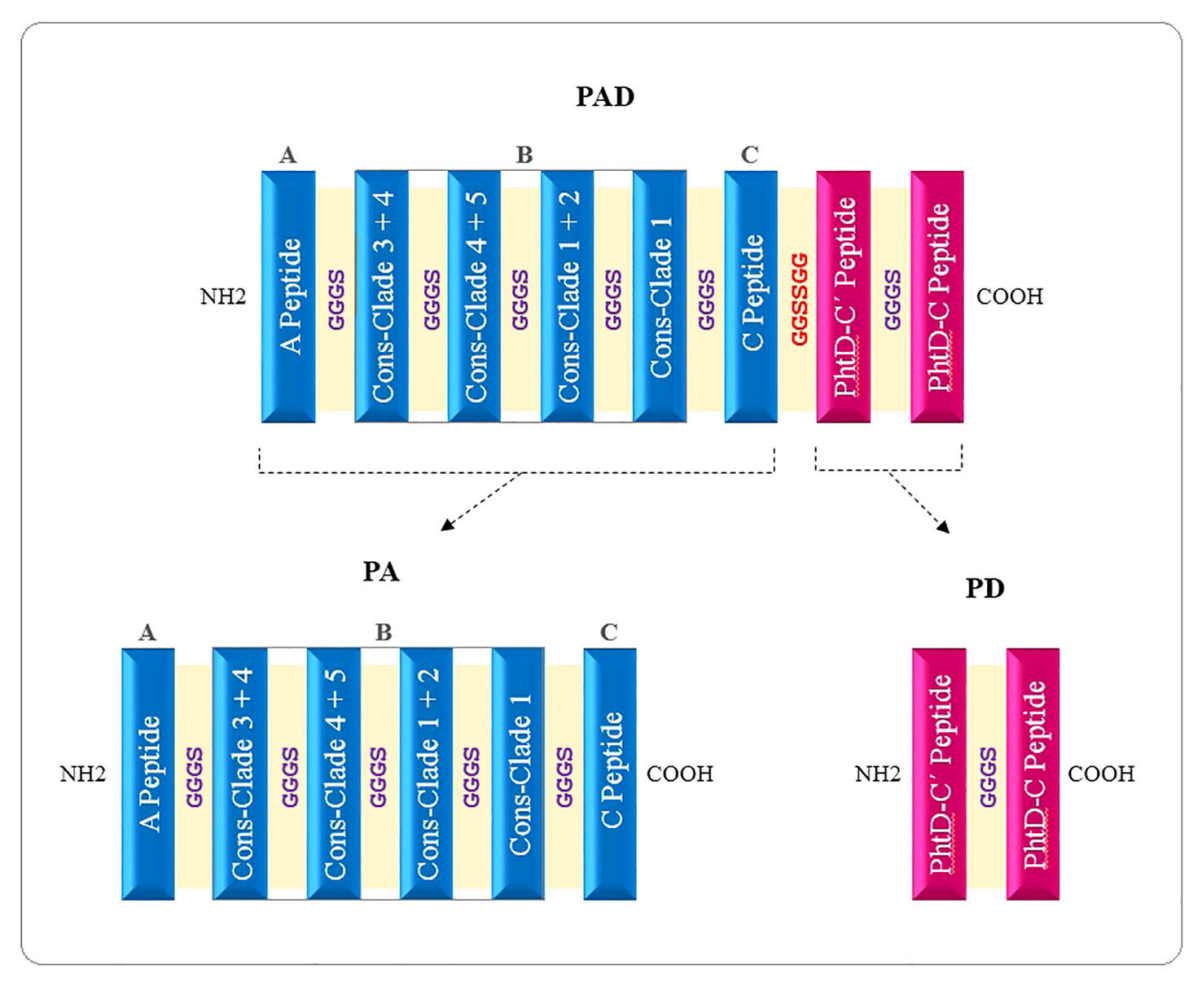
Schematic diagram of vaccine candidates. PAD, fusion peptide consisting of PspA and PhtD epitopes; PA, peptide consisting of epitopes of PspA protein; PD, Peptide consisting of epitopes of PhtD protein.

The high-scoring B- and T-cell epitopes shared between several computational prediction servers were considered to predict immunodominant peptide sequences, and 3D models were analyzed to select the most suitable domains. The selected final peptides from PspA (region A, region B for both families 1 & 2, and region C) and PhtD-C antigens are listed in **Table 1**. Due to the size limitation of the final construct, only four of the seven consensus sequences, which could target different clades, were considered for the B region of the PspA protein. The selected peptides were linked by the linkers GGGS (for peptides related to one protein) and GGSSGG (between peptides related to two proteins PspA and PhtD), which provide the possibility of rotation. The immunodominant peptides were placed next to each other in different positions and the features were evaluated with various servers. Ultimately, the sequences that had the best characteristics among the different positions of the peptides were selected for further analysis. Three final constructs named PAD (fusion construct consisting of PspA and PhtD epitopes), PA (consisting of PspA epitopes) and PD (consisting of PhtD epitopes) were designed. A histidine tag was added by a glycine to the C-terminal end of the designed constructs, which could be useful for protein purification. The schematic picture of how the peptides are placed in the final constructs PAD, PA and PD is shown in **Figure 3**. The total sequence length of PAD, PA, and PD constructs was 329, 222 and 109 amino acids, respectively (**Table 2**).

**Table 1:** Final selected immunodominant peptides of PspA and PhtD for designing the vaccine candidates.

Protein	Antigenic region	Peptide name & sequence	Length
**PspA**	**A Region**	**A Peptide**	
		EAPVASQSKAEKDYDAAVKKSEAAKKHYEEVKKKAEDAQKKYDEGQKKTVEKAKREKEASEKIA	64
	**B Region**	**Cons-Clade 1 + 2**	
		LKEIDESDSEDYIKEGFRVPLQSELDAKR	29
		**Cons-Clade 1**	
		KLEKDVEYFKNTDGEYTEQYLEAAEK	26
		**Cons-Clade 3 + 4**	
		ELEKLLDTLDPEGKTQDELDKEAAEAELDKKV	32
		**Cons-Clade 4 + 5**	
		LTRLEDNLKDAEENNVEDYIKEGLEKAI	28
	**C Region**	**C Peptide**	
		PAPAPKPEQPAPAPK	15
**PhtD**	**C-terminal of PhtD**	**PhtD-C Peptide**	
		SLEDLLATVKYYVEHPNERPHSDNGFGNASDHVQRNKNGQADTNQTEKPNEEK	53
		**PhtD-Cˊ Peptide**	
		EKVTDSSIRQNAVETLTGLKSSLLLGTKDNNTISAEVDSLLALLK	45

**Table 2:** Evaluation of physicochemical and immunological properties of the designed constructs.

Name	Sequence	Num.	MW (kDa)	pI	+R	−R	II	Aliphatic index	GRAVY	Antigenicity (VaxiJen)	Antigenicity (ANTIGENpro)	Solubility (SOLpro)	Allergenicity (AlgPred/ AllerTOP)	Toxicity (ToxinPred)
**PAD**	MEAPVASQSKAEKDYDAAVKKSEAAKKHYEEVKKKAEDAQKKYDEGQKKTVEKAKREKEASEKIAgggsELEKLLDTLDPEGKTQDELDKEAAEAELDKKVgggsLTRLEDNLKDAEENNVEDYIKEGLEKAIgggsLKEIDESDSEDYIKEGFRVPLQSELDAKRgggsKLEKDVEYFKNTDGEYTEQYLEAAEKgggsPAPAPKPEQPAPAPKggssggEKVTDSSIRQNAVETLTGLKSSLLLGTKDNNTISAEVDSLLALLKgggSLEDLLATVKYYVEHPNERPHSDNGFGNASDHVQRNKNGQADTNQTEKPNEEKgHHHHHH	329	35.69	5.05	49	70	39.88	61.76	-1.148	1.0069	0.887445	0.918234	non-allergenic	non- toxic
**PA**	MEAPVASQSKAEKDYDAAVKKSEAAKKHYEEVKKKAEDAQKKYDEGQKKTVEKAKREKEASEKIAgggsELEKLLDTLDPEGKTQDELDKEAAEAELDKKVgggsLTRLEDNLKDAEENNVEDYIKEGLEKAIgggsLKEIDESDSEDYIKEGFRVPLQSELDAKRgggsKLEKDVEYFKNTDGEYTEQYLEAAEKgggsPAPAPKPEQPAPAPKgHHHHHH	222	24.34	5.00	38	54	49.82	56.40	-1.285	1.0392	0.689983	0.896574	non-allergenic	non- toxic
**PD**	MEKVTDSSIRQNAVETLTGLKSSLLLGTKDNNTISAEVDSLLALLKgggSLEDLLATVKYYVEHPNERPHSDNGFGNASDHVQRNKNGQADTNQTEKPNEEKgHHHHHH	109	11.97	5.94	11	16	15.6	71.56	-0.982	0.7390	0.893044	0.876282	non-allergenic	non- toxic

The linker gggs for peptides related to one protein and the linker ggssgg between peptides related to two proteins (PspA and PhtD) are shown in blue and red, respectively.

#### 3.1.5 Evaluation of the various properties of the designed constructs

The designed constructs were evaluated and compared with respect to their physicochemical and immunological properties. Various features of the constructs were predicted using the ProtParam tool as represented in **Table 2**. The molecular weight of PAD, PA, and PD vaccine constructs was 35.66, 24.34, and 11.94 kDa, with theoretical pI about 5.05, 5.00, and 5.94, respectively. The instability index of PAD, PA, and PD were predicted as 39.88, 49.82, and 15.6, respectively. Proteins with an instability index below 40 are taken into account stable and vice versa (Gasteiger et al., 2005). For epitope-based proteins PAD, PA, and PD, the aliphatic index respectively was 61.76, 56.40 and 71.56 (indicating that they could be thermostable), and the GRAVY index respectively was -1.148, -1.285, and -0.982 (indicating that they are hydrophilic). The half-life of the protein constructs was calculated to be 30 hours in *E. coli*, more than 20 hours in yeast and more than 10 hours in mammals. Moreover, the antigenicity, solubility, allergenicity and toxicity of the constructs were investigated as shown in **Table 2**. The sequences were analyzed by VaxiJen and ANTIGENpro servers, and the scores above the threshold indicated that the proteins were antigenic in nature. Based on the prediction of the solubility upon overexpression in *E. coli*, the designed recombinant proteins were soluble. AlgPred and AllerTOP servers demonstrated that the protein constructs were non-allergen. The ToxinPred server showed the non-toxicity of the constructs for humans and animals. The output of these computational analyses revealed that the proposed constructs passed the evaluations with satisfactory results.

#### 3.1.6 Modeling and quality assessment of 3D structures of the constructs

**Figure 4:**
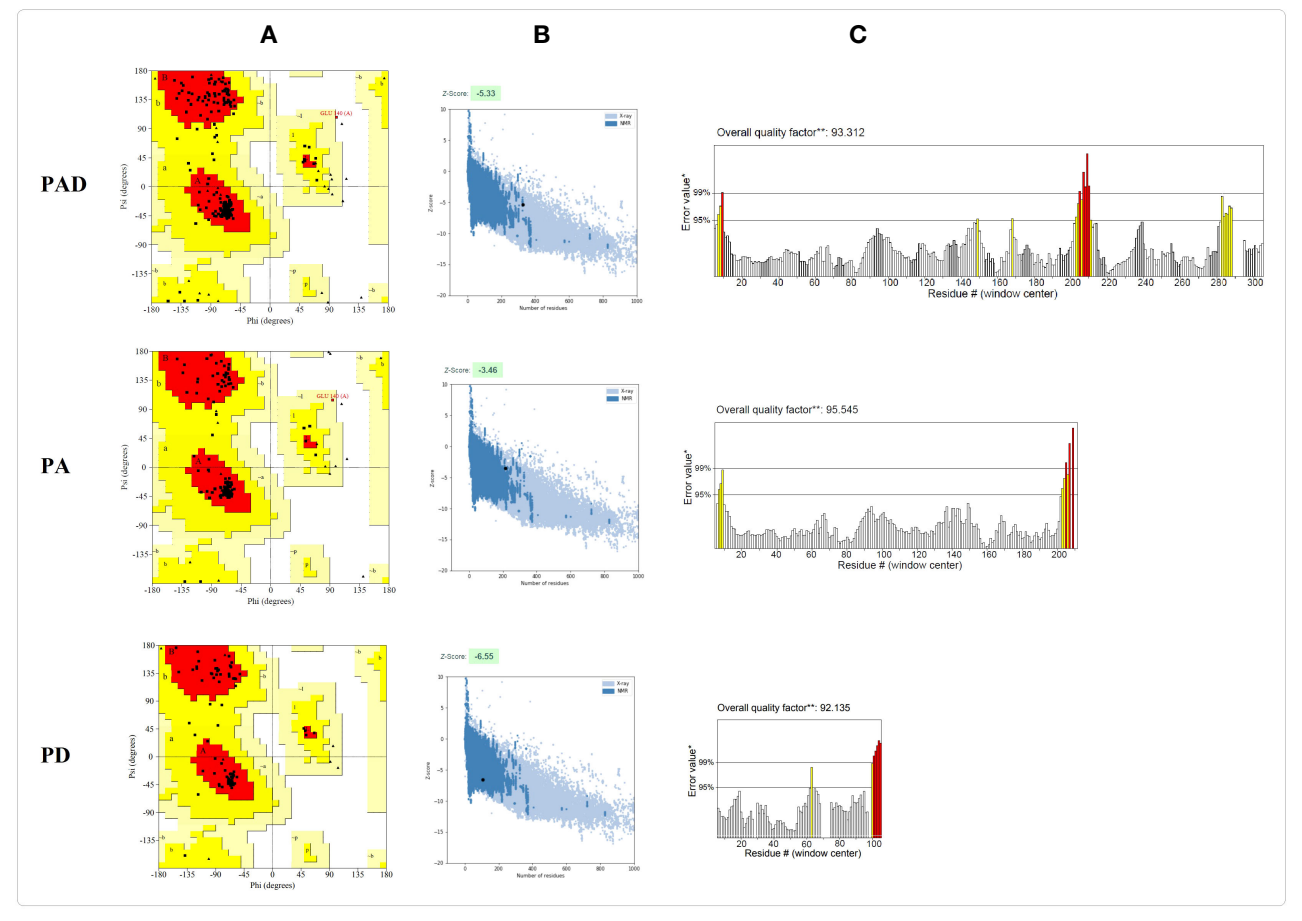
Validation of the final models PAD, PA, and PD after refinement. **(A)** The Ramachandran plots show that in the refined models PAD, PA, and PD, 92.5%, 95.6% and 90.3% of the residues are located in the favorable areas, respectively. The most favored, allowed, generously allowed and disallowed regions are shown in red, dark yellow, lighter yellow and white, respectively. **(B)** The ProSA Z-scores are found to be -5.33, -3.46, and -6.55 in the refined models PAD, PA, and PD, respectively. The z-scores of proteins determined by NMR or X ray are represented in dark or light blue, respectively. **(C)** In the ERRAT plots, the overall quality factors of the refined structures PAD, PA, and PD are 93.31, 95.54, and 92.13, respectively. The red and yellow colours show the problematic parts while the white colour shows the normal parts in the structure.

The three-dimensional structures of the protein constructs PAD, PA, and PD were predicted by Robetta server and refined by the GalaxyRefine server. The quality evaluation of the models was accomplished using Ramachandran plot, ERRAT value and ProSA Z-score. The predicted 3D structures and validation of the models before and after refinement are represented in **Table 3**. The Ramachandran, ProSA Z-score, and ERRAT plots before refinement and after refinement are depicted in **Supplementary Figure S9** and **Figure 4**, respectively. The Ramachandran plots demonstrated that in the primary models PAD, PA, and PD, 89.6%, 92.3,% and 84.9% of residues were in the favored regions, while in the refined models, 92.5%, 95.6% and 90.3% of residues were in the favored regions, respectively. The ERRAT values of the crude models PAD, PA, and PD were predicted as 93.96, 95.14, and 89.24, while the values of the refined models were calculated as 93.31, 95.54, and 92.13, respectively (higher score represents the higher quality). The Z-scores obtained by the ProSA were found to be -5.38, -3.48, and -6.63 in the primary models PAD, PA, and PD, compared to -5.33, -3.46, and -6.55 in the refined models, respectively (within the range of scores for natural proteins). The modeled and validated structures were used for further analysis.

**Table 3:** Predicted 3D structures and quality evaluation of the models before refinement (B.R.) and after refinement (A.R.).

Name	Structure	Step	Ramachandran plot			ProSA Z-Score	ERRAT
			Most favored regions (%)	Allowed regions (%)	Disallowed regions (%)		
**PAD**	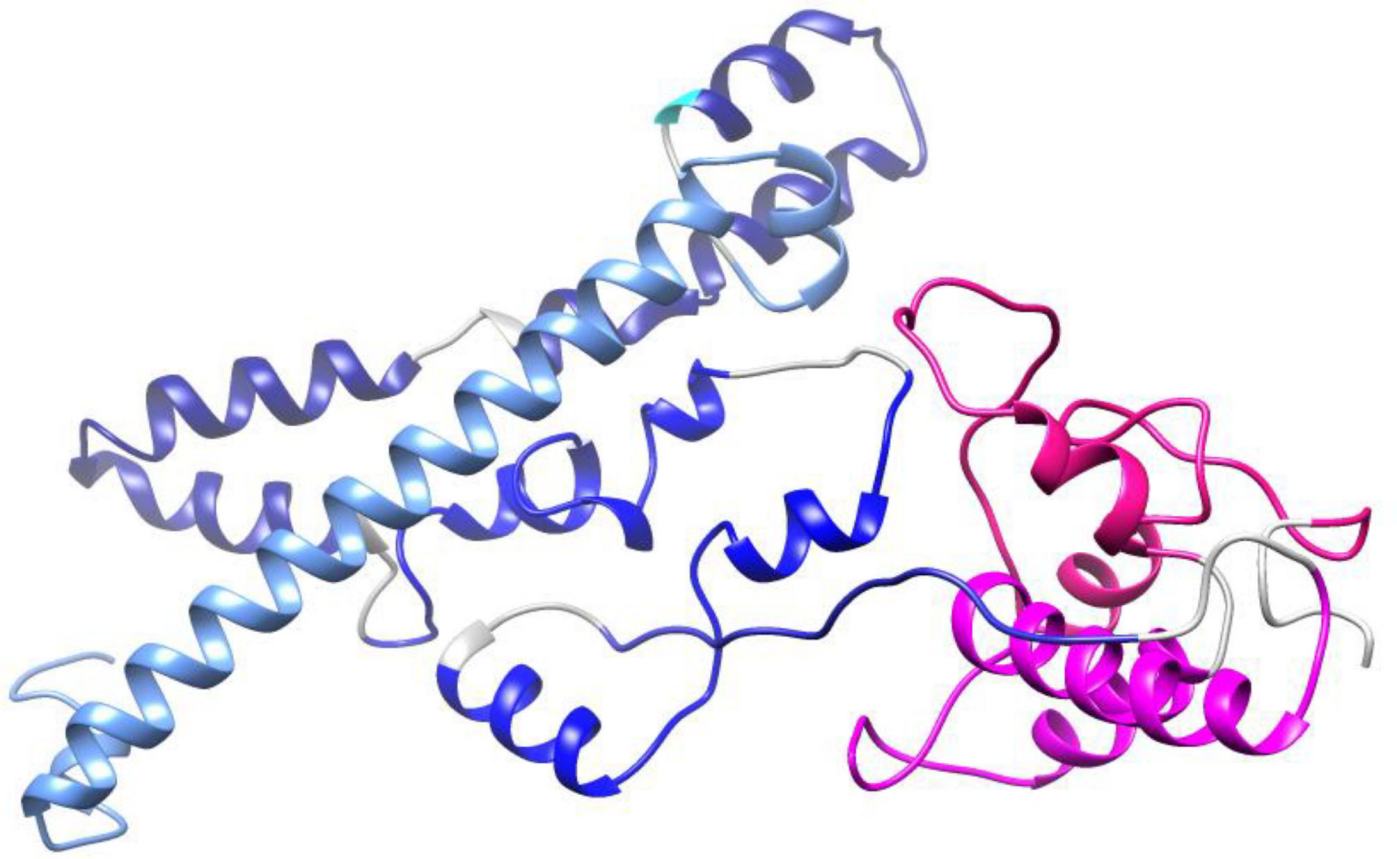	B.R.	89.6	10.4	0.0	-5.38	93.96
		A.R.	92.5	7.1	0.4	-5.33	93.31
**PA**	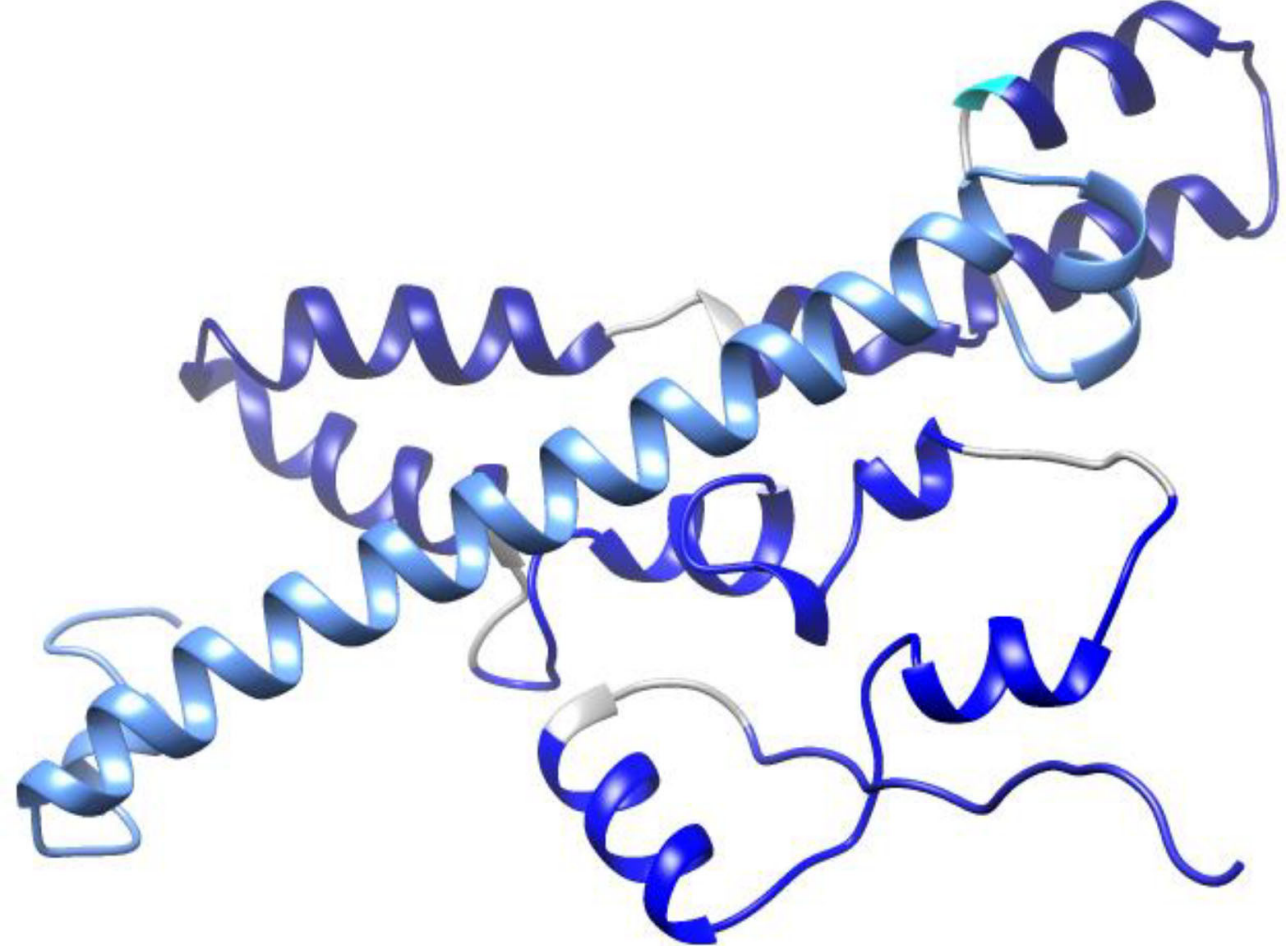	B. R.	92.3	7.7	0.0	-3.48	95.14
		A.R.	95.6	4.3	0.0	-3.46	95.54
**PD**	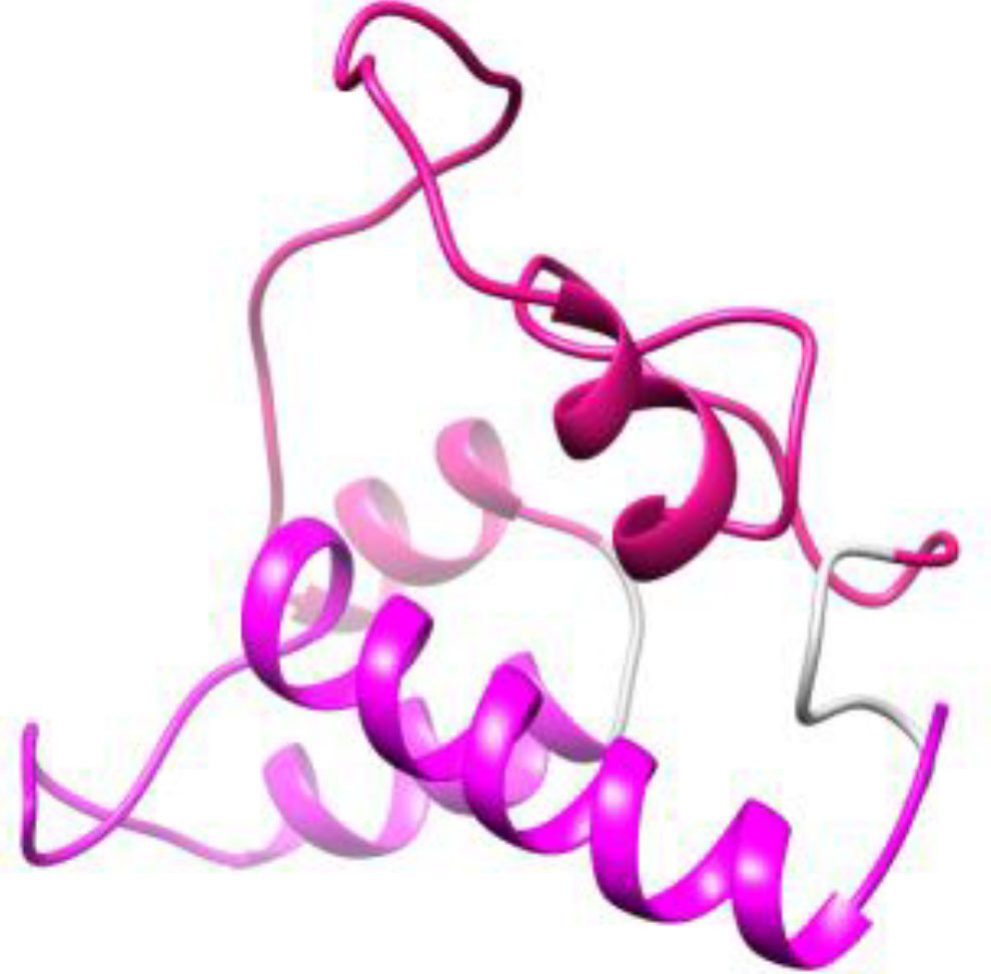	B.R.	84.9	15.1	0.0	-6.63	89.24
		A.R.	90.3	9.7	0.0	-6.55	92.13

#### 3.1.7 Prediction of structural B cell epitopes in engineered constructs

**Figure 5:**
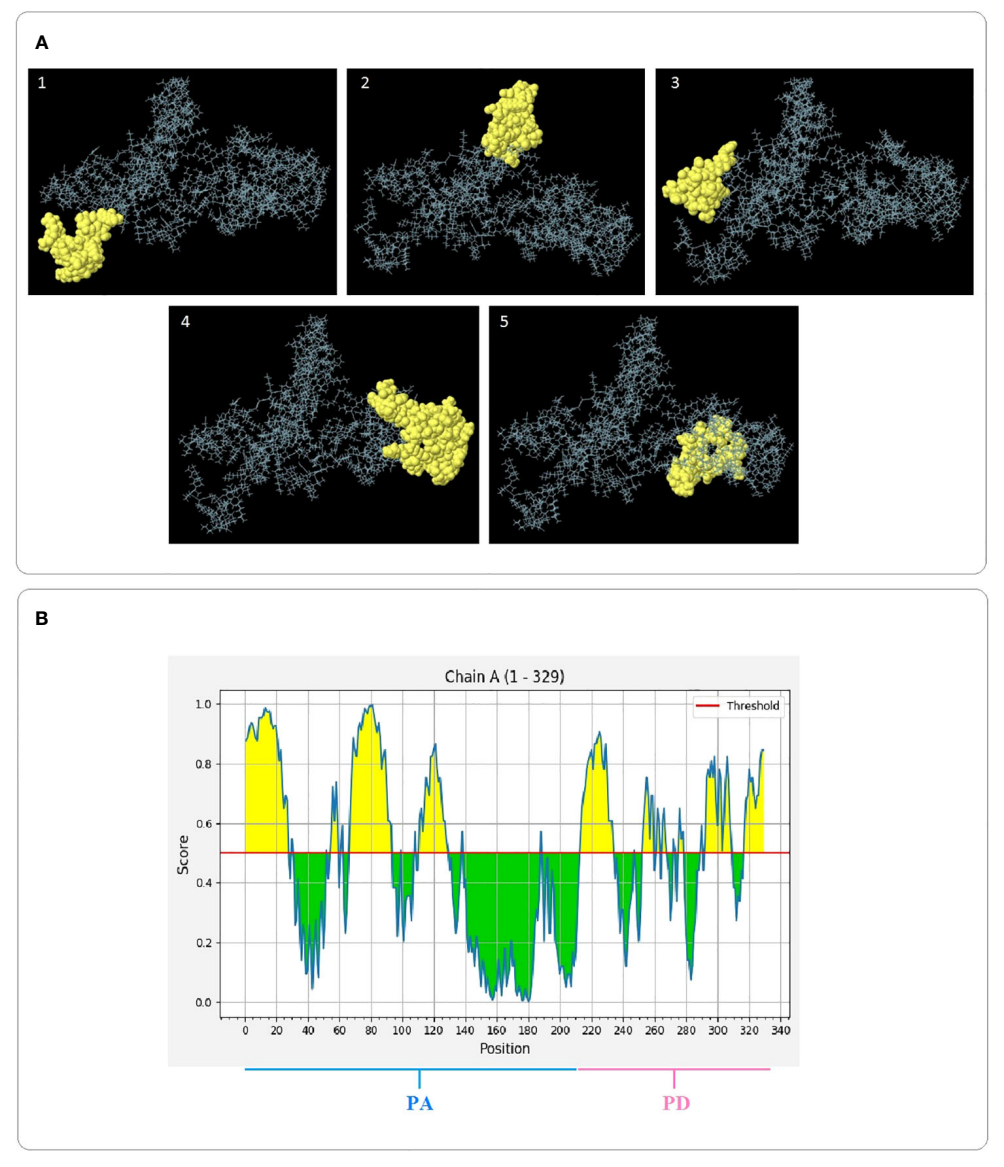
3D images of structural B cell epitopes of the PAD construct and 2D score chart. **(A)** The yellow and gray areas represent the structural epitopes and the other protein segments, respectively. **(B)** Yellow areas with values ​​above a threshold of 0.5 indicate potential B-cell epitopes.

Since most of B cell epitopes are discontinuous, the 3D models of the final designed constructs were analyzed for identification of the conformational epitopes by the ElliPro server. It was found that the PAD construct possessed five new epitopes, comprising 20 to 63 residues with the score values of 0.531 to 0.887 (protrusion index). The PA construct had five new epitopes, comprising 4 to 39 residues with the score values of 0.592 to 0.792. For the PD construct, three new epitopes, comprising 19–22 residues were found with a score value of 0. 636 –0.717. The details of the identified structural epitopes of the constructs PAD, PA, and PD are given in **Supplementary Table S20**. The three-dimensional representation of the epitopes in the PAD, PA, and PD constructs are shown in **Figure 5**, and **Supplementary Figures S10-S11**, respectively.

#### 3.1.8 Further validation using molecular docking with HLA

**Figure 6:**
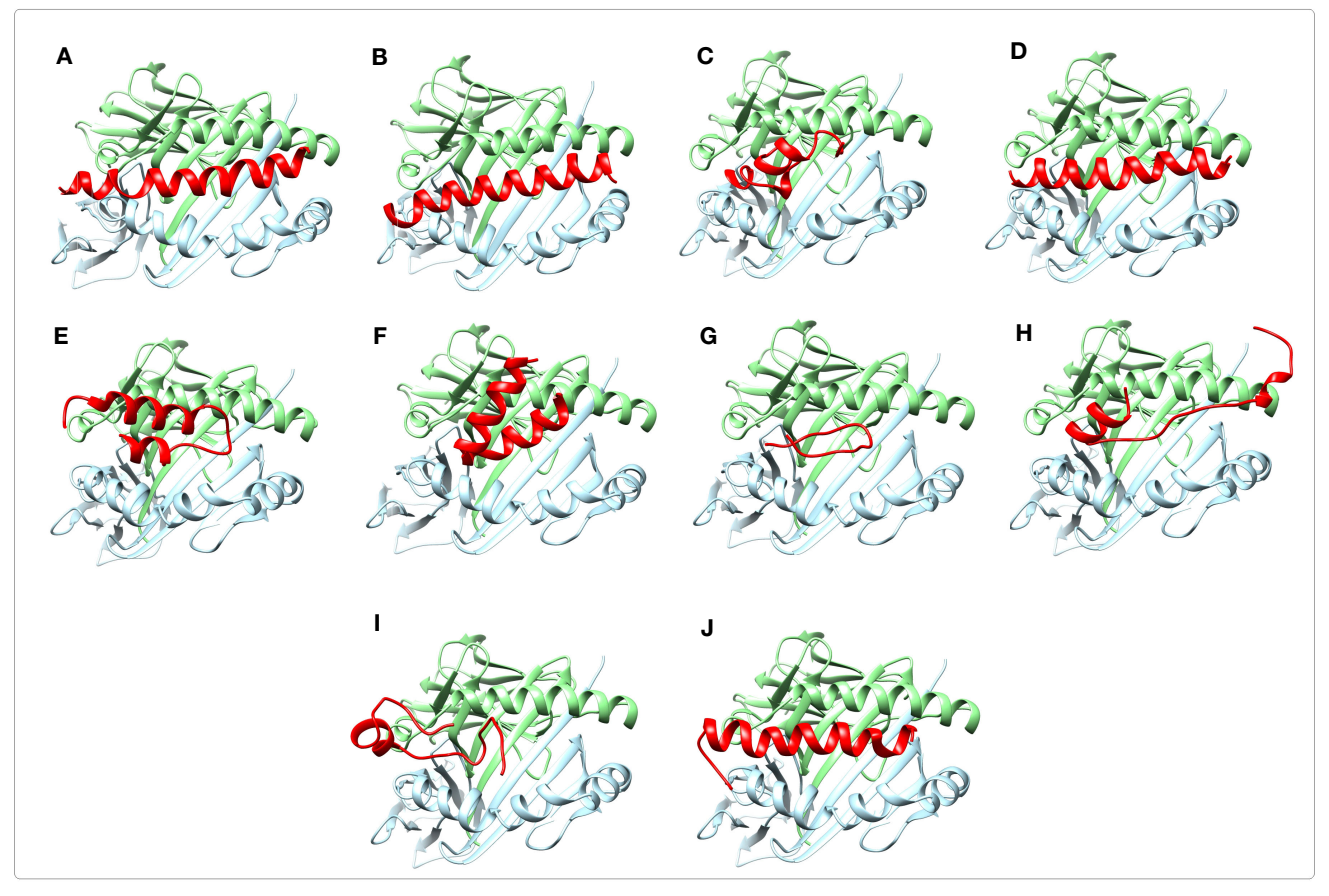
Molecular docking of immunogenic peptides with HLA-DRB1_01:01 molecule (chains A and B). Schematic images of peptide-HLA complexes were shown using Chimera software. The ≤30-mer peptides from the selected epitope-rich regions are shown in red. The A and B chains of HLA molecule are indicated in light green and light blue colors, respectively. The results displayed that the peptides had a favorable interaction with the receptor groove. **(A, B)** The peptides are related to the selected immunogenic part of A region of PspA. **(C-F)** The peptides are the selected B regions of PspA. **(G)** The peptide is the selected C region of PspA. **(H-J)** The peptides are related to the selected immunogenic regions of C-terminal of PhtD.

The selected immunogenic regions were further validated using molecular docking with MHC Class II (DRB1*01:01) receptor. Since the optimal size of peptides binding to the cleft of MHC-II molecules is approximately 30 residues, the ≤30-mer peptides of the selected epitope-rich regions were considered for the molecular docking. Following the docking process, the complex models ranked according to the energy level were generated by the ClusPro 2.0 server. The best docked complexes were visualized by Chimera software as shown in **Figure 6**. According to the results it appeared that the peptides interacted favorably with the receptor groove. For each complex, the lowest interaction energy scores, the number of salt bridges, hydrogen bonds, and non-bonded contacts formed between the peptides and the receptor are given in **Supplementary Table S21**.

#### 3.1.9 Immune simulations

**Figure 7:**
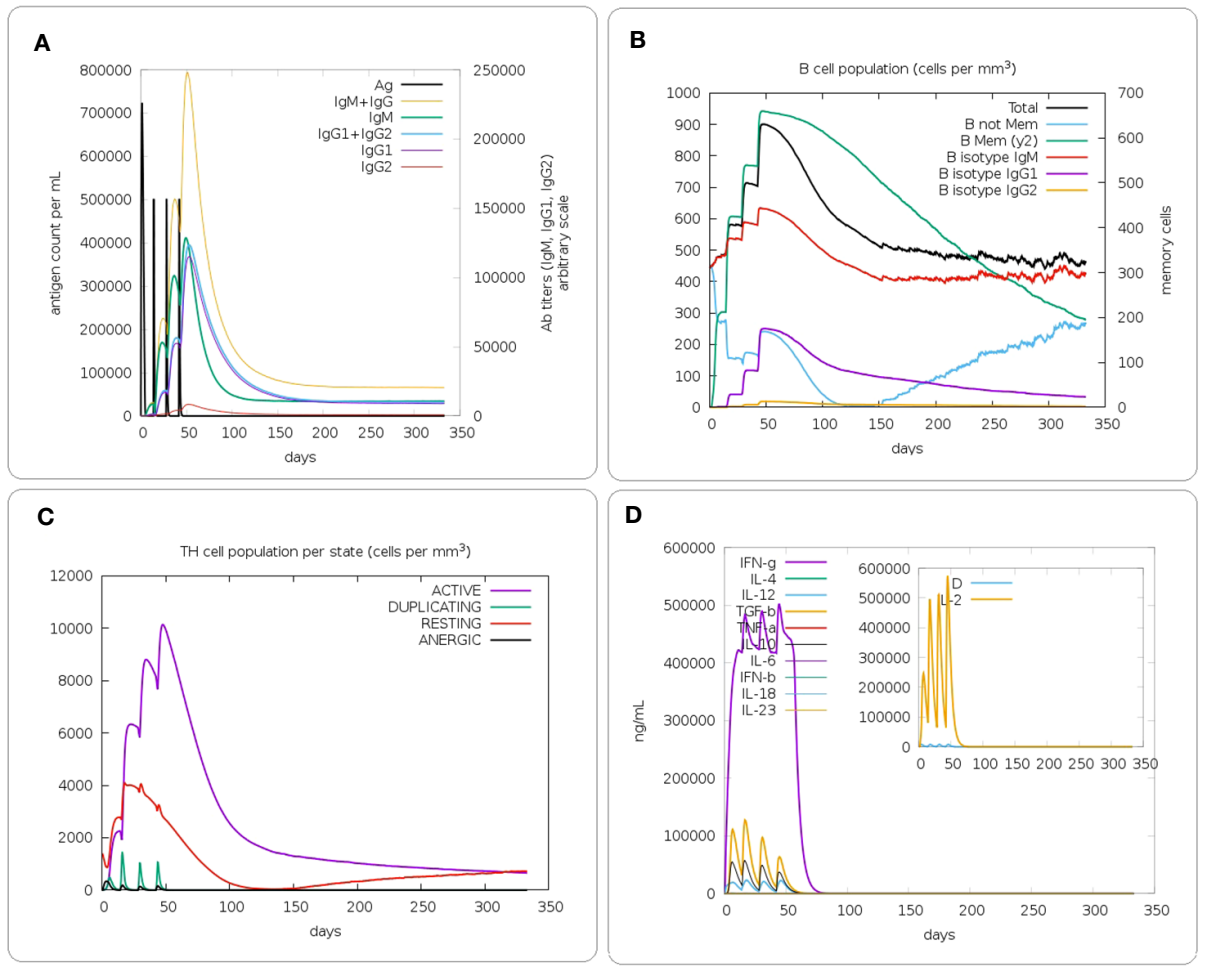
*In silico* immune simulation of the PAD construct. **(A)** Immunoglobulin production in response to injection of the PAD construct (antigens and immunoglobulin subclasses are represented as black and colored peaks, respectively). **(B)** Evolution of the B cell population after 4 injections. **(C)** Evolution of the T-helper cell population. **(D)** Analysis of the levels of different cytokines induced by the construct. The cytokines were distinguished by different colours and the concentration was expressed as ng/ml.

*In silico* immune simulations were performed for the evaluation of immunogenic profile of the constructs in real life. Based on the immune-simulation results, the designed constructs are able to generate appropriate primary, secondary and tertiary responses (**Figure 7** and **Supplementary Figures S12-S13** respectively for the PAD, PA and PD constructs). After the first injection, the initial response was characterized by an increase in the titer of IgM antibodies. After the injection of the booster dose, secondary and tertiary responses were characterized by increased B cell population, isotype switching, and memory cell formation, as well as increased immunoglobulin expression (IgM, IgM + IgG, and IgG1 + IgG2). Moreover, an increase in T helper cells with the development of memory was observed. The Immune simulations also demonstrated that the immunizations were able to induce production of IFN-γ which increases the activity of macrophages.

#### 3.1.10 Reverse translation and *in silico* cloning of the constructs

To achieve a high-level expression of recombinant proteins, the codon optimization of the designed constructs was done using the JCat server based on the codon preference of *E. coli* strain K12. Codon optimization can increase protein production by affecting mRNA stability. The CAI values for the optimized sequences of *PAD*, *PA* and *PD* were 0.98, 0.98 and 1.0, respectively. As much as this number is close to 1.0, it indicates a high level of expression in the bacterial system. The predicted GC contents for *PAD*, *PA* and *PD* were 47.82%, 46.54% and 51.07%, respectively, which are in the optimum range (30-70%) and show the possibility of high expression of the constructs in *E. coli* strain K12. The *Nco*I (CCATGG) and *Xho*I (CTCGAG) cleavage sites were inserted into the 5’ and 3’ ends of the optimized *PAD*, *PA* and *PD* sequences, respectively (**Supplementary Table S22**). The total length of the gene sequences of *PAD*, *PA* and *PD* were 998, 677 and 338 nucleotides, respectively. Finally, the adapted vaccine sequences were placed in the expression vectors pET28a(+) between the restriction enzymes *Nco*I and *Xho*I using the SnapGene tool. The developed plasmids were designated as pET28a-PAD, pET28a-PA, and pET28a-PD plasmids (**Supplementary Figures S14**).

### 3.2 Experimental analysis

#### 3.2.1 Expression and purification of recombinant epitope‐based proteins PAD, PA, and PD

**Figure 8:**
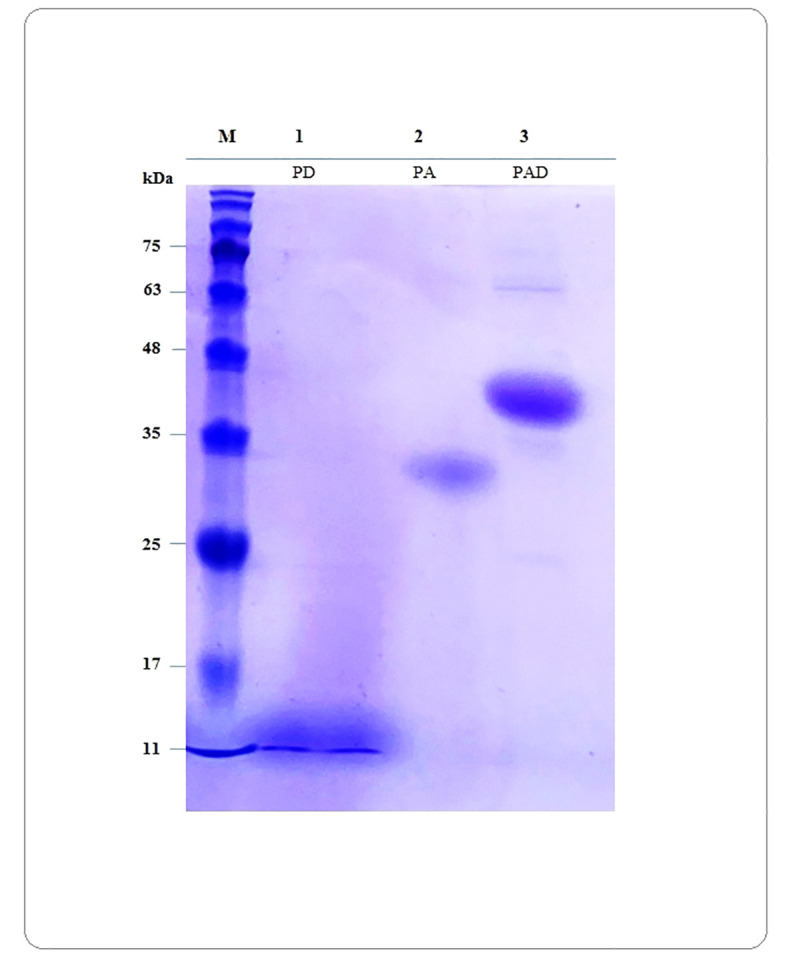
Verification of presence of purified proteins by SDS-PAGE electrophoresis. The calculated MW for the peptide constructs PAD, PA and PD are 35.69, 24.34 and 11.97 kDa, respectively. The calculated MW of PAD and PA is lower than the MW observed on the gel, indicating these proteins move more slowly. Lane M: protein marker; Lanes 1 to 3: Proteins PD, PA and PAD, respectively.

The optimized sequences of *PAD*, *PA* and *PD* were synthesized and cloned into the *Nco*I and *Xho*I restriction sites of pET28a plasmids by Biomatik Company (Canada). The recombinant plasmids were transferred into *E. coli* BL21 and the positive clones were detected by colony PCR using T7 universal primers (**Supplementary Figures S15**). The protein expression was successfully performed in *E. coli* and the protein purification was carried out by Ni-NTA chromatography under native conditions. As shown in **Figure 8**, the presence of proteins was monitored by SDS-PAGE, although the proteins PAD and PA showed a little different mobility on the gel. The calculated MW of PAD and PA was lower than the MW observed on the SDS-PAGE gel, indicating that these proteins move more slowly. These differences can be caused by the different protein characteristics such as the amino acid composition (high percentage of acidic AA), increased hydrophilicity (lower GRAVY), or the presence of proline in the protein. It has been found that the discrepancy between the calculated and observed MW on the gel reveals a linear relationship with the content of acidic residues, glutamate (Glu) and aspartate (Asp), that fits the equation y= 276.5x-31.33 (x presents the content of Glu and Asp and y presents the average ΔMW per residue) (Guan et al., 2015). The PA and PAD proteins consist of 222 and 329 residues with 24.32% and 21.27% acidic residues, respectively. Using the mentioned equation, it may be possible to conclude that the MW of PA and PAD proteins on SDS-PAGE gel is approximately 8 and 9 kDa higher than their predicted MW. In the next step, the purified recombinant proteins PAD, PA, and PD were used for subsequent *In vivo* immunogenicity assessments in mice.

#### 3.2.2 Specific total IgG responses induced by the vaccine candidates

**Figure 9:**
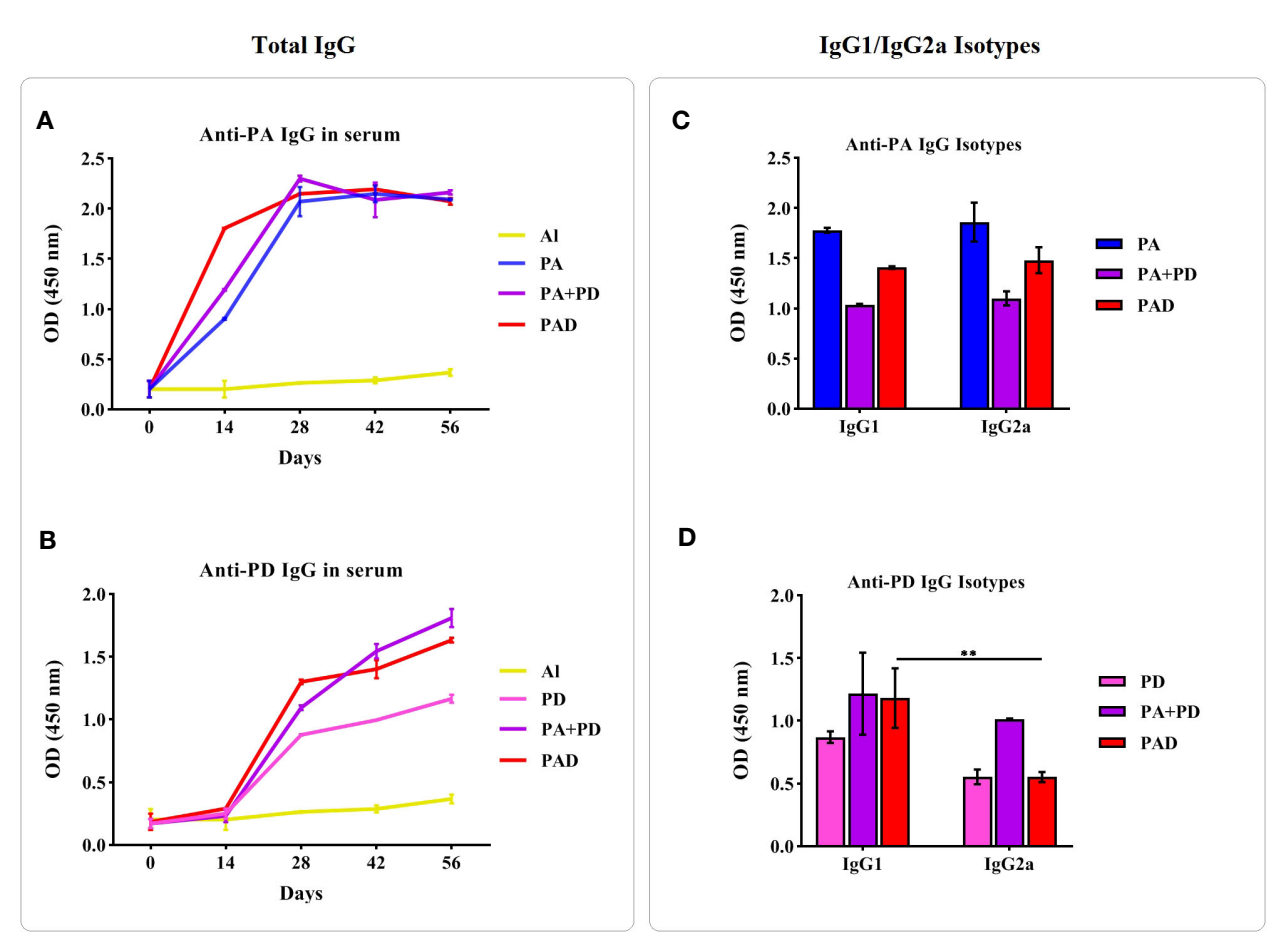
Immunogenicity of the recombinant proteins. Left figures: The comparison of specific total IgG responses in test and control groups was done before each injection and 2 weeks after the last injection (days 0, 14, 28, 42, and 56). Mice (n = 5 per group) were vaccinated with PA or PD alone, PA+PD, PAD or Al adjuvant, and then antisera were examined against PA or PD separately. **(A)** Anti-PA responses in the groups PA, PA+PD and PAD were significantly increased after the second, third, or fourth immunization compared to the first immunization. **(B)** Anti-PD responses in the groups PD, PA+PD and PAD after each immunization showed significant increases compared to the preceding phase. Right figures: The responses of IgG isotypes against the peptide PA or PD in the tested mice groups (n = 5) were examined 2 weeks after the third injection. **(C)** Specific IgG1 and IgG2a subclasses against the PA peptide in the groups PA, PA+PD and PAD did not show significant differences from each other. **(D)** The IgG1 subclass against the PD peptide showed 0.6-fold increase compared to IgG2a subclass in the PAD group. Statistical analysis was done using two-way ANOVA. **p < 0.01.

After the injection with the PAD fusion peptide, the mixture of PA and PD, and PA or PD alone, the levels of specific IgG produced against PA or PD were evaluated by indirect ELISA method. The **Figures 9A, B** shows IgG titers at 2 weeks after every 4 injections, i.e. on days 14, 28, 42, and 56, in the test mice groups compared to each other and the control group (Al). The results of anti-PA IgG responses (**Figure 9A**) showed that 2 weeks after the first injection on day 14, the groups PA, PA+PD and PAD had significant increases in the Ab levels compared to the control group. Also on day 14, the PA+PD and PAD groups exhibited 0.2- and 0.9-fold increases in the levels of anti-PA IgG compared to the PA group, respectively. In addition, there was a difference in term of anti-PA IgG level on day 14 between the PA+PD and PAD groups with an average of 0.6, indicating the superiority of the PAD group. Anti-PA IgG levels on day 28 had significant differences compared to the levels on day 14, while no significant differences were observed between days 28, 42, and 56 in the test groups.

The data of anti-PD IgG responses (**Figure 9B**) demonstrated that 2 weeks after the second injection on day 28, the groups PD, PA+PD and PAD had significant increases in the Ab levels compared to the control group. Also on day 28, the PA+PD and PAD groups showed a 0.2- and 0.4-fold increase in anti-PD IgG responses compared to the PD group, respectively. There was a slight difference in term of anti-PD IgG level among the PA+PD and the PAD groups which showed the superiority of the PAD group. Anti-PD responses on day 42 showed an increase compared to the responses on day 28 only in the PA+PD group. On day 56 compared to day 42, there was increases in the anti-PD IgG levels in the groups PD, PA+D and PAD. These results showed that the antibody responses against PD were increased after the second injection, and these increases continued following the next injections.

#### 3.2.3 Assessment of IgG subclasses

To determine the induction of Th1/Th2 immune responses, the levels of specific IgG1 and IgG2a against PA or PD were evaluated two weeks after the third injection. No significant differences were observed between IgG1 and IgG2a anti-PA antibodies in the mice groups PA, PA+PD and PAD (**Figure 9C**). Also, no significant differences were observed between IgG1 and IgG2a anti-PD antibodies in the PD and PA+PD groups, while in the PAD group a significant increase was observed in the level of IgG1 compared to IgG2a (**p < 0.01; **Figure 9D**). The IgG1/IgG2a ratio against PA in the groups PA, PA+PD and PAD is almost equal to one, showing the induction of a balanced Th1 and Th2 response (**Supplementary Table S23**). The ratio of anti-PD IgG isotypes in the groups PD, PA+PD and PAD is more than one, indicating the dominance of Th2 immune response (**Supplementary Table S23**).

#### 3.2.4 Measurement of cytokine responses

**Figure 10:**
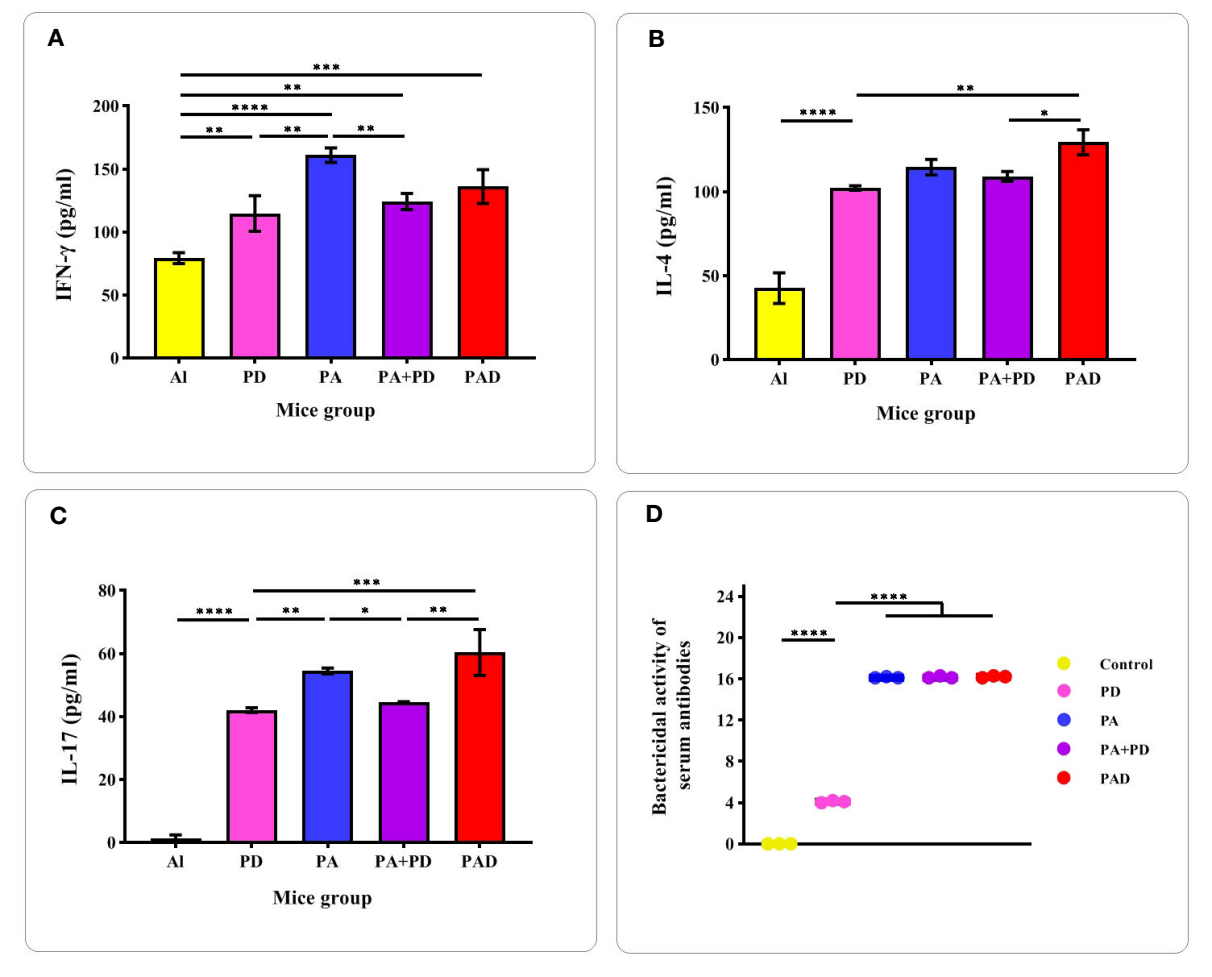
Evaluation of the levels of cytokines and the bactericidal activity of antibodies. **(A-C)** Measurement of the cytokines IFN-γ, IL-4, and IL-17 in test and control mice (n = 5 per group) was performed 2 weeks after the 3th injection. **(D)** The complement-mediated bactericidal activity of anti-recombinant proteins antibodies was evaluated 2 weeks after the 4th injection (n = 3). The Y-axis is the reverse of the dilution of serum in which more than 50% of the bacteria are killed compared to the control. The activity of antibodies in the sera of PA, PAD and PA+PD groups was higher than that in the serum of PD group. There was no significant difference in the serum activity of PA, PAD and PA+PD groups. *p < 0.05, **p < 0.01, ***p < 0.001 and ****p < 0.0001.

To confirm the induction of Th1/Th2/Th17 responses, evaluation of the levels of cytokines IFN-γ, IL-4 and IL-17 in the serum of mice groups PD, PA, PA+PD, PAD and control Al was done two weeks after the third immunization by sandwich ELISA method. As shown in the **Figure 10A**, the mice groups PD, PA, PA+PD and PAD showed ~1.4-, 2-, 1.5-, and 1.7-fold increases in IFN-γ levels compared to the control group, respectively. The levels of IFN-γ in the PA group were increased by 1.4- and 1.2-fold compared to those in the PD and PA+PD groups, respectively. However, no significant differences were observed between the PA and PAD groups, as well as between the PA+PD and PAD groups. From the results it follows that Th1 cell responses could be activated by subcutaneous immunization with PA, PD (individually as well as in combination) and PAD proteins.

As illustrated in **Figure 10B**, the groups PD, PA, PA+PD and PAD showed significant (more than 2.5-fold) increases in IL-4 levels compared to the control group. The superiority of the fusion peptide group among the test groups was observed in terms of IL-4 production. PAD group showed about more than 1-fold increase in IL-4 level compared to PD and PA+PD groups. As seen in **Figure 10C**, the cytokine IL-17A also showed significant increases in all test groups compared to that in the control group. Among the test groups, the superiority of the fusion group was also observed in terms of IL-17A production. The level of IL-17A in the PAD fusion group showed an increase of more than 1.3-fold compared to that in the PD and PA+PD groups. According to the results, it was found that the constructs PA, PD (individually and in combination) and PAD could activate Th2 and Th17 responses, and among them, the fusion peptide could enhance these responses more favorably.

#### 3.2.5 Antibody-dependent bactericidal assay

The complement-mediated bactericidal activity of antibodies created against the recombinant proteins in the serum of mice groups PA, PD, PA+PD and PAD was evaluated by SBA assay 2 weeks after the 4th immunization (**Figure 10D**). The specific antibodies in the serum of PA, PA+PD and PAD mice groups were more effective than those in serum of the PD group. The bactericidal activity of antibodies in the serum of PD group was considered in 1:4 dilution in which approximately 50% of the bacteria are killed compared to the control. The bactericidal activity of the serum of groups PA, PAD and PA+PD was considered in 1:16 dilution.

#### 3.2.6 Analysis of mice survival rates and bacterial loads in the blood and spleen

**Figure 11:**
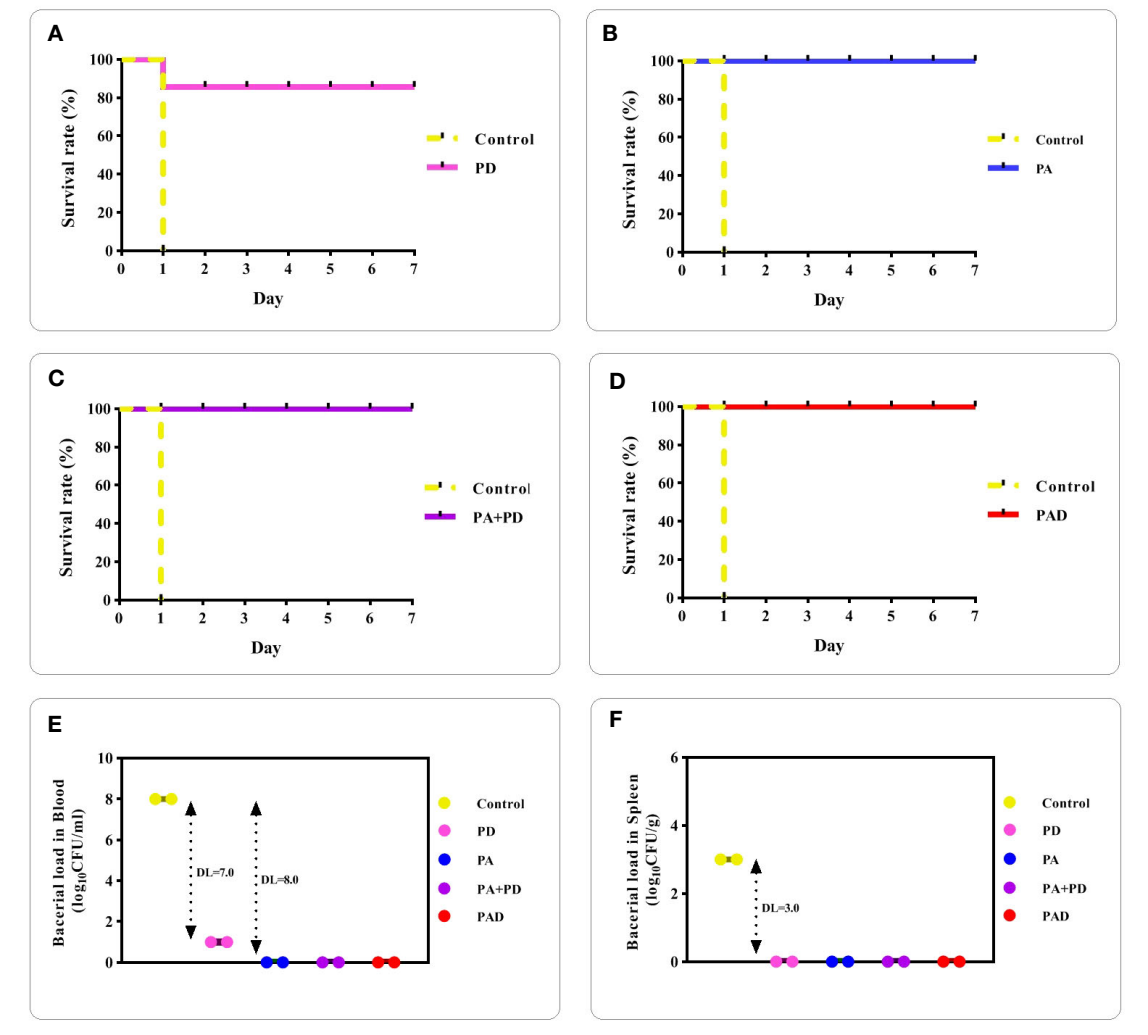
Assessment of mice survival rates and bacterial loads after pneumococcal challenge. **(A-D)** mice in the control group died one day after the challenge. The survival rate of mice up to 7 days after challenge in the PD group was 80% and in the PA, PA+PD or PAD groups was 100% (n = 3 per group). **(E, F)** Bacterial loads present in blood and spleen tissue of control and immunized mice (n = 2) were counted one day after the challenge. DLs in the blood were 7.0 Log10 (CFU/ml) for mice immunized with PD and 8.0 Log10 (CFU/ml) for the other three groups. DLs in the spleen of mice immunized with PD, PA, PA+PD and PAD were 3.0 Log10 (CFU/g).

To assess whether the recombinant proteins are capable of inducing protective immunity and decreasing bacterial burden in blood and spleen, an intraperitoneally challenge with a lethal dose of the ATCC 6303 bacteria was conducted in the mice groups after the final immunization. The survival of mice was monitored up to seven days after challenge. The mice of the control group died one day after the challenge. In the PD group, one mouse died, while all mice survived in the PA, PA+PD, and PAD groups, as shown in **Figures 11A-D**, respectively.

One day after the challenge, the number of bacteria in the blood and spleen of the mice were counted and a significant decrease of bacterial loads (DLs; log10 CFU/ml or g) was seen in the test groups compared to the control group. The bacterial loads in the blood of control mice were about 10^8^ CFU/ml, while there were 10^1^ CFU/ml of bacteria in the blood of the PD group (DL=7.0; **Figure 11E**) and no bacteria in the blood of PA, PA+PD and PAD groups (DL=8.0; **Figure 11E**). The results show that PA, PA+D and PAD groups were more effective in reducing the blood bacterial loads. The bacterial loads in the spleen of control mice were about 10^3^ CFU/g, while there were no bacteria in the spleen of the mice groups immunized with PD, PA, PA+PD and PAD (DL=3.0; **Figure 11F**).

## 4 Discussion

The commercial polysaccharide-based pneumococcal vaccines are expensive and serotype-dependent, and their immunogenicity is limited to the serotypes included in the vaccines (Norolahi et al., 2020; Bahadori et al., 2022). Therefore, pneumococcal protein vaccines providing serotype-independent immunity at low cost have been considered as interesting alternatives to existing vaccines (Converso et al., 2020; Masomian et al., 2020). The use of several protein antigens in a single vaccine, targeting multiple major bacterial virulence factors, can be a very attractive strategy to prevent infection (Lu et al., 2015). So far, different studies have demonstrated that fusion proteins containing several antigens are more efficient compared with combination formulations and could facilitate the production/purification process and product quality control (Nguyen et al., 2011; Goulart et al., 2013).

Recent improvements in immunoinformatics tools could aid in identifying possible B and T cell epitopes in protein candidates and speed up the process of developing epitope-based vaccines (Oli et al., 2020). In recent years, many research groups have developed innovative vaccine candidates employing immunoinformatics against various types of pathogens including viruses, bacteria, and parasites (such as SARS-CoV-2 (Marriam et al., 2023), *Mycobacterium tuberculosis* (Cheng et al., 2022), and *Leishmania donovani* (Saha et al., 2022)). The peptide-based vaccines are attractive alternatives to conventional vaccines since they have a lower production cost, save time, do not contain the whole pathogen, and are safer and more specific (Naz et al., 2020).

The PspA and PhtD proteins, among the pneumococcal virulence proteins, are highly immunogenic which are expressed on all serotypes and have shown very promising results in clinical studies as vaccine candidates (Converso et al., 2020; Masomian et al., 2020). The fact that PspAs are diverse in different clinical isolates may restrict the broad coverage of PspA-derived vaccines (Goulart et al., 2011). It has been found that the levels of cross-reactivity among PspAs depends on sequence similarity which is high within a PspA family and low among different families. In addition, some studies have shown that the levels of cross-reactivity and protection depends on the PspA clade (Darrieux et al., 2008; Goulart et al., 2011; Akbari et al., 2019). Recently, Akbari and colleagues have produced a PspA-derived fusion vaccine that contains fragments of the B region of PspA from the five most common clades (PspAB1-5). The vaccine would appear to have a significant protective effect against various pneumococcal strains, suggesting that using fusion proteins harboring B regions from clades 1-5 may be an effective vaccination strategy. Also, it has been proposed that the inclusion of the A and C regions of PspA protein in the vaccine construct could help elicit broadly cross-reactive antibodies (Akbari et al., 2019). The C-terminal fragment of PhtD protein is placed on the bacterial surface and Plumptre et al. showed that recombinant truncated derivatives of this fragment are highly immunogenic and capable of inducing very high titers of antibodies in comparison with the full-length PhtD protein (Plumptre et al., 2013a; Plumptre et al., 2013b).

According to our knowledge, the proteins PspA and PhtD have not been studied in a fusion form until today, and this study is the first report evaluating the fusion form of these two proteins as a vaccine candidate against *Streptococcus pneumoniae*. In the present study, the computational approaches were applied for designing potential peptide-based vaccines including the immunodominant regions of PspA (from different families) and PhtD proteins. We assessed the A, B and C regions of the PspA protein to predict the B and T cell epitopes. B regions from clades 1 to 5 were analyzed to provide wider protective coverage and decrease the possibility of PspA variants escaping host immune responses. In addition, the C-terminal of PhtD was analyzed as a promising immunogenic region for predicting B and Th cell epitopes. None of the selected epitope-rich regions of these proteins have been reported before, and this study presents for the first time novel vaccine candidates consisting of immunodominant regions of PspA and PhtD-C (a fusion construct of the two proteins and two individual constructs). Finally, the immunogenicity and protective effects of the constructs were compared individually, in combination and fusion form in mouse model.

On the basis of the immunoinformatics data of the study, the high-scoring and overlapping B and Th cell epitopes shared on multiple servers were taken into account to select the final peptides for PspA (region A, region B for two families 1 and 2, and region C) and PhtD-C (**Table 1**). An epitope-rich peptide of 64 amino acids was selected for the A region of PspA protein. After analyzing the protein sequence of the B region in different PspAs, it was found that clades 1 and 4 have the most overlap with other clades, and hence only consensus epitope sequences of region B corresponding to clades 1 and 4 were considered in the final construct: Cons-Clade 1 + 2 (29 residues covering clades 1 and 2), Cons-Clade 1 (26 amino acids covering clade 1), Cons-Clade 3 + 4 (32 residues covering clades 3 and 4) and Cons-Clade 4 + 5 (28 residues covering clades 4 and 5). Thus, it can be said that all 5 clades can be targeted by selecting these consensus sequences. To select the epitope in the C region of PspA, the 15-amino acid peptide PAPAKPEQPAPAPK was chosen, which had the highest overlap in the epitopes experimentally identified in recent studies. Approximately 46% of pneumococcal strains were found to express a copy of the repeat PKPEQP capable of eliciting protective Abs in animal models (Daniels et al., 2010), so this important motif was taken into account in this study. The epitope-rich regions of PhtD-C that were selected in this study included two peptides from amino acids 648 to 700 and amino acids 782 to 826 (53 and 45 residues in length, respectively).

In the next step of this research, the final selected peptides were connected to each other by glycine-rich flexible linkers (GGGS and GGSSGG) capable of improving solubility and enabling the neighboring domains to act freely (Kavoosi et al., 2007; Kar et al., 2020). A histidine tag was added by a glycine to the C-terminal of the developed constructs to aid protein purification. Finally, two individual protein constructs (PA: composed of PspA epitopes and PD: composed of PhtD-C epitopes) and one fusion construct (PAD: composed of PspA and PhtD-C epitopes) were designed with the aim of comparing them in the form of individual, combination and fusion proteins (**Figure 3**).

The physicochemical and immunological characteristics of the designed constructs were evaluated using different web servers (**Table 2**). The molecular weights of constructs were less than 110 kDa, showing them acceptable for vaccine development because proteins with MWs below 110 kDa are easier to purify (Sanami et al., 2020). The theoretical pI of the constructs indicated that the constructs are acidic in nature which may be useful for protein purification (Zhao et al., 2019). The instability index of the PAD or PD was less than 40, thus they were classified as stable constructs. While the instability index of the PA was above 40, so the construct stability should be confirmed in experimental tests. The predicted aliphatic indexes suggested that the constructs were thermostable (Ikai, 1980). The GRAVY indexes of the constructs were negative values which showed the hydrophilic nature of proteins and their strong interaction with water molecules and therefore their high solubility (Kyte and Doolittle, 1982). Further predictions revealed that the designed vaccine candidates have a high solubility percentage upon overexpressed in *E. coli*, as well as they are antigenic, non-allergenic and non-toxic.

The primary 3D structures of the vaccine candidates were modeled using Robetta Server and then refined by GalaxyRefine software resulting in higher quality 3D models. The quality assessment of the initial and refined models was conducted using Ramachandran diagrams, ProSA Z-scores and ERRAT scores. The obtained results showed that the refined structures possessed better quality than the initial 3D structures (**Table 3**). The prediction of B cell structural epitopes in the final constructs was done using the ElliPro server after confirming the 3D structures. The server predicted five and three potential non-linear B-cell epitopes in the PAD/PA and PD constructs, respectively (**Supplementary Table S20**). The results confirm that the vaccine candidates are able to induce the humoral immune response, which is necessary for protection against pneumococci. The proposed immunogenic regions should interact effectively with the HLA molecules for inducing efficiently immune responses. The potential interaction between the selected peptides and HLA-DRB1_01:01 was evaluated by molecular docking analysis. The results confirmed that the peptides possessed affinity for the HLA receptor (**Supplementary Table S21**). The elicitation of memory B and T cell responses is considered one of the criteria for effective vaccine candidates (Bahadori et al., 2022). The immune simulation results showed that memory cells were increased with each dose of vaccines, showing the suitability of the constructs. Moreover, it was found that the IFN-γ production was increased following the repeated the injections. The results showed that the constructs have the potential to induce humoral and cellular responses providing a basis for immunity against pneumococcal infections.

In the next step, to ensure high-level expression in *E. coli* K12, the reverse translation and codon optimization of the designed constructs were conducted using the Jcat server. For the optimized sequence of PAD, PA or PD, the GC content and the CAI value were favorable for high protein expression in the bacteria. Finally, for the purpose of *in silico* cloning, the gene sequences of vaccine candidates were successfully inserted into the pET28a(+) expression vectors. Overall, the *in silico* data showed that the vaccine candidates may be highly effective against pneumococcal infections, but in order to confirm these *in silico* results, further experimental studies were performed *in vitro* and *in vivo*.

The experimental results of expression and purification of PAD, PA and PD recombinant proteins showed that all three proteins are stable. The computational results were confirmed by expressing PAD, PA and PD recombinant proteins in *E. coli* BL21 after induction by IPTG. The computational prediction of the solubility of PAD, PA and PD proteins was confirmed by their *in vitro* purification under native conditions on Ni-NTA column. In the SDS-PAGE technique, the presence of bands corresponding to PAD, PA and PD recombinant proteins demonstrated acceptable expression and codon optimization. The band near 11, 35, or 48 kDa of the protein ladder corresponds to the PD, PA, or PAD construct, respectively. The predicted MW of PA and PAD are lower than the apparent MW on SDS-PAGE gel, indicating the slow movement of these proteins. In a study by Shirai et al., it was shown that the differences between estimated and apparent MWs could be due to the GRAVY score (Shirai et al., 2008). Due to the preferential binding of SDS to hydrophobic parts of the proteins, those that are more hydrophilic and have a lower GRAVY tend to bind less SDS, resulting in lower electrophoretic mobility and appearing to have a higher MW. This research group also demonstrated that the isoelectric point of a protein could influence the electrophoretic mobility (Shirai et al., 2008). This confirms what Guan and colleagues hypothesize, namely that a protein with many charges is likely to result in repulsion of the SDS charge and subsequently abnormal mobility on the gel (Guan et al., 2015). Moreover, the proline content in a protein also should be taken into account as another cause of greater apparent MW (Scheller et al., 2021). Since the ring structure of the proline molecule inhibits rotation of the C-N bond, the presence of proline in a protein can cause a kink in the secondary structure, leading to the polypeptide being unable to be fully stretched following reduction and SDS-binding.

In the next step, to check the immunogenic and protective effect of vaccine candidates, the purified recombinant proteins along with Alum adjuvant were injected into female BALB/c mice. The Alum adjuvant mainly induces Th2 responses, leading to increased production of antigen-specific antibodies (Leroux-Roels, 2010; Younis et al., 2019). Since pneumococcus is an extracellular pathogen and a strong Th2 response could be effective against the infection (Mizrachi-Nebenzahl et al., 2003), Alum was chosen as an adjuvant in this study. The BALB/c mice produce a stronger humoral response than other inbred strains, and it has also been shown that female BALB/c mice can induce regulatory T cells better than male mice (Elderman et al., 2018). Immunization was done subcutaneously in the mice groups PD, PA, PA+PD, PAD and Al (adjuvant control) on days 0, 14, 28 and 42. Drug injection is a preferred way for drug delivery to achieve the desired effect quickly and directly. Among the various methods of drug injection, subcutaneous injection is the one that is applied to the fat layer of the subcutaneous tissue just under the skin. Because the subcutaneous tissue possess few blood vessels, the injected drug is released very slowly with a constant rate of absorption. Therefore, it is very effective in the administration of drugs such as vaccines, which require sustained delivery at low doses (Kim et al., 2017). Two weeks after each immunization, mouse sera were collected and analyzed for active antibody production against pneumococci and cytokine production.

In confirmation of the computational predictions, the experimental results revealed that the PAD, PA and PD constructs are non-allergenic and non-toxic, so that no increased body temperature, decreased weight, allergic reactions, hypersensitivity or restlessness was observed in the animal models following the injection of the proteins. In addition, the experimental data also verified the computational analysis of the antigenicity of the recombinant proteins. The PAD, PA and PD proteins were able to increase the levels of IgG antibodies in the test mice groups compared to the control group, at different injection times (**Figure 9**). The anti-PA IgG responses showed an upward trend after the first injection, and this increase continued after the 2nd injection, but the responses remained approximately in the same range two weeks after the 3rd or 4th injections. On the 14th day, a significant difference in anti-PA IgG responses was observed among the test groups, with the superiority of the PAD group. The analysis of anti-PD IgG levels showed that the responses were increased after the 2nd injection and this increase continued after the subsequent injections. On the 28th day, a significant difference was observed among the test groups in terms of anti-PD IgG response, with the superiority of the PAD group. The anti-PA IgG1/IgG2a serum ratio in PA, PA+PD and PAD groups is close to one, indicating the balance between Th1 and Th2 responses. The anti-PD IgG1/IgG2a serum ratio in the PD, PA+PD and PAD groups is greater than one, showing that the Th2 response is dominant (**Supplementary Table S23**). A possible reason for this Th2 polarization could be the use of Alum adjuvant, which promotes Th2 immune response effective against extracellular pathogens such as pneumococcus. However, this polarization might be associated with the nature of antigens or the immunological pathway. This result is consistent with recent literature which reported that the PspA or PhtD antigens could stimulate dominant Th2 responses (Kothari et al., 2015; Malekan et al., 2019; Afshari et al., 2023b).

In the experimental phase, the levels of cytokines IFN-γ, IL-4 and IL-17A in the immunized mice groups was investigated (**Figure 10**) and the results of computational immune simulation were confirmed. The findings showed that the designed vaccine candidates were able to induce a combination of Th1, Th2 and Th17 immune responses. Cytokine IFN-γ showed a significant difference in the immunized groups of PA, PD, PA+PD and PAD compared to the control group. The highest level of IFN-γ was induced in the PA group, although it was not significantly different from the PAD group. These results show that Th1 cell responses are significantly activated by subcutaneous immunization with PA, PD (individually as well as in combination) and PAD proteins. Similarly, IL-4 and IL-17A cytokines were significantly increased in all immunized groups compared to the control group. Among the immunized groups, a significant difference was observed between the PA+PD and PAD groups, with the superiority of the fusion protein group in terms of the production of IL-4 and IL-17 cytokines. These results indicate that Th2 and Th17 responses are activated through subcutaneous immunization with PA, PD (individually and in combination) and PAD proteins, and meanwhile the PAD protein fusion activates these responses more strongly.

Serum bactericidal assay was used to evaluate *in vitro* the potential protective effects of vaccine candidates against pneumococcal strain. In this test, the complement-mediated killing activity of anti-recombinant proteins antibodies was analyzed after the last injection. The activity of Abs in destroying almost 50% of bacteria compared to the control was considered at a dilution of 1:16 for the PA, PA+PD or PAD group, and at a dilution of 1:4 for the PD group (**Figure 10D**). The results show that these antibodies could be able to clear the bacteria with the help of complement.

In this study, the protective effect of epitope-based vaccine candidates against *Streptococcus pneumoniae* strain ATCC 6303 was investigated after the final injection. The results of the challenge showed that the PA construct is more effective than the PD construct in the survival of mice. The survival rate of mice in the PA, PA+PD and PAD groups was 100% and complete clearing of bacteria in blood/spleen was observed one day after the challenge. While in the PD group, the survival rate was 80%, and the bacterial loads in the blood was reduced to 10^1^ CFU (DL=7.0), while complete clearing of bacteria was observed in the spleen.

As seen in the present study, in comparison between individual constructs, although the PD construct is a suitable vaccine target, it is not as effective as the PA construct alone as an immunogen and is not sufficient to provide complete protection against pneumococcal infections. The different immunoreactivity results of the two individual formulations PD and PA may be attributed to several factors, e.g. protein molecular weight, chemical/physical properties, composition and degradability (Schellekens, 2005). However, the PD construct played an important role by stimulating the production of cytokines. It has been found that IL-17 plays a key role in protecting against pneumococcus by decreasing bacterial density and colonization in the nasopharynx (Lu et al., 2015). In general, based on the findings, it can be concluded that the most effective formulation against pneumococcus is the one containing both constructs in order to produce broader immune responses. In the comparison between the combination of constructs and the fusion construct, it can be said that both formulations were able to elicit relatively similar protective effects, although the fusion peptide enhanced Th2 and Th17 responses more favorably. This shows that in the fusion construct, the addition of peptides to each other has not changed their structure, and the immunogenic epitopes have been made available and the antigenic properties of the peptides have been preserved. Another advantage of the fusion construct was that it could simplify the process of production/purification of antigens and facilitate product quality control. Overall, the existing data confirm that in the vaccine development, a protein fusion could be more effective than a protein alone or a combination of proteins.

These results are consistent with those of Nguyena et al., who found that immunization of mice with FlaB–PspA fusion protein, compared to immunization with PspA alone or a mixture of PspA and FlaB, is able to induce more effective mucosal immunity against pneumococcal infection (Nguyen et al., 2011). The FlaB–PspA fusion protein had longer half-life, potentiated IgG and IgA antibody responses, and provided the best protection against challenge with *S. pneumoniae*. FlaB–PspA fusion protein stimulated IL-4 and IFN-γ production and significantly enhanced IL-4 production and Th2 responses, being consistent with the IgG subtype responses. Also, our results agree with the results of Lu et al. that showed PsaA–PspA fusion protein could stimulate production of high-titered antibodies against *S. pneumoniae* strains comparable to each antigen alone (Lu et al., 2015). The PsaA–PspA significantly reduced bacterial levels in blood/organs and provided a high survival rate of up to 100% for some strains after intraperitoneal challenge. The PsaA–PspA fusion protein could induce a much higher level of IL-17A than the other formulations. Furthermore, our results are consistent with those of Converso et al., who reported rPspA-PotD fusion protein could induce an increased antibody production when compared with the individual proteins (Converso et al., 2017a). The rPspA-PotD fusion group presented the highest secretion of IL-17 which was associated with a reduced pneumococcal colonization. The rPspA-PotD was able to enhance phagocytosis, reduce bacterial loads in the nasopharynx and elicit wide protection against infection.

Moreover, the results of this study reveal the success of computational tools in designing epitope-based vaccine candidates, which are consistent with various studies proving the reliability of immunoinformatics approaches (Ahmadi et al., 2019; Hasanzadeh et al., 2020; Cheng et al., 2023). In this context, Zhang et al. designed and evaluated a multi-epitope subunit vaccine against group B Streptococcus (GBS) infection, and experimental results consistent with *in silico* data showed that the proposed construct is capable of inducing strong immune responses and is an ideal vaccine candidate against GBS (Zhang et al., 2022).

## 5 Conclusion

In the present study, the epitope-based vaccine candidate in the form of a fusion of PspA and PhtD epitopes was compared with the individual and combination formulations. The obtained findings confirmed that the fusion formulation was relatively more efficient and effective than the other formulations, although this issue requires further studies. The fusion construct was able to produce a high specific IgG titer and was able to induce a combination of Th1, Th2 and Th17 immune responses. Also, antisera raised against the fusion construct had a favorable effect on the mediation of complement-dependent bacterial killing and provided 100% survival in immunized mice after bacterial challenge. The experimental validation results of the designed vaccine candidate also confirmed the immunoinformatics studies. Overall, it can be concluded that the fusion construct can be considered as a promising vaccine candidate against pneumococcal infection with significant prospects. The next plans for optimizing the vaccine candidate will be to change the adjuvant, concentration of immunogen, and injection route/timing, as well as the assessment of mucosal immunity and the use of different pneumococcus strains expressing other clades of PspA for investigating the cross-reactive antibodies responses. Further studies in the future can focus on other epitope-based fusion constructs consisting of promising immunogenic antigen candidates for achieving more robust immune response and protection.

## Data availability statement

The datasets presented in this study can be found in online repositories. The names of the repository/repositories and accession number(s) can be found in the article/**Supplementary Material**.

## Ethics statement

The animal study was approved by Animal Ethical Committee of Semnan University of Medical Sciences, Semnan, Iran (Approved Number: IR.SEMUMS.REC.1399.128). The study was conducted in accordance with the local legislation and institutional requirements.

## Author contributions

MS: Conceptualization, Data curation, Formal analysis, Investigation, Methodology, Validation, Writing - review & editing, Writing original draft. ZB: Conceptualization, Data curation, Formal analysis, Investigation, Methodology, Validation, Writing-original draft. SB: Methodology. EA: Methodology. HM: Validation. SM: Project administration, Supervision. AS: Project administration, Supervision.
