# Supplementary material for: A new candidate epitope-based vaccine against PspA PhtD of Streptococcus pneumoniae: a computational experimental approach
Source: Front Cell Infect Microbiol. 2023 Nov 15;13:1271143. doi: 10.3389/fcimb.2023.1271143 (PMC10684780; doi:10.3389/fcimb.2023.1271143)
Supplement: Supplementary file 1 [file DataSheet_1.docx]

***Supplementary Material***

# 1 Supplementary Tables and Figures

## 1.1 Supplementary Tables

### Supplementary Table S1. Accession numbers of PspA protein sequences from different pneumococcal strains in the NCBI database.

| **Strain name** | **Genbank Accession number** | **Strain name** | **Genbank Accession number** | **Strain name** | **Genbank Accession number** |
| --- | --- | --- | --- | --- | --- |
| AC122* | AAF27714.1 | GA05245 | EHZ06901.1 | NP141 | EHZ99220.1 |
| AC94* | AAF27698.1 | GA07228 | EHE68717.1 | OXC141 | WP_001035317.1 |
| ATCC6303* | AAF27715.1 | GA07914 | EHZ13312.1 | P1031 | WP_001035309.1 |
| BG11703* | AAF27716.1 | GA08780 | EHE69704.1 | PCS70012 | ELU63511.1 |
| BG6380* | AAF27717.1 | GA11304 | WP_024478546.1 | PNI0153 | ELU78415.1 |
| BG6692* | AAF27704.1 | GA13224 | EHZ20138.1 | SP-BS293 | EFL70049.1 |
| BG7516* | AAF27718.1 | GA13430 | EHZ18233.1 | SP14-BS292 | EFL68254.1 |
| BG7817* | AAF27719.1 | GA13637 | EHD96455.1 | SP14-BS69 | EDK67229.1 |
| BG8090* | AAF27713.1 | GA13723 | WP_001035359.1 | SP18-BS74 | EDK69190.1 |
| BG8743* | AAF27699.1 | GA13856 | EHD94046.1 | SP19-BS75 | EDK71559.1 |
| BG8838* | AAF27703.1 | GA14373 | EHZ25632.1 | SP23-BS72 | EDK82340.1 |
| BG9163* | AAF27711.1 | GA16242 | EHE02641.1 | SP3-BS71 | EDK74780.1 |
| BG9739* | AAF27700.1 | GA16531 | EHD52508.1 | SP6-BS73 | EDK77396.1 |
| DBL1* | AAF27702.1 | GA17301 | EJG94099.1 | SP9-BS68 | EDK79448.1 |
| DBL5* | AAF27706.1 | GA17328 | EHE10903.1 | SPAR55 | EJG83087.1 |
| DBL6A* | AAF27701.1 | GA17484 | EJH13744.1 | SPAR95 | EJG83549.1 |
| E134* | AAF27707.1 | GA17971 | EHE11974.1 | SPN034183 | WP_015545692.1 |
| EF10197* | AAF27708.1 | GA18068 | EHZ32568.1 | SPN072838 | WP_001035339.1 |
| EF3296* | AAF27712.1 | GA18523 | EHD73296.1 | SPNA45 | CCM09196.1 |
| EF5868* | AAC62252.1 | GA19101 | EHZ39315.1 | Hungary19A-6 | ACA36951.1 |
| EF6796* | AAF27709.1 | GA19690 | EHE73248.1 | INV104 | WP_001035356.1 |
| L81905* | AAF27705.1 | GA19923 | EHZ36433.1 | ND6012 | WP_001035297.1 |
| R6* | NP_357715.1 | GA40028 | WP_061815997.1 | Netherlands15B-37 | EHE54378.1 |
| RX1* | AAA27018.1 | GA40563 | EHZ47469.1 | NP112 | WP_000604177.1 |
| A66.1* | CRI60845.1 | GA41410 | EHD59901.1 | GA02270 | EHY99083.1 |
| WU2* | AAF27710.1 | GA43380 | EHE30006.1 | GA02506 | EIA04396.1 |
| 70585 | ACO18024.1 | GA44128 | EHZ53365.1 | GA02714 | EHZ00510.1 |
| 2061376 | EJG59200.1 | GA47210 | EHZ60794.1 | GA04175 | EHZ06172.1 |
| 2070109 | EJG44022.1 | GA47373 | EHE35485.1 | GA04216 | EJG95376.1 |
| 2070531 | EJG51086.1 | GA47439 | EHE40916.1 | GA60132 | EJH20917.1 |
| 2071004 | EJG64371.1 | GA47502 | WP_061745222.1 | GA62331 | EJH17394.1 |
| 2080913 | EJG70660.1 | GA47522 | EHZ67469.1 | gamPNI0373 | AFS42270.1 |
| 3063-00 | EHE63738.1 | GA47562 | EJH25918.1 | CGSP14 | WP_001035312.1 |
| 459-5 | EIC60454.1 | GA47597 | EHZ68388.1 | EU-NP04 | EHZ95578.1 |
| 670-6B | WP_001035308.1 | GA47628 | EHZ67911.1 | G54 | ACF54852.1 |
| 6963-05 | EHD71913.1 | GA47751 | EHE55694.1 | CDC1873-00 | EDT50938.1 |
| 7533-05 | EHZ85653.1 | GA47794 | EHZ74492.1 | CDC3059-06 | EDT96646.1 |
| AP200 | WP_001035331.1 | GA47976 | EHE46608.1 | CDC1087-00 | EDT90528.1 |
| BS397 | EFL77253.1 | GA49138 | EHD47872.1 | GA56348 | WP_061633400.1 |
| CCRI_1974 | WP_088850314.1 | GA49447 | EHD64813.1 |  |  |
| CDC0288-04 | EDT93968.1 | GA52612 | EJG87017.1 |  |  |
| *Reference strain | | | | | |

### Supplementary Table S2. PspA sequence from reference strain AC122. The sequence of region A is highlighted in green, and regions B and C are shown in red and blue, respectively.

| **>AC122** |
| --- |
| EEAPVASQSKAEKDYDAAVKKSEAAKKHYEEVKKKAEDAQKKYDEGQKKTVEKAKREKEASEKIAEATKEVQQAYLAYQQASNESQRKEADKKIKEATQRKDEAEAAFATIRTTIVVPEPSELAETKKKAEEAKAEEKVAKRKYDYATLKVALAKKEVEAKELEIEKLQYEISTLEQEVATAQHQVDNLKKLLAGADPDDGTEVIEAKLNKGEAELNAKQAELAKKQTELEKLLDSLDPEGKTQDELDKEAEEAELDKKADELQNKVADLEKEISNLEILLGGADSEDDTAALQNKLATKKAELEKTQKELDAALNELGPDGDEEETPAPAPQPEQPAPAPKPEQPTPAPKPEQPTPAPKPEQPAPAPKPEQPAPAPKPEQPAPAPKPEQPTPGPKIE |

### Supplementary Table S3. Pneumococcal strains belonging to families 1 and 2. Strains with non-repetitive CDR were used for further evaluations.

| **Family 1** | **Clade 1** | BG6692, BG8743, GA60132, BG8838, GA60080, GA47502, DBL6A, BG9739, GA04175, INV104, 70585, DBL1, GA13723, AC94 |
| --- | --- | --- |
|  | **Clade 2** | ND6012, E134, BG9163, DBL5, EF10197, R6, GA62331, WU2 |
| **Family 2** | **Clade 3** | GA41410, GA43380, AC122, GA18523, GA13430, GA52612, GA47794, BG8090, GA02506, SPN034183, 459-5, GA14373, SP3-BS71, OXC141 |
|  | **Clade 4** | EF5668, SP19-BS75, GA40563, BG7561, BG11703 |
|  | **Clade 5** | SPAR95, GA47373, Hungary19A-6, ATCC6303 |

### Supplementary Table S4. Protein sequence of PhtD. The C- terminal of PhtD (amino acids 383 to 853) is shown in color and underlined.

| **Protein name** | **Strain** | **GenBank ID** | **Sequence** |
| --- | --- | --- | --- |
| PhtD | R6 | AAK99711.1 | *mkinkkylagsvavlalsv*CSYELGRHQAGQVKKESNRVSYIDGDQAGQKAENLTPDEVSKREGINAEQIVIKITDQGYVTSHGDHYHYYNGKVPYDAIISEELLMKDPNYQLKDSDIVNEIKGGYVIKVDGKYYVYLKDAAHADNIRTKEEIKRQKQERSHNHNSRADNAVAAARAQGRYTTDDGYIFNASDIIEDTGDAYIVPHGDHYHYIPKSDLSASELAAAQAYWNGKQGSRPSSSSSHNANPAQPRLSENHNLTVTPTYHQNQGENISSLLRELYAKPLSERHVESDGLIFDPAQITSRTANGVAVPHGDHYHFIPYSQLSPLEEKLARIIPLRYRSNHWVPDSRPEQPSPQSTPEPSPSPQPAPNPQPAPSNPIDEKLVKEAVRKVGDGYVFEENGVPRYIPAKDLSAETAAGIDSKLAKQESLSHKLGAKKTDLPSSDREFYNKAYDLLARIHQDLLDNKGRQVDFEALDNLLERLKDVSSDKVKLVDDILAFLAPIRHPERLGKPNAQITYTDDEIQVAKLAGKYTTEDGYIFDPRDITSDEGDAYVTPHMTHSHWIKKDSLSEAERAAAQAYAKEKGLTPPSTDHQDSGNTEAKGAEAIYNRVKAAKKVPLDRMPYNLQYTVEVKNGSLIIPHYDHYHNIKFEWFDEGLYEAPKGYSLEDLLATVKYYVEHPNERPHSDNGFGNASDHVQRNKNGQADTNQTEKPNEEKPQTEKPEEDKEHDEVSEPTHPESDEKENHVGLNPSADNLYKPSTDTEETEEEAEDTTDEAEIPQVEHSVINAKIAEAEALLEKVTDSSIRQNAVETLTGLKSSLLLGTKDNNTISAEVDSLLALLKESQPTPIQ |

### Supplementary Table S5. Experimentally determined B and T cell epitopes in the A region of PspAs.

| Experimentally approved epitopes of PspA-A | Assay Types | Reference |
| --- | --- | --- |
| VRAEEAPVASQSKAE | B Cell Assays | Vadesilho; 2014 |
| ANQSKAEKDYD |  |  |
| ASQSKAEKDYDAAMK |  |  |
| DYDAAVKKSEAAKKD |  |  |
| ASQSKAEKDYD |  |  |
| ASQSKAEKDYDAAVK |  |  |
| KAEKDYDAAVK |  |  |
| DYDAAVKKYEA |  |  |
| AVKKYEAAKKE |  |  |
| KKAEDAQKKYD |  |  |
| KKAEDAQKKYDEDQK |  |  |
| SEAAKKHYEEV |  |  |
| KKEYEDGKAAQKKYE |  |  |
| EDGKAAQKKYE |  |  |
| EVQNAYVKYQRVQR |  |  |
| KYQQELV |  |  |
| ASQPTVVRAEESPVA | T Cell Assays | Singh; 2010 |
| SPVASQSKAEKDYDA |  |  |
| DYDAAKKDAKNAKKA |  |  |
| AKKAVEDAQKALDDA |  |  |
| LDDAKAAQKKYDEDQ |  |  |
| DEDQKKTEEKAALEK |  |  |
| ALEKAASEEMDKAVA |  |  |
| KAVAAVQQAYLAYQQ |  |  |
| AYQQATDKAAKDAAD |  |  |

### Supplementary Table S6. Experimentally determined B and T cell epitopes in the B region of PspAs.

| Experimentally approved epitopes of PspA-B | Molecule | Assay Types | Reference |
| --- | --- | --- | --- |
| ESDSEDYV | PspA1 | B Cell Assays | Vadesilho; 2014 |
| ESDSEDYVK | PspA1 |  |  |
| SDGEQAGQYLAAAEE | PspA1 |  |  |
| DSEDDTAA | PspA3 |  |  |
| NNVEDYIK | PspA4 |  |  |
| TNNVEDYIKEGLEEA | PspA4 |  |  |
| NNVEDYIKEG | PspA4 |  |  |
| NNVEDYVKEG | PspA5 |  |  |
| QELKEIDESESEDYA | PspA2 | T Cell Assays | Singh; 2010 |
| EDYAKEGFRAPLQSK | PspA2 |  |  |
| LQSKLDAKKAKLSKL | PspA2 |  |  |

### Supplementary Table S7. Experimentally determined epitopes in the C region of PspAs.

| Experimentally approved epitopes of PspA-C | Ref. |
| --- | --- |
| EKSADQQAEEDYARRSEEEYNRLTQQQ | Daniels 2010 |
| PKPEQ |  |
| PAPAPKPEQPAPAPK | Vadesilho 2014 |
| APKPE |  |
| APKPEQPA |  |
| EEDYARRSEEEYNRL |  |
| PAPKPEQPAEQPKPAPAPQPAPAPKPEKT | Tamborrini 2015 |
| QQAEEDYARRSEEEYNRLPQQQPPKAEKP |  |
| PKPEQPAPAPK |  |
| PKPEQPAPAPKPEQPAKPEKP |  |
| PEQPAKPEKP |  |

### Supplementary Table S8. Predicted B-cell epitopes in the A region of PspA from strain AC122.

| LBTope | | | | | | | | | | |
| --- | --- | --- | --- | --- | --- | --- | --- | --- | --- | --- |
| Sequence | **% Probabilty of correct prediction** | **Sequence** | | **% Probabilty of correct prediction** | **Sequence** | **% Probabilty of correct prediction** | **Sequence** | | | **% Probabilty of correct prediction** |
| KKHYEEVKKKAEDAQ | 66.99 | HYEEVKKKAEDAQKK | | 66.41 | EEVKKKAEDAQKKYD | 63.47 | VKKKAEDAQKKYDEG | | | 61.68 |
| KHYEEVKKKAEDAQK | 80.32 | YEEVKKKAEDAQKKY | | 70.21 | EVKKKAEDAQKKYDE | 63.41 | KKKAEDAQKKYDEGQ | | | 60.38 |
| ABCpred | | | | | | | | | | |
| Sequence | **Score** | **Sequence** | | **Score** | **Sequence** | **Score** | **Sequence** | | | **Score** |
| KEVQQAYLAYQQASNE | 0.87 | KKAEDAQKKYDEGQKK | | 0.86 | LAYQQASNESQRKEAD | 0.77 | HYEEVKKKAEDAQKKY | | | 0.68 |
| AKREKEASEKIAEATK | 0.86 | SEAAKKHYEEVKKKAE | | 0.85 | SEKIAEATKEVQQAYL | 0.74 | ESQRKEADKKIKEATQ | | | 0.64 |
| YDEGQKKTVEKAKREK | 0.86 | PVASQSKAEKDYDAAV | | 0.82 | EKDYDAAVKKSEAAKK | 0.73 |  | | |  |
| Emini surface accessibility Prediction | | | | | | | | | | |
| Sequence | | **Sequence** | | | **Sequence** | |  | | | |
| KAEKD (10-14) | | KA (35-36) | | | AKREKEA (54-60) | |  | | | |
| KH (27-28) | | AQKKYDEGQ (39-47) | | | ESQRKEA (84-90) | |  | | | |
| EV (31-32) | | KT (49-50) | | | K (93), A (97) | |  | | | |
| Ellipro (predicted linear epitopes) | | | | | | | | | | |
| Sequence | **Score** | | **Sequence** | **Score** | **Sequence** | **Score** |  | | |  |
| DYDAAVKKSEAAKKHYE | 0.754 | | VQQAYLAYQQASNESQ | 0.734 | KREKEASEKIAE | 0.708 |  | | |  |
|  |  |  |  |  |  |  | |  |  |  |
| Ellipro (predicted discontinuous epitopes) | | | | | | | | | | |
| Residues | **Score** | | **Residues** | **Score** | **Residues** | **Score** |  | | |  |
| V102, Q103, Q104, A105, Y106, L107, A108, Y109, Q110, Q111, A112, S113, N114, S116, Q117 | 0.756 | | A42, D45, Y46, D47, A48, A49, V50, K51, K52, S53, E54, A55, A56, K57, K58, Y60, E61, K64 | 0.752 | R87, E88, K89, E90, A91, S92, E93, K94, I95, A96, E97 | 0.735 |  | | |  |

### Supplementary Table S9. Prediction of MHC-II epitopes in A region of PspA from strain AC122. IEDB peptides with percentile rank <10.0 and NetMHCIIpan peptides with rank value <1.0 were considered for further analysis.

| IEDB | | | | | | | |  |
| --- | --- | --- | --- | --- | --- | --- | --- | --- |
| Allele | **Peptide** | **Percentile Rank** | **Allele** | **Peptide** | **Percentile Rank** | **Allele** | **Peptide** | **Percentile Rank** |
| H2-IEd | AKKHYEEVKKKAEDA | 4.70 | H2-IEd | SEAAKKHYEEVKKKA | 8.00 | DRB1*08:01 | EVQQAYLAYQQASNE | 3.90 |
| H2-IEd | EAAKKHYEEVKKKAE | 4.70 | DRB1*04:01 | QAYLAYQQASNESQR | 1.20 | DRB1*08:01 | KEVQQAYLAYQQASN | 3.90 |
| H2-IEd | AAKKHYEEVKKKAED | 4.80 | DRB1*04:01 | AYLAYQQASNESQRK | 1.50 | DRB1*08:01 | QAYLAYQQASNESQR | 3.90 |
| H2-IEd | KKHYEEVKKKAEDAQ | 7.40 | DRB1*04:01 | YLAYQQASNESQRKE | 1.60 | DRB1*08:01 | QQAYLAYQQASNESQ | 3.90 |
| H2-IAd | ATKEVQQAYLAYQQA | 7.65 | DRB1*04:01 | LAYQQASNESQRKEA | 1.90 | DRB1*08:01 | VQQAYLAYQQASNES | 3.90 |
| H2-IAd | TKEVQQAYLAYQQAS | 7.70 | DRB1*04:01 | QQAYLAYQQASNESQ | 3.40 | DRB1*08:01 | YLAYQQASNESQRKE | 3.90 |
| H2-IAb | KEVQQAYLAYQQASN | 7.90 | DRB1*08:01 | AYLAYQQASNESQRK | 3.90 | DRB1*04:01 | AYQQASNESQRKEAD | 4.20 |
| NetMHCIIpan | | | | | | | |  |
| Allele | **Peptide** | **Percentile Rank** | **Allele** | **Peptide** | **Percentile Rank** | **Allele** | **Peptide** | **Percentile Rank** |
| H-2-IEd | EAPVASQSKAEKDYD | 0.960 | DRB1_0401 | YLAYQQASNESQRKE | 0.13 | DRB1_0801 | SEKIAEATKEVQQAY | 1.65 |
| H-2-IEd | EKDYDAAVKKSEAAK | 0.920 | DRB1_0401 | LAYQQASNESQRKEA | 0.54 | DRB1_1101 | KAEKDYDAAVKKSEA | 1.57 |
| H-2-IEd | DYDAAVKKSEAAKKH | 0.653 | DRB1_0801 | KAEKDYDAAVKKSEA | 0.25 | DRB1_1101 | AEKDYDAAVKKSEAA | 0.72 |
| H-2-IEd | YDAAVKKSEAAKKHY | 0.873 | DRB1_0801 | AEKDYDAAVKKSEAA | 0.10 | DRB1_1101 | EKDYDAAVKKSEAAK | 0.39 |
| H-2-IEd | DAAVKKSEAAKKHYE | 0.960 | DRB1_0801 | EKDYDAAVKKSEAAK | 0.06 | DRB1_1101 | KDYDAAVKKSEAAKK | 1.22 |
| H-2-IEd | AAKKHYEEVKKKAED | 0.740 | DRB1_0801 | KDYDAAVKKSEAAKK | 0.39 | DRB1_1101 | AAKKHYEEVKKKAED | 0.50 |
| H-2-IEd | AKKHYEEVKKKAEDA | 0.880 | DRB1_0801 | EAAKKHYEEVKKKAE | 0.44 | DRB1_1101 | AKKHYEEVKKKAEDA | 0.24 |
| H-2-IEd | KKHYEEVKKKAEDAQ | 0.960 | DRB1_0801 | AAKKHYEEVKKKAED | 0.03 | DRB1_1101 | KKHYEEVKKKAEDAQ | 0.17 |
| H-2-IAd | AKREKEASEKIAEAT | 0.820 | DRB1_0801 | AKKHYEEVKKKAEDA | 0.01 | DRB1_1101 | KHYEEVKKKAEDAQK | 0.59 |
| H-2-IAd | KREKEASEKIAEATK | 0.980 | DRB1_0801 | KKHYEEVKKKAEDAQ | 0.01 | DRB1_1101 | KKTVEKAKREKEASE | 1.93 |
| H-2-IAd | REKEASEKIAEATKE | 0.967 | DRB1_0801 | KHYEEVKKKAEDAQK | 0.06 | DRB1_1101 | EASEKIAEATKEVQQ | 1.76 |
| H-2-IAd | EKEASEKIAEATKEV | 0.720 | DRB1_0801 | HYEEVKKKAEDAQKK | 1.46 | DRB1_1101 | ASEKIAEATKEVQQA | 0.65 |
| H-2-IAd | KEASEKIAEATKEVQ | 0.813 | DRB1_0801 | GQKKTVEKAKREKEA | 1.42 | DRB1_1101 | SEKIAEATKEVQQAY | 0.53 |
| H-2-IAd | EASEKIAEATKEVQQ | 0.967 | DRB1_0801 | QKKTVEKAKREKEAS | 0.76 | DRB1_1301 | EAPVASQSKAEKDYD | 1.58 |
| DRB1_0401 | QAYLAYQQASNESQR | 0.953 | DRB1_0801 | KKTVEKAKREKEASE | 0.54 | DRB1_1301 | KKTVEKAKREKEASE | 1.52 |
| DRB1_0401 | AYLAYQQASNESQRK | 0.993 | DRB1_0801 | ASEKIAEATKEVQQA | 1.76 | DRB1_1301 | SEKIAEATKEVQQAY | 1.72 |

### Supplementary Table S10. Predicted B cell epitopes in the B region of PspA clade 1 (DBL6A).

| LBTope* | | | | | | | | |
| --- | --- | --- | --- | --- | --- | --- | --- | --- |
| Sequence | **% Probabilty of correct prediction** | **Sequence** | | **% Probabilty of correct prediction** | **Sequence** | **% Probabilty of correct prediction** | **Sequence** | **% Probabilty of correct prediction** |
| KEIDESDSEDYVKEG | 64.42 | SKLEELSDKIDELDA | | 73.12 | DAEIAKLEKDVEDFK | 85.79 | QAGQYLAAAEEDLIA | 60.75 |
| EIDESDSEDYVKEGF | 60.99 | KLEELSDKIDELDAE | | 64.43 | AEIAKLEKDVEDFKN | 90.68 | AGQYLAAAEEDLIAK | 71.84 |
| IDESDSEDYVKEGFR | 62.63 | LEELSDKIDELDAEI | | 72.47 | EIAKLEKDVEDFKNS | 88.37 | GQYLAAAEEDLIAKK | 73.32 |
| DESDSEDYVKEGFRA | 69.16 | EELSDKIDELDAEIA | | 73.55 | IAKLEKDVEDFKNSD | 70.88 | QYLAAAEEDLIAKKA | 64.78 |
| ESDSEDYVKEGFRAP | 65.61 | ELSDKIDELDAEIAK | | 66.99 | AKLEKDVEDFKNSDG | 63.89 | YLAAAEEDLIAKKAE | 60.07 |
| SEDYVKEGFRAPLQS | 60.02 | LSDKIDELDAEIAKL | | 72.94 | KLEKDVEDFKNSDGE | 61.30 | LAAAEEDLIAKKAEL | 66.52 |
| EDYVKEGFRAPLQSE | 60.02 | SDKIDELDAEIAKLE | | 73.56 | LEKDVEDFKNSDGEQ | 62.15 | AAAEEDLIAKKAELE | 66.56 |
| DYVKEGFRAPLQSEL | 75.49 | DKIDELDAEIAKLEK | | 89.09 | DVEDFKNSDGEQAGQ | 73.90 | AAEEDLIAKKAELEQ | 61.32 |
| YVKEGFRAPLQSELD | 66.81 | KIDELDAEIAKLEKD | | 81.34 | EDFKNSDGEQAGQYL | 64.85 | LEQTEADLKKAVNEX | 65.55 |
| VKEGFRAPLQSELDA | 60.23 | IDELDAEIAKLEKDV | | 84.23 | DFKNSDGEQAGQYLA | 60.23 | EQTEADLKKAVNEXX | 72.01 |
| EGFRAPLQSELDAKQ | 65.46 | DELDAEIAKLEKDVE | | 84.26 | FKNSDGEQAGQYLAA | 66.00 | QTEADLKKAVNEXXX | 66.31 |
| ELDAKQAKLSKLEEL | 65.31 | ELDAEIAKLEKDVED | | 94.31 | DGEQAGQYLAAAEED | 72.47 | TEADLKKAVNEXXXX | 67.83 |
| LDAKQAKLSKLEELS | 62.05 | LDAEIAKLEKDVEDF | | 81.43 | GEQAGQYLAAAEEDL | 65.13 | EADLKKAVNEXXXXX | 60.02 |
| ABCpred | | | | | | | | |
| Sequence | **Score** | **Sequence** | | **Score** | **Sequence** | **Score** | **Sequence** | **Score** |
| LKEIDESDSEDYVKEG | 0.86 | DSEDYVKEGFRAPLQS | | 0.83 | LDAEIAKLEKDVEDFK | 0.80 | AELEQTEADLKKAVNE | 0.75 |
| SDKIDELDAEIAKLEK | 0.85 | AGQYLAAAEEDLIAKK | | 0.80 | APLQSELDAKQAKLSK | 0.78 |  |  |
| Emini surface accessibility Prediction | | | | | | | | |
| Sequence | | **Sequence** | | | **Sequence** | | **Sequence** | |
| LKEIDESDSEDYVKEG | | SELDAKQAKLSK | | | EELSDKIDELDAE | | AKLEKDVEDFKNSDGEQA | |
|  |  |  | |  |  |  |  |  |
| Ellipro (predicted linear epitopes) | | | | | | | | |
| Sequence | **Score** | | **Sequence** | **Score** | **Sequence** | **Score** |  |  |
| LKEIDESDSEDYVKEGFRAP | 0.755 | | KDVEDFKNSDGEQAGQYLA | 0.698 | ADLKKAVNE | 0.656 |  |  |
| Ellipro (predicted discontinuous epitopes) | | | | | | | | |
| Residues | **Score** | | **Residues** | **Score** | **Residues** | **Score** | **Residues** | **Score** |
| L1, K2, E3, I4, D5, E6, S7, D8, S9, E10, D11, Y12, V13, K14, E15, G16, F17, R18, A19, P20 | 0.755 | | D58, F59, K60, N61, S62, D63, G64, E65, Q66, A67, G68, Q69, Y70, A72, E76 | 0.747 | A83, E86, Q87, A90, D91, K93 | 0.643 | K54, D55, E57 | 0.616 |
| DiscoTope 2.0 | | | | | | | | |
| Sequence |  | | **Sequence** |  |  |  |  |  |
| LKEIDESDSEDYVKEGFRAPLQS | | | KNSDGEQAGQ |  |  |  |  |  |
|  | | |  |  |  |  |  |  |

### Supplementary Table S11. Predicted B cell epitopes in the B region of PspA clade 2 (R6).

| LBTope* | | | | | | | |
| --- | --- | --- | --- | --- | --- | --- | --- |
| Sequence | **% Probabilty of correct prediction** | **Sequence** | **% Probabilty of correct prediction** | **Sequence** | **% Probabilty of correct prediction** | **Sequence** | **% Probabilty of correct prediction** |
| SEDYAKEGFRAPLQS | 61.34 | APLQSKLDAKKAKLS | 69.39 | LEELSDKIDELDAEI | 72.47 | KAAEENNNVEDYFKE | 64.72 |
| EDYAKEGFRAPLQSK | 66.76 | PLQSKLDAKKAKLSK | 71.28 | EELSDKIDELDAEIA | 73.55 | AAEENNNVEDYFKEG | 63.16 |
| DYAKEGFRAPLQSKL | 72.16 | QSKLDAKKAKLSKLE | 61.90 | ELSDKIDELDAEIAK | 66.99 | FKEGLEKTIAAKKAE | 64.99 |
| YAKEGFRAPLQSKLD | 72.65 | KLDAKKAKLSKLEEL | 61.47 | LSDKIDELDAEIAKL | 72.94 | KEGLEKTIAAKKAEL | 61.79 |
| AKEGFRAPLQSKLDA | 60.75 | LDAKKAKLSKLEELS | 72.99 | SDKIDELDAEIAKLE | 73.56 | EKTEADLKKAVNEP | 72.32 |
| KEGFRAPLQSKLDAK | 60.75 | DAKKAKLSKLEELSD | 62.71 | DKIDELDAEIAKLED | 87.06 | KTEADLKKAVNEP | 71.50 |
| EGFRAPLQSKLDAKK | 73.04 | AKKAKLSKLEELSDK | 62.45 | KIDELDAEIAKLEDQ | 70.13 | TEADLKKAVNEP | 69.79 |
| GFRAPLQSKLDAKKA | 61.68 | KKAKLSKLEELSDKI | 60.51 | IDELDAEIAKLEDQL | 60.87 | EADLKKAVNEP | 63.42 |
| FRAPLQSKLDAKKAK | 70.16 | SKLEELSDKIDELDA | 73.12 | EIAKLEDQLKAAEEN | 62.43 |  |  |
| RAPLQSKLDAKKAKL | 62.13 | KLEELSDKIDELDAE | 64.43 | IAKLEDQLKAAEENN | 60.18 |  |  |
| ABCpred | | | | | | | |
| Sequence | **Score** | **Sequence** | **Score** | **Sequence** | **Score** | **Sequence** | **Score** |
| ESEDYAKEGFRAPLQS | 0.90 | ELEKTEADLKKAVNEP | 0.83 | EGLEKTIAAKKAELEK | 0.70 | APLQSKLDAKKAKLSK | 0.70 |
| SDKIDELDAEIAKLED | 0.87 | LKEIDESESEDYAKEG | 0.81 | LEDQLKAAEENNNVED | 0.70 | NNNVEDYFKEGLEKTI | 0.63 |
| Emini surface accessibility Prediction | | | | | | | |
| Sequence | | **Sequence** | | **Sequence** | |  | |
| SESEDYA | | AEENNNV | | AELEKT | |  | |
|  |  |  |  |  |  |  |  |
| Ellipro (predicted linear epitopes) | | | | | | | |
| Sequence | **Score** | **Sequence** | **Score** |  |  |  |  |
| LKEIDESESEDYAKEGFRAP | 0.741 | DQLKAAEENNNVEDYFKEG | 0.778 |  |  |  |  |
| Ellipro (predicted discontinuous epitopes) | | | | | | | |
| Residues | **Score** | **Residues** | **Score** | **Residues** | **Score** |  |  |
| K57, A58, A59, E60, E61, N62, N63, N64, V65, E66 | 0.873 | K2, E3, I4, D5, E6, S7, E8, D11, Y12, A13, K14, E15, G16, F17, R18, A19, P20, S23 | 0.725 | Y68, E71, G72, K75 | 0.715 |  |  |
| DiscoTope 2.0 | | | | | | | |
| Sequence |  | **Sequence** |  |  |  |  |  |
| LKEIDESESEDYAKEGFRAPLQS | | AAEENNNVEDYF |  |  |  |  |  |

### Supplementary Table S12. Predicted B cell epitopes in the B region of PspA clade 3 (AC122).

| LBTope* | | | | | | | | |
| --- | --- | --- | --- | --- | --- | --- | --- | --- |
| Sequence | **% Probabilty of correct prediction** | **Sequence** | | **% Probabilty of correct prediction** | **Sequence** | **% Probabilty of correct prediction** | **Sequence** | **% Probabilty of correct prediction** |
| DELDKEAEEAELDKK | 60.59 | DKEAEEAELDKKADE | | 73.82 | DELQNKVADLEKEIS | 64.94 | EKEISNLEILLGGAD | 60.47 |
| ELDKEAEEAELDKKA | 67.13 | KEAEEAELDKKADEL | | 61.92 | LQNKVADLEKEISNL | 64.40 |  |  |
| LDKEAEEAELDKKAD | 73.04 | ADELQNKVADLEKEI | | 61.30 | VADLEKEISNLEILL | 63.63 |  |  |
| ABCpred | | | | | | | | |
| Sequence | **Score** | **Sequence** | | **Score** | **Sequence** | **Score** | **Sequence** | **Score** |
| AELEKTQKELDAALNE | 0.95 | AEEAELDKKADELQNK | | 0.82 | EKLLDSLDPEGKTQDE | 0.73 | AKKQTELEKLLDSLDP | 0.55 |
| DPEGKTQDELDKEAEE | 0.85 | LGGADSEDDTAALQNK | | 0.78 | AALQNKLATKKAELEK | 0.64 |  |  |
| Emini surface accessibility Prediction | | | | | | | | |
| Sequence | | **Sequence** | | | **Sequence** | |  | |
| EGKTQDELDKEAE | | ELDKKAD | | | EKTQKE | |  | |
|  |  |  | |  |  |  |  |  |
| Ellipro (predicted linear epitopes) | | | | | | | | |
| Sequence | **Score** | | **Sequence** | **Score** | **Sequence** | **Score** |  |  |
| SNLEILLGGADSEDD | 0.709 | | TELEKLLDSLDPEGKTQDEL | 0.653 | KELDAALNE | 0.622 |  |  |
| Ellipro (predicted discontinuous epitopes) | | | | | | | | |
| Residues | **Score** | | **Residues** | **Score** | **Residues** | **Score** | **Residues** | **Score** |
| S53, N54, L55, E56, I57, L58, L59, G60, G61, A62, D63, S64, E65, D66, D67 | 0.709 | | K10, L11, L12, D13, S14, L15, D16, P17, E18, G19, K20, T21, Q22, D23, E24, L25, D26 | 0.679 | D90, A91, A92, L93, N94, E95 | 0.677 | A46, E49, K50 | 0.649 |
| DiscoTope 2.0 | | | | | | | | |
| Sequence |  | |  |  |  |  |  |  |
| KKQTELEKLLDSLDPEGKTQDE | | |  |  |  |  |  |  |

### Supplementary Table S13. Predicted B cell epitopes in the B region of PspA clade 4 (EF5668).

| LBTope* | | | | | | | | |
| --- | --- | --- | --- | --- | --- | --- | --- | --- |
| Sequence | **% Probabilty of correct prediction** | **Sequence** | | **% Probabilty of correct prediction** | **Sequence** | **% Probabilty of correct prediction** | **Sequence** | **% Probabilty of correct prediction** |
| DPEGKTQDELDKEAA | 61.55 | NQVAELEEELSKLED | | 65.74 | ELEEELSKLEDNLKD | 78.88 | EELSKLEDNLKDAET | 69.15 |
| NEKVEALQNQVAELE | 62.32 | QVAELEEELSKLEDN | | 64.53 | LEEELSKLEDNLKDA | 79.61 | ELSKLEDNLKDAETN | 63.01 |
| LQNQVAELEEELSKL | 62.89 | VAELEEELSKLEDNL | | 66.57 | EEELSKLEDNLKDAE | 78.33 | KLEDNLKDAETNNVE | 60.23 |
| ABCpred | | | | | | | | |
| Sequence | **Score** | **Sequence** | | **Score** | **Sequence** | **Score** |  |  |
| AELEKTQKELDAALNE | 0.95 | LEKVLATLDPEGKTQD | | 0.79 | ELSKLEDNLKDAETNN | 0.73 |  |  |
| DPEGKTQDELDKEAAE | 0.89 | EGLEEAIATKKAELEK | | 0.78 | EDAELELEKVLATLDP | 0.69 |  |  |
| VEDYIKEGLEEAIATK | 0.80 | QVAELEEELSKLEDNL | | 0.76 | EAAEAELNEKVEALQN | 0.63 |  |  |
| Emini surface accessibility Prediction | | | | | | | | |
| Sequence | | **Sequence** | | | **Sequence** | |  | |
| PEGKTQDELD | | NLKDAE | | | KKAELEKTQKE | |  | |
|  |  |  | |  |  |  |  |  |
| Ellipro (predicted linear epitopes) | | | | | | | | |
| Sequence | **Score** | | **Sequence** | **Score** |  |  |  |  |
| PEGKTQDELDKEAAEA | 0.758 | | DNLKDAETNNVEDYIKE | 0.749 |  |  |  |  |
| Ellipro (predicted discontinuous epitopes) | | | | | | | | |
| Residues | **Score** | | **Residues** | **Score** | **Residues** | **Score** | **Residues** | **Score** |
| D61, A62, E63, T64, N65, N66, V67, E68, D69, I71 | 0.828 | | P17, E18, G19, K20, T21, Q22, D23, E24, L25, D26, K27, E28, A29, A30, E31, A32 | 0.758 | D94, A95, L97, N98 | 0.722 | Y70, E73, E77 | 0.684 |
| DiscoTope 2.0 | | | | | | | | |
| Sequence |  | | **Sequence** |  |  |  |  |  |
| KTQDELDKE |  | | KDAETNNVEDYIKE |  |  |  |  |  |

### Supplementary Table S14. Predicted B-cell epitopes in the B region of PspA clade 5 (ATCC 6303).

| LBTope* | | | | | | | | |
| --- | --- | --- | --- | --- | --- | --- | --- | --- |
| Sequence | **% Probabilty of correct prediction** | **Sequence** | | **% Probabilty of correct prediction** | **Sequence** | **% Probabilty of correct prediction** | **Sequence** | **% Probabilty of correct prediction** |
| DPEGKTQDELDKEAA | 61.55 | QDELDKEAAEDANIE | | 81.37 | LENKVAELDKEVTRL | 74.49 | ENNVEDYVKEGLEKA | 61.77 |
| EGKTQDELDKEAAED | 65.40 | DELDKEAAEDANIEA | | 81.10 | ENKVAELDKEVTRLQ | 74.06 | NNVEDYVKEGLEKAL | 60.38 |
| GKTQDELDKEAAEDA | 76.32 | ELDKEAAEDANIEAL | | 64.59 | NKVAELDKEVTRLQS | 63.68 | EDYVKEGLEKALTDK | 68.01 |
| KTQDELDKEAAEDAN | 72.02 | VADLENKVAELDKEV | | 67.54 | AEENNVEDYVKEGLE | 64.37 | DYVKEGLEKALTDKK | 67.86 |
| TQDELDKEAAEDANI | 79.69 | DLENKVAELDKEVTR | | 67.52 | EENNVEDYVKEGLEK | 62.80 |  |  |
| ABCpred | | | | | | | | |
| Sequence | **Score** | **Sequence** | | **Score** | **Sequence** | **Score** | **Sequence** | **Score** |
| DPEGKTQDELDKEAAE | 0.89 | EVTRLQSDLKDAEENN | | 0.81 | EDAELELEKVLATLDP | 0.69 | ADLENKVAELDKEVTR | 0.58 |
| EGLEKALTDKKVELNN | 0.84 | LEKVLATLDPEGKTQD | | 0.79 | ELDKEAAEDANIEALQ | 0.68 | EDANIEALQNKVADLE | 0.58 |
| KALDTAPKALDTALNE | 0.81 | DKKVELNNTQKALDTA | | 0.70 | VEDYVKEGLEKALTDK | 0.64 |  |  |
| Emini surface accessibility Prediction | | | | | | | | |
| Sequence | | **Sequence** | | | **Sequence** | |  | |
| PEGKTQDELD | | LKDAEENN | | | NNTQKA | |  | |
|  |  |  | |  |  |  |  |  |
| Ellipro (predicted linear epitopes) | | | | | | | | |
| Sequence | **Score** | | **Sequence** | **Score** | **Sequence** | **Score** |  |  |
| PEGKTQDELDKEAAEDANI | 0.76 | | SDLKDAEENNVEDYVKEG | 0.755 | QKALDTAPK | 0.678 |  |  |
| Ellipro (predicted discontinuous epitopes) | | | | | | | | |
| Residues | **Score** | | **Residues** | **Score** | **Residues** | **Score** |  |  |
| D65, A66, E67, E68, N69, N70, V71, E72, D73, Y74, V75, K76, E77, G78, K81 | 0.776 | | P17, E18, G19, K20, T21, Q22, D23, E24, L25, D26, K27, E28, A29, A30, E31, D32, A33, N34, I35 | 0.76 | K95, A96, L97, D98, T99, A100, P101, K102, D105, T106, L108, N109 | 0.696 |  |  |
| DiscoTope 2.0 | | | | | | | | |
| Sequence |  | | **Sequence** |  |  |  |  |  |
| KTQDELDKEAAEDAN |  | | KDAEENNVEDYVKEG |  |  |  |  |  |

### Supplementary Table S15. Prediction of MHC-II epitopes in the B region of PspA protein from clades 1 to 5 (PspA1-5-B).

| PspA1-B  (DBL6A) | IEDB | | |  | | |
| --- | --- | --- | --- | --- | --- | --- |
|  | **Allele** | **Peptide** | **Percentile Rank** | **Allele** | **Peptide** | **Percentile Rank** |
|  | H2-IAb | YVKEGFRAPLQSELD | 8.60 | H2-IAb | DYVKEGFRAPLQSEL | 8.90 |
|  | **NetMHCIIpan** | | |  | | |
|  | **Allele** | **Peptide** | **%Rank** | **Allele** | **Peptide** | **%Rank** |
|  | H-2-IAb | KEGFRAPLQSELDAK | 0.95 | DRB1_0101 | SDKIDELDAEIAKLE | 0.59 |
|  | H-2-IAd | YLAAAEEDLIAKKAE | 0.61 | DRB1_0401 | VEDFKNSDGEQAGQY | 0.98 |
|  | H-2-IAd | LAAAEEDLIAKKAEL | 0.59 | DRB1_0801 | AAEEDLIAKKAELEQ | 0.95 |
|  | H-2-IAd | AAAEEDLIAKKAELE | 0.31 | DRB1_0801 | AEEDLIAKKAELEQT | 0.66 |
|  | DRB1_0101 | LSDKIDELDAEIAKL | 0.95 | DRB1_0801 | EEDLIAKKAELEQTE | 0.50 |
| PspA2-B  (R6) | **IEDB** | | |  | | |
|  | **Allele** | **Peptide** | **Percentile Rank** | **Allele** | **Peptide** | **Percentile Rank** |
|  | H-2-IAd | EGLEKTIAAKKAELE | 5.00 | H2-IAb | AKEGFRAPLQSKLDA | 6.95 |
|  | H-2-IAd | GLEKTIAAKKAELEK | 5.85 | H-2-IAd | KEGLEKTIAAKKAEL | 7.15 |
|  | H2-IAb | YAKEGFRAPLQSKLD | 6.50 | H2-IAb | KEGFRAPLQSKLDAK | 7.90 |
|  | H2-IAb | DYAKEGFRAPLQSKL | 6.60 | H-2-IAd | FKEGLEKTIAAKKAE | 9.00 |
|  | **NetMHCIIpan** | | |  | | |
|  | **Allele** | **Peptide** | **%Rank** | **Allele** | **Peptide** | **%Rank** |
|  | H-2-IEd | EKTIAAKKAELEKTE | 0.54 | H-2-IAd | GLEKTIAAKKAELEK | 0.08 |
|  | H-2-IEd | LEKTIAAKKAELEKT | 0.76 | DRB1_0101 | LSDKIDELDAEIAKL | 0.95 |
|  | H-2-IEd | KEGLEKTIAAKKAEL | 0.69 | DRB1_0101 | SDKIDELDAEIAKLE | 0.59 |
|  | H-2-IEd | FKEGLEKTIAAKKAE | 0.40 | DRB1_0801 | GLEKTIAAKKAELEK | 0.55 |
|  | H-2-IAd | YFKEGLEKTIAAKKA | 0.46 | DRB1_0801 | LEKTIAAKKAELEKT | 0.30 |
|  | H-2-IAd | FKEGLEKTIAAKKAE | 0.02 | DRB1_0801 | EKTIAAKKAELEKTE | 0.38 |
|  | H-2-IAd | KEGLEKTIAAKKAEL | 0.01 | DRB1_0801 | KTIAAKKAELEKTEA | 0.88 |
|  | H-2-IAd | EGLEKTIAAKKAELE | 0.00 |  |  |  |
| PspA3-B  (AC122) | **NetMHCIIpan** | | |  | | |
|  | **Allele** | **Peptide** | **%Rank** | **Allele** | **Peptide** | **%Rank** |
|  | DRB1_0801 | QNKLATKKAELEKTQ | 0.81 | DRB1_0801 | KKAELEKTQKELDAA | 0.73 |
| PspA4-B  (EF5668) | **NetMHCIIpan** | | |  | | |
|  | **Allele** | **Peptide** | **%Rank** | **Allele** | **Peptide** | **%Rank** |
|  | H-2-IEd | EEAIATKKAELEKTQ | 0.75 | DRB1_0101 | NEKVEALQNQVAELE | 0.93 |
|  | H-2-IAd | YIKEGLEEAIATKKA | 0.89 | DRB1_0401 | NEKVEALQNQVAELE | 0.96 |
|  | H-2-IAd | IKEGLEEAIATKKAE | 0.06 | DRB1_0801 | GLEEAIATKKAELEK | 0.97 |
|  | H-2-IAd | KEGLEEAIATKKAEL | 0.05 | DRB1_0801 | LEEAIATKKAELEKT | 0.68 |
|  | H-2-IAd | EGLEEAIATKKAELE | 0.02 | DRB1_0801 | EEAIATKKAELEKTQ | 0.56 |
|  | H-2-IAd | GLEEAIATKKAELEK | 0.19 | DRB1_0801 | KKAELEKTQKELDAA | 0.73 |
| PspA5-B  (ATCC 6303) | **IEDB** | | |  | | |
|  | **Allele** | **Peptide** | **Percentile Rank** | **Allele** | **Peptide** | **Percentile Rank** |
|  | DRB1*03:01 | VTRLQSDLKDAEENN | 3.30 | DRB1*03:01 | DKEVTRLQSDLKDAE | 5.70 |
|  | DRB1*03:01 | EVTRLQSDLKDAEEN | 3.40 | DRB1*03:01 | RLQSDLKDAEENNVE | 6.90 |
|  | DRB1*03:01 | KEVTRLQSDLKDAEE | 4.10 | DRB1*03:01 | LQSDLKDAEENNVED | 7.20 |
|  | DRB1*03:01 | TRLQSDLKDAEENNV | 4.50 |  |  |  |
|  | **NetMHCIIpan** | | |  | | |
|  | **Allele** | **Peptide** | **%Rank** | **Allele** | **Peptide** | **%Rank** |
|  | H-2-IAd | AAEDANIEALQNKVA | 0.72 | DRB1_0801 | KVELNNTQKALDTAP | 0.43 |
|  | H-2-IAd | AEDANIEALQNKVAD | 0.99 | DRB1_0801 | TQKALDTAPKALDTA | 0.61 |
|  | DRB1_0101 | DANIEALQNKVADLE | 0.72 | DRB1_0801 | QKALDTAPKALDTAL | 0.83 |
|  | DRB1_0301 | EVTRLQSDLKDAEEN | 0.75 | DRB1_1501 | AEDANIEALQNKVAD | 0.96 |
|  | DRB1_0301 | VTRLQSDLKDAEENN | 0.53 | DRB1_1501 | EDANIEALQNKVADL | 0.80 |
|  | DRB1_0801 | KKVELNNTQKALDTA | 0.79 | DRB1_1501 | DANIEALQNKVADLE | 0.62 |

### Supplementary Table S16. Predicted linear B-cell epitopes of PhtD-C.

| LBTope* | | | | | | | | |
| --- | --- | --- | --- | --- | --- | --- | --- | --- |
| Sequence | **% Probabilty of correct prediction** | **Sequence** | | **% Probabilty of correct prediction** | **Sequence** | **% Probabilty of correct prediction** | **Sequence** | **% Probabilty of correct prediction** |
| KVGDGYVFEENGVPR | 62.17 | APIRHPERLGKPNAQ | | 62.70 | FEWFDEGLYEAPKGY | 64.84 | EDKEHDEVSEPTHPE | 60.76 |
| VGDGYVFEENGVPRY | 62.30 | PIRHPERLGKPNAQI | | 60.66 | EWFDEGLYEAPKGYS | 65.38 | DKEHDEVSEPTHPES | 63.36 |
| GDGYVFEENGVPRYI | 68.29 | IFDPRDITSDEGDAY | | 61.34 | GLYEAPKGYSLEDLL | 62.65 | KEHDEVSEPTHPESD | 72.74 |
| DGYVFEENGVPRYIP | 74.69 | FDPRDITSDEGDAYV | | 65.07 | LYEAPKGYSLEDLLA | 71.19 | EHDEVSEPTHPESDE | 56.97 |
| GYVFEENGVPRYIPA | 62.80 | WIKKDSLSEAERAAA | | 66.66 | YEAPKGYSLEDLLAT | 61.84 | HDEVSEPTHPESDEK | 58.14 |
| PRYIPAKDLSAETAA | 61.01 | IKKDSLSEAERAAAQ | | 69.18 | EAPKGYSLEDLLATV | 60.18 | DEVSEPTHPESDEKE | 65.29 |
| TAAGIDSKLAKQESL | 61.55 | AYAKEKGLTPPSTDH | | 60.71 | PKGYSLEDLLATVKY | 62.42 | EVSEPTHPESDEKEN | 68.67 |
| AAGIDSKLAKQESLS | 61.43 | KGAEAIYNRVKAAKK | | 60.45 | GYSLEDLLATVKYYV | 64.28 | VSEPTHPESDEKENH | 66.09 |
| AGIDSKLAKQESLSH | 62.01 | AEAIYNRVKAAKKVP | | 63.69 | YSLEDLLATVKYYVE | 70.99 | SEPTHPESDEKENHV | 64.58 |
| GIDSKLAKQESLSHK | 67.50 | EAIYNRVKAAKKVPL | | 67.67 | SLEDLLATVKYYVEH | 71.99 | EPTHPESDEKENHVG | 78.08 |
| IDSKLAKQESLSHKL | 65.33 | AIYNRVKAAKKVPLD | | 64.22 | LEDLLATVKYYVEHP | 73.19 | PTHPESDEKENHVGL | 88.62 |
| DSKLAKQESLSHKLG | 70.50 | AKKVPLDRMPYNLQY | | 62.98 | EDLLATVKYYVEHPN | 78.23 | THPESDEKENHVGLN | 76.27 |
| SKLAKQESLSHKLGA | 76.16 | VPLDRMPYNLQYTVE | | 71.53 | DLLATVKYYVEHPNE | 76.15 | HPESDEKENHVGLNP | 74.54 |
| KLAKQESLSHKLGAK | 65.82 | PLDRMPYNLQYTVEV | | 72.03 | LLATVKYYVEHPNER | 66.77 | PESDEKENHVGLNPS | 77.70 |
| LAKQESLSHKLGAKK | 65.00 | LDRMPYNLQYTVEVK | | 77.77 | ATVKYYVEHPNERPH | 71.91 | ESDEKENHVGLNPSA | 76.47 |
| SHKLGAKKTDLPSSD | 64.17 | DRMPYNLQYTVEVKN | | 72.07 | TVKYYVEHPNERPHS | 73.60 | SDEKENHVGLNPSAD | 73.15 |
| LGAKKTDLPSSDREF | 65.00 | RMPYNLQYTVEVKNG | | 71.80 | VKYYVEHPNERPHSD | 74.39 | DEKENHVGLNPSADN | 60.92 |
| TDLPSSDREFYNKAY | 63.93 | MPYNLQYTVEVKNGS | | 72.71 | KYYVEHPNERPHSDN | 60.65 | PSADNLYKPSTDTEE | 61.39 |
| EFYNKAYDLLARIHQ | 64.42 | PYNLQYTVEVKNGSL | | 66.99 | VEHPNERPHSDNGFG | 65.58 | TDEAEIPQVEHSVIN | 61.07 |
| FYNKAYDLLARIHQD | 63.06 | YNLQYTVEVKNGSLI | | 63.80 | ASDHVQRNKNGQADT | 63.73 | IRQNAVETLTGLKSS | 69.63 |
| YNKAYDLLARIHQDL | 71.92 | NLQYTVEVKNGSLII | | 62.61 | SDHVQRNKNGQADTN | 80.34 | RQNAVETLTGLKSSL | 78.04 |
| NKAYDLLARIHQDLL | 62.76 | LQYTVEVKNGSLIIP | | 61.58 | DHVQRNKNGQADTNQ | 69.28 | QNAVETLTGLKSSLL | 68.19 |
| KAYDLLARIHQDLLD | 62.81 | QYTVEVKNGSLIIPH | | 64.34 | QRNKNGQADTNQTEK | 71.60 | LKSSLLLGTKDNNTI | 61.49 |
| AYDLLARIHQDLLDN | 68.17 | YTVEVKNGSLIIPHY | | 64.80 | RNKNGQADTNQTEKP | 70.27 | KSSLLLGTKDNNTIS | 60.55 |
| YDLLARIHQDLLDNK | 68.29 | TVEVKNGSLIIPHYD | | 64.16 | NKNGQADTNQTEKPN | 71.94 | SSLLLGTKDNNTISA | 63.19 |
| DLLARIHQDLLDNKG | 74.91 | VEVKNGSLIIPHYDH | | 61.67 | KNGQADTNQTEKPNE | 68.52 | SLLLGTKDNNTISAE | 61.01 |
| LLARIHQDLLDNKGR | 71.56 | EVKNGSLIIPHYDHY | | 62.31 | NGQADTNQTEKPNEE | 60.57 | LLLGTKDNNTISAEV | 66.68 |
| LARIHQDLLDNKGRQ | 78.06 | VKNGSLIIPHYDHYH | | 61.98 | EEKPQTEKPEEDKEH | 62.24 | LLGTKDNNTISAEVD | 76.60 |
| ARIHQDLLDNKGRQV | 69.86 | IIPHYDHYHNIKFEW | | 72.02 | EKPQTEKPEEDKEHD | 61.25 | LGTKDNNTISAEVDS | 78.98 |
| IHQDLLDNKGRQVDF | 60.62 | IPHYDHYHNIKFEWF | | 64.10 | KPQTEKPEEDKEHDE | 73.39 | GTKDNNTISAEVDSL | 80.97 |
| HQDLLDNKGRQVDFE | 65.15 | PHYDHYHNIKFEWFD | | 68.93 | PQTEKPEEDKEHDEV | 72.55 | TKDNNTISAEVDSLL | 73.94 |
| ALDNLLERLKDVSSD | 61.23 | HYDHYHNIKFEWFDE | | 68.13 | QTEKPEEDKEHDEVS | 74.97 | KDNNTISAEVDSLLA | 66.53 |
| LDNLLERLKDVSSDK | 73.89 | YDHYHNIKFEWFDEG | | 72.78 | TEKPEEDKEHDEVSE | 62.91 | LALLKESQPTPIQ | 62.40 |
| DNLLERLKDVSSDKV | 69.96 | DHYHNIKFEWFDEGL | | 70.32 | KPEEDKEHDEVSEPT | 68.83 | ALLKESQPTPIQ | 64.24 |
| NLLERLKDVSSDKVK | 67.03 | IKFEWFDEGLYEAPK | | 67.72 | PEEDKEHDEVSEPTH | 68.26 |  |  |
| LERLKDVSSDKVKLV | 62.39 | KFEWFDEGLYEAPKG | | 60.93 | EEDKEHDEVSEPTHP | 71.35 |  |  |
| ABCpred | | | | | | | | |
| Sequence | **Score** | **Sequence** | | **Score** | **Sequence** | **Score** | **Sequence** | **Score** |
| EKPQTEKPEEDKEHDE | 0.95 | FGNASDHVQRNKNGQA | | 0.87 | HVQRNKNGQADTNQTE | 0.80 | GQADTNQTEKPNEEKP | 0.72 |
| DPRDITSDEGDAYVTP | 0.95 | TDLPSSDREFYNKAYD | | 0.86 | PEEDKEHDEVSEPTHP | 0.79 | AYAKEKGLTPPSTDHQ | 0.71 |
| TDHQDSGNTEAKGAEA | 0.93 | HSVINAKIAEAEALLE | | 0.86 | NGVPRYIPAKDLSAET | 0.79 | LLGTKDNNTISAEVDS | 0.69 |
| EVSEPTHPESDEKENH | 0.92 | EGLYEAPKGYSLEDLL | | 0.86 | SHWIKKDSLSEAERAA | 0.79 | SSIRQNAVETLTGLKS | 0.69 |
| HYHNIKFEWFDEGLYE | 0.92 | GAEAIYNRVKAAKKVP | | 0.85 | KPNAQITYTDDEIQVA | 0.79 | SAEVDSLLALLKESQP | 0.66 |
| GLTPPSTDHQDSGNTE | 0.91 | ERAAAQAYAKEKGLTP | | 0.85 | LARIHQDLLDNKGRQV | 0.76 | ERLKDVSSDKVKLVDD | 0.66 |
| KYTTEDGYIFDPRDIT | 0.89 | TGLKSSLLLGTKDNNT | | 0.84 | EEAEDTTDEAEIPQVE | 0.76 | KAAKKVPLDRMPYNLQ | 0.65 |
| LAPIRHPERLGKPNAQ | 0.89 | SDEGDAYVTPHMTHSH | | 0.84 | YVTPHMTHSHWIKKDS | 0.75 | RMPYNLQYTVEVKNGS | 0.60 |
| VRKVGDGYVFEENGVP | 0.88 | DFEALDNLLERLKDVS | | 0.83 | GLNPSADNLYKPSTDT | 0.74 | KGYSLEDLLATVKYYV | 0.53 |
| VEHPNERPHSDNGFGN | 0.88 | PSTDTEETEEEAEDTT | | 0.83 | SDEKENHVGLNPSADN | 0.74 |  |  |
| GSLIIPHYDHYHNIKF | 0.88 | KLAKQESLSHKLGAKK | | 0.81 | LSAETAAGIDSKLAKQ | 0.74 |  |  |
| DDEIQVAKLAGKYTTE | 0.88 | AEALLEKVTDSSIRQN | | 0.81 | EIPQVEHSVINAKIAE | 0.72 |  |  |
| Emini surface accessibility Prediction | | | | | | | | |
| Sequence Sequence Sequence Sequence | | | | | | | | |
| SSDREFYNKA | | PSTDHQDSG | | | HPNERPHS | | HVQRNKNG | |
| DTNQTEKPNEEKPQTEKPEEDKEH | | PTHPESDEKE | | | NLYKPSTDTEETEEEAEDTTD | |  | |
|  |  |  | |  |  |  |  |  |
| Ellipro | | | | | | | | |
| Sequence | **Score** | | **Sequence** | **Score** | **Sequence** | **Score** | **Sequence** | **Score** |
| LEKVTDSSIRQNAVETLTGLKSSLLLGTKDNNTISAEVDSLLALLKESQPTPIQ | 0.826 | | EAKGAEAIYNRVKAAK | 0.638 | SDHVQRNKNGQADTNQTEK | 0.605 | EPTH | 0.555 |
| EKLVKEAVRKVGDGYVFEENGVPRYIPAKDLSAETAAGIDSKLAKQESL | 0.801 | | EHPNERPHSDNGF | 0.611 | DLLDNKGRQVDFEALDNLLERLKDVSSDKVKLVDDILAFLAPIR | 0.589 | LIIPHYDH | 0.546 |
| TEETEEEAEDTTDEAEIPQVEH | 0.79 | | KTDLPSSDREFYN | 0.608 | AKEKGL | 0.558 | DEGLY | 0.527 |

### Supplementary Table S17. Predicted discontinuous B-cell epitopes from PhtD-C.

| Ellipro |  |  |
| --- | --- | --- |
| Residues | **Number of residues** | **Score** |
| E1, K2, L3, V4, K5, E6 | 6 | 0.975 |
| E298, H299, P300, N301, E302, R303, P304, H305, S306, D307, N308, G309, F310, N334, E354, P355, T356, H357, P358, H366, V367, G368, L369, T383, E384, E385, T386, E387, E388, E389, A390, E391, D392, T393, T394, D395, E396, A397, E398, I399, P400, Q401, V402, E403, H404, S405, N408, L418, E419, K420, V421, T422, D423, S424, S425, I426, R427, Q428, N429, A430, V431, E432, T433, L434, T435, G436, L437, K438, S439, S440, L441, L442, L443, G444, T445, K446, D447, N448, N449, T450, I451, S452, A453, E454, V455, D456, S457, L458, L459, A460, L461, L462, K463, E464, S465, Q466, P467, T468, P469, I470, Q471 | 101 | 0.746 |
| A7, V8, K10, V11, G12, D13, G14, Y15, V16, F17, E18, E19, N20, G21, V22, P23, R24, Y25, I26, P27, A28, K29, D30, L31, S32, A33, E34, T35, A36, A37, G38, I39, D40, S41, K42, L43, A44, K45, Q46, E47, S48, L49, A55, K56, K57, T58, D59, L60, P61, S62, S63, D64, R65, E66, F67, Y68, N69, K70, K103, D104, V105, S106, S107, D108, K109, K111, L112, V113, D114, D115, I116, A118, F119, L120, A121, P122, I123, H125, E127, Q144, K147 | 81 | 0.667 |
| Y158, P162, R163, I165, T166, K204, G205, L206, T207, G217, N218, E220, A221, K222, G223, A224, E225, A226, I227, Y228, N229, R230, V231, K232, A233, A234, K235, D274, E275, G276, L277, Y278, N322, G323, Q324, A325, D326, T327, N328, Q329, T330, E331, K332 | 43 | 0.608 |
| D81, L82, L83, D84, N85, K86, G87, R88, Q89, V90, D91, F92, E93, A94, L95, D96, N97, L98, L99, R101, E169, A172, Y173, V174, T175, P176, H177, H182, Y200, A201, K202, E203 | 32 | 0.584 |
| S256, L257, I258, I259, P260, H261, Y262, D263, H264, S314, D315, H316, V317, N320, K321 | 15 | 0.526 |
| Discotope |  |  |
| Residues | **Number of residues** | **Score** |
| T175, H177, M178, T179, H180, S181, H182, W183, K185, K186, D187, S188, L189, S190, E191, A192, E193, R194, A195, A196, A197, Q198, A199, Y200, A201, K202, E203, K204, G205, L206, T207, P208, P209, S210, T211, D212, H213, Q214, D215, S216, G217, N218, T219, E220, A221, K222, G223, A224, E225, A226, I227, Y228, N229, R230, V231, K232, A233, A234, K235, K236, V237, P238, L239, D240, R241, M242, P243, Y244, N245, L246, Q247, Y248, T249, V250, E251, V252, K253, N254, G255, S256, L257, I258, I259, P260, H261, Y262, D263, H264, Y265, H266, N267, I268, K269, F270, E271, W272, F273, D274, E275, G276, L277, Y278, E279, A280, P281, K282, G283, Y284, S285, L286, E287, D288, L289, L290, A291, T292, V293, K294, Y295, Y296, V297, E298, H299, P300, N301, E302, R303, P304, H305, S306, D307, N308, G309, F310, G311, N312, A313, S314, D315, H316, V317, Q318, R319, N320, K321, N322, G323, Q324, A325, D326, T327, N328, Q329, T330, E331, K332, P333, N334, E335, E336, K337, P338, Q339, T340, E341, K342, P343, E344, E345, D346, K347, E348, H349, D350, E351, V352, S353, E354, P355, T356, H357, P358, E359, S360, D361, E362, K363, E6364, N365 | 189 | 3.71 |
| N370, S372, A373, D374, N375, L376, Y377, K378, P379 | 9 | -0.85 |
| T386, E387, E388, E389, A390, E391, D392, T393, T394, D395, E396, E398 | 12 | -0.89 |
| E19, G21, V22, P23, R24, Y25 | 6 | -1.01 |
| K45, E47, S48, L49, S50 | 5 | -1.49 |
| K86, G87, R88, Q89 | 4 | -1.53 |
| F67, N69, K70, A71 | 4 | -1.80 |
| D141, I143, V145, A146, L148, G150, Y152, T154, E155, D156, G157, Y158, I159, F160, D161, P162, R163, D164, I165, T166, D168, E169 | 22 | -1.86 |
| I123, R124, H125, E127, R128, L129 | 6 | -1.98 |
| E1, K2, L3, V4, K5, E6, A7, V8 | 8 | -2.22 |
| D447, N448, N449, T450, I451, S452, A453, E454 | 8 | -2.33 |

### Supplementary Table S18. Prediction of MHC-II epitopes from PhtD-C. Epitopes with IEDB percentile rank <10.0 and NetMHCIIpan rank value <1.0 were considered for further analysis.

| IEDB | | |  | | | | |
| --- | --- | --- | --- | --- | --- | --- | --- |
| Allele | **Peptide** | **Percentile Rank** | **Allele** | **Peptide** | | **Percentile Rank** | |
| H2-IEd | AEAIYNRVKAAKKVP | 2.40 | DRB1*11:01 | AEAIYNRVKAAKKVP | | 4.50 | |
| H2-IEd | GAEAIYNRVKAAKKV | 3.00 | DRB1*11:01 | EAIYNRVKAAKKVPL | | 4.60 | |
| H2-IEd | KGAEAIYNRVKAAKK | 3.25 | DRB1*11:01 | GAEAIYNRVKAAKKV | | 4.60 | |
| H2-IEd | EAIYNRVKAAKKVPL | 3.50 | DRB1*03:01 | RDITSDEGDAYVTPH | | 4.60 | |
| H2-IAd | QNAVETLTGLKSSLL | 4.30 | DRB1*04:01 | DSKLAKQESLSHKLG | | 4.70 | |
| H2-IAd | NAVETLTGLKSSLLL | 4.40 | DRB1*04:01 | SKLAKQESLSHKLGA | | 4.70 | |
| H2-IEd | MTHSHWIKKDSLSEA | 4.85 | DRB1*04:01 | IDSKLAKQESLSHKL | | 4.80 | |
| H2-IAb | DEGDAYVTPHMTHSH | 5.00 | DRB1*11:01 | AIYNRVKAAKKVPLD | | 4.90 | |
| H2-IEd | HMTHSHWIKKDSLSE | 5.00 | DRB1*04:01 | GIDSKLAKQESLSHK | | 5.50 | |
| H2-IEd | ILAFLAPIRHPERLG | 5.10 | DRB1*03:01 | SDKVKLVDDILAFLA | | 5.50 | |
| H2-IAb | EGDAYVTPHMTHSHW | 5.20 | DRB1*07:01 | DSLLALLKESQPTPI | | 5.60 | |
| H2-IEd | AIYNRVKAAKKVPLD | 5.80 | DRB1*13:01 | EKLVKEAVRKVGDGY | | 5.70 | |
| H2-IAd | AVETLTGLKSSLLLG | 6.05 | DRB1*13:01 | KLVKEAVRKVGDGYV | | 5.70 | |
| H2-IEd | IYNRVKAAKKVPLDR | 6.05 | DRB1*13:01 | LVKEAVRKVGDGYVF | | 5.70 | |
| H2-IAb | SDEGDAYVTPHMTHS | 6.20 | DRB1*03:01 | DKVKLVDDILAFLAP | | 5.90 | |
| H2-IAd | LSEAERAAAQAYAKE | 6.25 | DRB1*03:01 | KVKLVDDILAFLAPI | | 6.20 | |
| H2-IEd | AKGAEAIYNRVKAAK | 6.30 | DRB1*04:01 | GDGYVFEENGVPRYI | | 6.30 | |
| H2-IAb | GDAYVTPHMTHSHWI | 6.40 | DRB1*11:01 | IYNRVKAAKKVPLDR | | 6.50 | |
| H2-IAd | SEAERAAAQAYAKEK | 6.50 | DRB1*01:01 | LTGLKSSLLLGTKDN | | 6.80 | |
| H2-IAb | VDDILAFLAPIRHPE | 6.50 | DRB1*11:01 | KGAEAIYNRVKAAKK | | 6.90 | |
| H2-IAb | DDILAFLAPIRHPER | 6.65 | DRB1*03:01 | SSDKVKLVDDILAFL | | 6.90 | |
| H2-IEd | YNRVKAAKKVPLDRM | 6.70 | DRB1*15:01 | VKLVDDILAFLAPIR | | 7.20 | |
| H2-IAd | SLSEAERAAAQAYAK | 6.85 | DRB1*07:01 | AIYNRVKAAKKVPLD | | 7.40 | |
| H2-IEd | LAFLAPIRHPERLGK | 7.20 | DRB1*04:01 | DGYVFEENGVPRYIP | | 7.40 | |
| H2-IAb | DAYVTPHMTHSHWIK | 7.70 | DRB1*07:01 | EAIYNRVKAAKKVPL | | 7.40 | |
| H2-IAb | DILAFLAPIRHPERL | 7.80 | DRB1*08:01 | AEAIYNRVKAAKKVP | | 7.60 | |
| H2-IEd | THSHWIKKDSLSEAE | 8.10 | DRB1*08:01 | AIYNRVKAAKKVPLD | | 7.60 | |
| H2-IAd | AIYNRVKAAKKVPLD | 8.15 | DRB1*08:01 | AKGAEAIYNRVKAAK | | 7.60 | |
| H2-IEd | NRVKAAKKVPLDRMP | 8.15 | DRB1*08:01 | EAIYNRVKAAKKVPL | | 7.60 | |
| H2-IAb | GAEAIYNRVKAAKKV | 8.20 | DRB1*08:01 | GAEAIYNRVKAAKKV | | 7.60 | |
| H2-IAb | AEAIYNRVKAAKKVP | 8.25 | DRB1*08:01 | IYNRVKAAKKVPLDR | | 7.60 | |
| H2-IEd | DILAFLAPIRHPERL | 8.35 | DRB1*08:01 | KGAEAIYNRVKAAKK | | 7.60 | |
| H2-IAd | EAERAAAQAYAKEKG | 8.35 | DRB1*11:01 | DDILAFLAPIRHPER | | 8.00 | |
| H2-IAd | VETLTGLKSSLLLGT | 8.35 | DRB1*11:01 | DEIQVAKLAGKYTTE | | 8.00 | |
| H2-IEd | AFLAPIRHPERLGKP | 8.65 | DRB1*11:01 | DILAFLAPIRHPERL | | 8.00 | |
| H2-IEd | PHMTHSHWIKKDSLS | 8.70 | DRB1*11:01 | ILAFLAPIRHPERLG | | 8.00 | |
| H2-IAb | GNTEAKGAEAIYNRV | 8.90 | DRB1*11:01 | LAFLAPIRHPERLGK | | 8.00 | |
| H2-IAb | SGNTEAKGAEAIYNR | 8.90 | DRB1*03:01 | VKLVDDILAFLAPIR | | 8.00 | |
| H2-IAb | DSGNTEAKGAEAIYN | 8.95 | DRB1*08:01 | EENGVPRYIPAKDLS | | 8.10 | |
| H2-IAd | EAIYNRVKAAKKVPL | 9.10 | DRB1*08:01 | ENGVPRYIPAKDLSA | | 8.10 | |
| H2-IAb | KGAEAIYNRVKAAKK | 9.10 | DRB1*08:01 | FEENGVPRYIPAKDL | | 8.10 | |
| H2-IEd | RVKAAKKVPLDRMPY | 9.50 | DRB1*08:01 | GVPRYIPAKDLSAET | | 8.10 | |
| H2-IEd | DDILAFLAPIRHPER | 9.80 | DRB1*08:01 | NGVPRYIPAKDLSAE | | 8.10 | |
| H2-IAb | SLLALLKESQPTPIQ | 9.85 | DRB1*03:01 | RLKDVSSDKVKLVDD | | 8.10 | |
| H2-IAb | LVDDILAFLAPIRHP | 9.95 | DRB1*08:01 | VFEENGVPRYIPAKD | | 8.10 | |
| DRB1*01:01 | AVETLTGLKSSLLLG | 0.44 | DRB1*08:01 | VPRYIPAKDLSAETA | | 8.10 | |
| DRB1*01:01 | NAVETLTGLKSSLLL | 0.51 | DRB1*15:01 | ETLTGLKSSLLLGTK | | 8.20 | |
| DRB1*01:01 | VETLTGLKSSLLLGT | 0.52 | DRB1*07:01 | GAEAIYNRVKAAKKV | | 8.20 | |
| DRB1*01:01 | ETLTGLKSSLLLGTK | 0.96 | DRB1*07:01 | IYNRVKAAKKVPLDR | | 8.20 | |
| DRB1*01:01 | QNAVETLTGLKSSLL | 1.60 | DRB1*07:01 | YNRVKAAKKVPLDRM | | 8.20 | |
| DRB1*08:01 | HMTHSHWIKKDSLSE | 1.70 | DRB1*04:01 | VGDGYVFEENGVPRY | | 8.30 | |
| DRB1*08:01 | HSHWIKKDSLSEAER | 1.70 | DRB1*01:01 | AEAIYNRVKAAKKVP | | 8.40 | |
| DRB1*08:01 | HWIKKDSLSEAERAA | 1.70 | DRB1*01:01 | AIYNRVKAAKKVPLD | | 8.40 | |
| DRB1*08:01 | MTHSHWIKKDSLSEA | 1.70 | DRB1*01:01 | EAIYNRVKAAKKVPL | | 8.40 | |
| DRB1*08:01 | SHWIKKDSLSEAERA | 1.70 | DRB1*01:01 | GAEAIYNRVKAAKKV | | 8.40 | |
| DRB1*08:01 | THSHWIKKDSLSEAE | 1.70 | DRB1*01:01 | KGAEAIYNRVKAAKK | | 8.40 | |
| DRB1*08:01 | WIKKDSLSEAERAAA | 1.70 | DRB1*15:01 | VETLTGLKSSLLLGT | | 8.60 | |
| DRB1*01:01 | TLTGLKSSLLLGTKD | 2.10 | DRB1*15:01 | ILAFLAPIRHPERLG | | 8.70 | |
| DRB1*11:01 | YNKAYDLLARIHQDL | 2.50 | DRB1*03:01 | ERLKDVSSDKVKLVD | | 8.90 | |
| DRB1*11:01 | FYNKAYDLLARIHQD | 2.60 | DRB1*15:01 | EDLLATVKYYVEHPN | | 9.00 | |
| DRB1*11:01 | NKAYDLLARIHQDLL | 2.70 | DRB1*03:01 | NTISAEVDSLLALLK | | 9.10 | |
| DRB1*11:01 | KAYDLLARIHQDLLD | 2.90 | DRB1*11:01 | YNRVKAAKKVPLDRM | | 9.10 | |
| DRB1*03:01 | DPRDITSDEGDAYVT | 3.00 | DRB1*07:01 | AEAIYNRVKAAKKVP | | 9.20 | |
| DRB1*11:01 | EFYNKAYDLLARIHQ | 3.20 | DRB1*11:01 | EKLVKEAVRKVGDGY | | 9.20 | |
| DRB1*15:01 | VDDILAFLAPIRHPE | 3.30 | DRB1*03:01 | NNTISAEVDSLLALL | | 9.20 | |
| DRB1*15:01 | DDILAFLAPIRHPER | 3.40 | DRB1*11:01 | VDDILAFLAPIRHPE | | 9.30 | |
| DRB1*03:01 | PRDITSDEGDAYVTP | 3.40 | DRB1*04:01 | GYVFEENGVPRYIPA | | 9.40 | |
| DRB1*11:01 | AYDLLARIHQDLLDN | 3.50 | DRB1*03:01 | LKDVSSDKVKLVDDI | | 9.40 | |
| DRB1*11:01 | YDLLARIHQDLLDNK | 3.50 | DRB1*15:01 | AVETLTGLKSSLLLG | | 9.70 | |
| DRB1*15:01 | LVDDILAFLAPIRHP | 3.60 | DRB1*03:01 | DNNTISAEVDSLLAL | | 9.70 | |
| DRB1*03:01 | FDPRDITSDEGDAYV | 4.10 | DRB1*03:01 | SHWIKKDSLSEAERA | | 9.70 | |
| DRB1*15:01 | KLVDDILAFLAPIRH | 4.10 | DRB1*15:01 | EAIYNRVKAAKKVPL | | 9.90 | |
| DRB1*15:01 | DILAFLAPIRHPERL | 4.30 | DRB1*03:01 | HSHWIKKDSLSEAER | | 9.90 | |
| DRB1*07:01 | SLLALLKESQPTPIQ | 4.30 |  |  | |  | |
| NetMHCIIpan |  |  |  |  |  | |  |
| Allele | **Peptide** | **%Rank** | **Allele** | **Peptide** | **%Rank** | |  |
| H-2-IEd | VPRYIPAKDLSAETA | 0.66 | DRB1_0401 | VGDGYVFEENGVPRY | 0.67 | |  |
| H-2-IEd | LATVKYYVEHPNERP | 0.21 | DRB1_0401 | GDGYVFEENGVPRYI | 0.80 | |  |
| H-2-IEd | ATVKYYVEHPNERPH | 0.05 | DRB1_0401 | NGVPRYIPAKDLSAE | 0.10 | |  |
| H-2-IEd | TVKYYVEHPNERPHS | 0.03 | DRB1_0401 | GVPRYIPAKDLSAET | 0.04 | |  |
| H-2-IEd | VKYYVEHPNERPHSD | 0.22 | DRB1_0401 | VPRYIPAKDLSAETA | 0.03 | |  |
| H-2-IAd | PRYIPAKDLSAETAA | 0.71 | DRB1_0401 | PRYIPAKDLSAETAA | 0.13 | |  |
| H-2-IAd | RYIPAKDLSAETAAG | 0.31 | DRB1_0401 | REFYNKAYDLLARIH | 0.68 | |  |
| H-2-IAd | YIPAKDLSAETAAGI | 0.37 | DRB1_0401 | GRQVDFEALDNLLER | 0.73 | |  |
| H-2-IAd | IPAKDLSAETAAGID | 0.81 | DRB1_0401 | RQVDFEALDNLLERL | 0.65 | |  |
| H-2-IAd | SAETAAGIDSKLAKQ | 0.47 | DRB1_0401 | QVDFEALDNLLERLK | 0.31 | |  |
| H-2-IAd | AETAAGIDSKLAKQE | 0.24 | DRB1_0401 | NAQITYTDDEIQVAK | 0.99 | |  |
| H-2-IAd | LSEAERAAAQAYAKE | 0.98 | DRB1_0401 | DRMPYNLQYTVEVKN | 0.90 | |  |
| H-2-IAd | SEAERAAAQAYAKEK | 0.69 | DRB1_0401 | RMPYNLQYTVEVKNG | 0.52 | |  |
| H-2-IAd | GNTEAKGAEAIYNRV | 0.77 | DRB1_0401 | DNGFGNASDHVQRNK | 0.70 | |  |
| H-2-IAd | NTEAKGAEAIYNRVK | 0.35 | DRB1_0801 | FYNKAYDLLARIHQD | 0.74 | |  |
| H-2-IAd | IPQVEHSVINAKIAE | 0.73 | DRB1_0801 | YNKAYDLLARIHQDL | 0.99 | |  |
| H-2-IAd | PQVEHSVINAKIAEA | 0.36 | DRB1_0801 | QVDFEALDNLLERLK | 0.77 | |  |
| H-2-IAd | QVEHSVINAKIAEAE | 0.23 | DRB1_0801 | HSHWIKKDSLSEAER | 0.72 | |  |
| H-2-IAd | VEHSVINAKIAEAEA | 0.35 | DRB1_1101 | EKLVKEAVRKVGDGY | 0.68 | |  |
| H-2-IAd | EHSVINAKIAEAEAL | 0.75 | DRB1_1101 | VPRYIPAKDLSAETA | 0.97 | |  |
| H-2-IAd | SIRQNAVETLTGLKS | 0.76 | DRB1_1101 | FYNKAYDLLARIHQD | 0.05 | |  |
| DRB1_0101 | PAKDLSAETAAGIDS | 0.78 | DRB1_1101 | YNKAYDLLARIHQDL | 0.06 | |  |
| DRB1_0101 | AKDLSAETAAGIDSK | 0.32 | DRB1_1101 | NKAYDLLARIHQDLL | 0.13 | |  |
| DRB1_0101 | DRMPYNLQYTVEVKN | 0.33 | DRB1_1101 | KAYDLLARIHQDLLD | 0.83 | |  |
| DRB1_0101 | RMPYNLQYTVEVKNG | 0.13 | DRB1_1101 | PYNLQYTVEVKNGSL | 0.65 | |  |
| DRB1_0301 | TEDGYIFDPRDITSD | 0.89 | DRB1_1101 | YNLQYTVEVKNGSLI | 0.46 | |  |
| DRB1_0301 | EDGYIFDPRDITSDE | 0.72 | DRB1_1101 | NLQYTVEVKNGSLII | 0.55 | |  |
| DRB1_0301 | FDPRDITSDEGDAYV | 0.70 | DRB1_1101 | VDSLLALLKESQPTP | 0.90 | |  |
| DRB1_0301 | DPRDITSDEGDAYVT | 0.41 | DRB1_1301 | KGAEAIYNRVKAAKK | 0.70 | |  |
| DRB1_0301 | PRDITSDEGDAYVTP | 0.30 | DRB1_1301 | GAEAIYNRVKAAKKV | 0.42 | |  |
| DRB1_0301 | RDITSDEGDAYVTPH | 0.85 | DRB1_1301 | AEAIYNRVKAAKKVP |  | |  |

### Supplementary Table S19. Consensus epitope sequences for B-region of PspAs. Two epitope sequences were considered for each clade. In the areas where there was sequence overlap, the consensus sequence was jointly selected.

| **Peptide name** | **Consensus epitope sequence for B-region of PspAs** | **AA of B regions** |
| --- | --- | --- |
| Cons-Clade 1 + 2 | LKEIDESDSEDYIKEGFRVPLQSELDAKR | 1-29 (Clade 1 & 2) |
| Cons-Clade 1 | KLEKDVEYFKNTDGEYTEQYLEAAEK | 51-76 (Clade 1) |
| Cons-Clade2 | LEDQLKDAEGNNNVEAYFKEGLEK | 52-76 (Clade 2) |
| Cons-Clade 3 + 4 | ELEKLLDTLDPEGKTQDELDKEAAEAELDKKV | 7-38 ( Clade 3 & 4) |
| Cons-Clade3 | KEISNLEILLGGADSEDDTAA | 54-74 ( Clade3) |
| Cons-Clade 4 + 5 | LTRLEDNLKDAEENNVEDYIKEGLEKAI | 56-83 (Clade 4 & 5) |
| Cons-Clade 5 | PEGKTQDELDKEAAEDANIEALQNKV | 17-42 (Clade 5) |

### Supplementary Table S20. Structural B-cell epitopes in the final construct PAD, PA, or PD predicted using the Ellipro tool.

| **Name** | **No.** | **Residues** | **Number of residues** | **Score** |
| --- | --- | --- | --- | --- |
| **PAD** | 1 | A:M1, A:E2, A:A3, A:P4, A:V5, A:A6, A:S7, A:Q8, A:S9, A:K10, A:A11, A:E12, A:K13, A:D14, A:Y15, A:D16, A:A17, A:A18, A:V19, A:K20, A:K21, A:S22, A:E23, A:A24, A:A25, A:K26, A:K27 | 27 | 0.887 |
|  | 2 | A:E52, A:K55, A:R56, A:E57, A:K58, A:E59, A:S61, A:E62, A:I64, A:A65, A:G66, A:G67, A:G68, A:S69, A:E70, A:L71, A:E72, A:K73, A:L74, A:L75, A:D76, A:T77, A:L78, A:D79, A:P80, A:E81, A:G82, A:K83, A:T84, A:Q85, A:D86, A:E87, A:L88, A:D89, A:K90, A:E91, A:A92, A:A93, A:E94 | 39 | 0.756 |
|  | 3 | A:R108, A:D111, A:N112, A:L113, A:K114, A:D115, A:A116, A:E117, A:E118, A:N119, A:N120, A:V121, A:E122, A:D123, A:Y124, A:I125, A:K126, A:E127, A:G128, A:L129 | 20 | 0.69 |
|  | 4 | A:P212, A:A213, A:P214, A:K215, A:G216, A:G217, A:S218, A:S219, A:G220, A:G221, A:K222, A:V223, A:T224, A:D225, A:S226, A:S227, A:I228, A:R229, A:Q230, A:N231, A:A232, A:V233, A:E234, A:P288, A:H289, A:S290, A:D291, A:N292, A:G293, A:F294, A:G295, A:N296, A:A297, A:S298, A:D299, A:H300, A:V301, A:Q302, A:R303, A:N304, A:K305, A:N306, A:G307, A:Q308, A:A309, A:D310, A:T311, A:N312, A:Q313, A:K316, A:P317, A:N318, A:E319, A:E320, A:K321, A:P322, A:G323, A:H324, A:H325, A:H326, A:H327, A:H328, A:H329 | 63 | 0.68 |
|  | 5 | A:L244, A:L245, A:G246, A:T247, A:K248, A:D249, A:N251, A:T252, A:I253, A:S254, A:A255, A:E256, A:V257, A:D258, A:S259, A:L261, A:A262, A:L263, A:K265, A:G266, A:G267, A:G268, A:S269, A:D272, A:L273, A:A275, A:T276, A:V277, A:K278, A:Y279 | 30 | 0.531 |
| **PA** | 1 | A:G67, A:G68, A:S69, A:E70, A:L71, A:E72, A:K73, A:L74, A:L75, A:D76, A:T77, A:L78, A:D79, A:P80, A:E81, A:G82, A:K83, A:T84, A:Q85, A:D86, A:E87, A:L88, A:D89, A:K90 | 24 | 0.792 |
|  | 2 | A:M1, A:E2, A:A3, A:P4, A:V5, A:A6, A:S7, A:Q8, A:S9, A:K10, A:A11, A:E12, A:K13, A:D14, A:Y15, A:D16, A:A17, A:A18, A:V19, A:K20, A:K21, A:S22, A:E23, A:A24, A:K26, A:K27 | 26 | 0.777 |
|  | 3 | A:R108, A:D111, A:N112, A:L113, A:K114, A:D115, A:A116, A:E117, A:E118, A:N119, A:N120, A:V121, A:E122, A:D123, A:Y124, A:I125, A:K126, A:E127 | 18 | 0.702 |
|  | 4 | A:G168, A:G169, A:S170, A:K171, A:L172, A:E173, A:K174, A:D175, A:V176, A:Y178, A:F179, A:K180, A:T182, A:D183, A:G184, A:E185, A:Y186, A:T187, A:E188, A:Q189, A:Y190, A:L191, A:E192, A:E195, A:P201, A:A202, A:P203, A:A204, A:P205, A:K206, A:P207, A:E208, A:Q209, A:P210, A:A211, A:P212, A:A213, A:P214, A:K215 | 39 | 0.67 |
|  | 5 | A:E57, A:K58, A:E59, A:E62 | 4 | 0.592 |
| **PD** | 1 | A:N87, A:G88, A:Q89, A:D91, A:T92, A:N93, A:Q94, A:T95, A:E96, A:K97, A:P98, A:N99, A:E100, A:E101, A:K102, A:G103, A:H104, A:H105, A:H106 | 19 | 0.717 |
|  | 2 | A:G27, A:T28, A:K29, A:D30, A:N31, A:N32, A:E63, A:H64, A:N66, A:E67, A:R68, A:P69, A:H70, A:S71, A:D72, A:N73, A:G74, A:F75, A:G76, A:N77, A:D80, A:R84 | 22 | 0.656 |
|  | 3 | A:M1, A:E2, A:K3, A:V4, A:T5, A:D6, A:S7, A:S8, A:R10, A:Q11, A:L41, A:L42, A:A43, A:L44, A:L45, A:K46, A:G47, A:G48, A:G49 | 19 | 0.636 |

### Supplementary Table S21. Details of the interactions between the selected peptides (30-mer) and the A and B chains of HLA-DRB1_01:01 molecule.

| **Complex** | **Peptide Name** | **Sequence** | **Lowest Interaction**  **Energy** | **No. of Salt Bridge** | **No. of Hydrogen Bonds** | **No. of Non-bonded**  **contacts** |
| --- | --- | --- | --- | --- | --- | --- |
| A | A Peptide | EAPVASQSKAEKDYDAAVKKSEAAKKHYEE | -674.6 | 3 | 17 | 213 |
| B |  | KKSEAAKKHYEEVKKKAEDAQKKYDEGQKK | -668.1 | 3 | 11 | 164 |
| C | Cons-Clade 1+2 | LKEIDESDSEDYIKEGFRVPLQSELDAKR | -644.4 | 0 | 7 | 109 |
| D | Cons-Clade 1 | KLEKDVEYFKNTDGEYTEQYLEAAEK | -835.6 | 1 | 9 | 146 |
| E | Cons-Clade 3+4 | ELEKLLDTLDPEGKTQDELDKEAAEAELDK | -391.5 | 1 | 11 | 132 |
| F | Cons-Clade 4+5 | LTRLEDNLKDAEENNVEDYIKEGLEKAI | -531.9 | 1 | 7 | 82 |
| G | C Peptide | PAPAPKPEQPAPAPK | -524.1 | 0 | 8 | 87 |
| H | PhtD-C Peptide | SLEDLLATVKYYVEHPNERPHSDNGFGNAS | -943.9 | 3 | 13 | 170 |
| I |  | HPNERPHSDNGFGNASDHVQRNKNGQADTN | -712.7 | 2 | 12 | 146 |
| J | PhtD-Cˊ Peptide | EKVTDSSIRQNAVETLTGLKSSLLLGTKDN | -815.6 | 4 | 14 | 179 |

### Supplementary Table S22. Gene sequences of *PAD*, *PA* and *PD*. The TAA-stop codon was used in the 3′ end*,* and *Nco*I and *Xho*I sites were inserted in 5′ and 3′ ends, respectively.

| **Gene name** | **Sequence from 5´ to 3´**  (Cloning Sites: 5´ *Nco*I / *Xho*I 3´) |
| --- | --- |
| ***PAD***  (998 bp) | CC**ATG**GAAGCTCCGGTTGCTTCTCAGTCTAAAGCTGAAAAAGACTACGACGCTGCTGTTAAAAAATCTGAAGCTGCTAAAAAACACTACGAAGAAGTTAAAAAAAAAGCTGAAGACGCTCAGAAAAAATACGACGAAGGTCAGAAAAAAACCGTTGAAAAAGCTAAACGTGAAAAAGAAGCTAGTGAAAAAATCGCTGGTGGTGGTTCTGAACTGGAAAAACTGCTGGACACCCTGGACCCGGAAGGTAAAACCCAGGACGAACTGGACAAAGAAGCTGCTGAAGCTGAACTGGACAAAAAAGTTGGTGGTGGTTCTCTGACCCGTCTGGAAGACAACCTGAAAGACGCTGAAGAAAACAACGTTGAAGACTACATCAAAGAAGGTCTGGAAAAAGCTATCGGTGGTGGTTCTCTGAAAGAAATCGACGAATCTGACTCTGAAGACTACATCAAAGAAGGTTTCCGTGTTCCGCTGCAGTCTGAACTGGACGCTAAACGTGGTGGTGGTTCTAAACTGGAAAAAGACGTTGAATACTTCAAAAACACCGACGGTGAATACACCGAACAGTACCTGGAAGCTGCTGAAAAAGGTGGTGGTTCTCCGGCTCCGGCTCCGAAACCGGAACAGCCGGCTCCGGCTCCGAAAGGTGGTTCTTCTGGTGGTGAAAAAGTTACCGACTCTTCTATCCGTCAGAACGCTGTTGAAACCCTGACCGGTCTGAAATCTTCTCTGCTGCTGGGTACCAAAGACAACAACACCATCTCTGCTGAAGTTGACTCTCTGCTGGCTCTGCTGAAAGGTGGTGGTTCTCTGGAAGACCTGCTGGCTACCGTTAAATACTACGTTGAACACCCGAACGAACGTCCGCACTCTGACAACGGTTTCGGTAACGCTTCTGACCACGTTCAGCGTAACAAAAACGGTCAGGCTGACACCAACCAGACCGAAAAACCGAACGAAGAAAAAGGTCACCACCACCACCACCAC**TAA**CTCGAG |
| ***PA***  (677 bp) | CC**ATG**GAAGCTCCGGTTGCTTCTCAGTCTAAAGCTGAAAAAGACTACGACGCTGCTGTTAAAAAATCTGAAGCTGCTAAAAAACACTACGAAGAAGTTAAAAAAAAAGCTGAAGACGCTCAGAAAAAATACGACGAAGGTCAGAAAAAAACCGTTGAAAAAGCTAAACGTGAAAAAGAAGCTAGTGAAAAAATCGCTGGTGGTGGTTCTGAACTGGAAAAACTGCTGGACACCCTGGACCCGGAAGGTAAAACCCAGGACGAACTGGACAAAGAAGCTGCTGAAGCTGAACTGGACAAAAAAGTTGGTGGTGGTTCTCTGACCCGTCTGGAAGACAACCTGAAAGACGCTGAAGAAAACAACGTTGAAGACTACATCAAAGAAGGTCTGGAAAAAGCTATCGGTGGTGGTTCTCTGAAAGAAATCGACGAATCTGACTCTGAAGACTACATCAAAGAAGGTTTCCGTGTTCCGCTGCAGTCTGAACTGGACGCTAAACGTGGTGGTGGTTCTAAACTGGAAAAAGACGTTGAATACTTCAAAAACACCGACGGTGAATACACCGAACAGTACCTGGAAGCTGCTGAAAAAGGTGGTGGTTCTCCGGCTCCGGCTCCGAAACCGGAACAGCCGGCTCCGGCTCCGAAAGGTCACCACCACCACCACCAC**TAA**CTCGAG |
| ***PD***  (338 bp) | CC**ATG**GAAAAAGTTACCGACTCTTCTATCCGTCAGAACGCTGTTGAAACCCTGACCGGTCTGAAATCTTCTCTGCTGCTGGGTACCAAAGACAACAACACCATCTCTGCTGAAGTTGACTCTCTGCTGGCTCTGCTGAAAGGTGGTGGTTCTCTGGAAGACCTGCTGGCTACCGTTAAATACTACGTTGAACACCCGAACGAACGTCCGCACTCTGACAACGGTTTCGGTAACGCTTCTGACCACGTTCAGCGTAACAAAAACGGTCAGGCTGACACCAACCAGACCGAAAAACCGAACGAAGAAAAAGGTCACCACCACCACCACCAC**TAA**CTCGG |

### Supplementary Table S23. Ratio of anti-PA or anti-PD IgG isotypes in different groups of mice.

| **Antibody** | **Groups** | **IgG1/IgG2a Ratio** |
| --- | --- | --- |
| Anti-PA IgG | PA  PA+PD  PAD | 0.95  0.94  0.95 |
| Anti-PD IgG | PD  PA+PD  PAD | 1.57  1.2  2.14 |

**1.2 Supplementary Figures**

### Supplementary Figure S1.

Supplementary Figure S1. Phylogenetic tree of A region sequences of PspA from 123 pneumococcal strains. The non-repetitive sequences of region A were aligned by MEGA software and the phylogenetic tree of aligned sequences was drawn using Neighbor-Joining method. The A sequence from the reference strain AC122 was chosen as a representative to obtain the epitopes of this region.


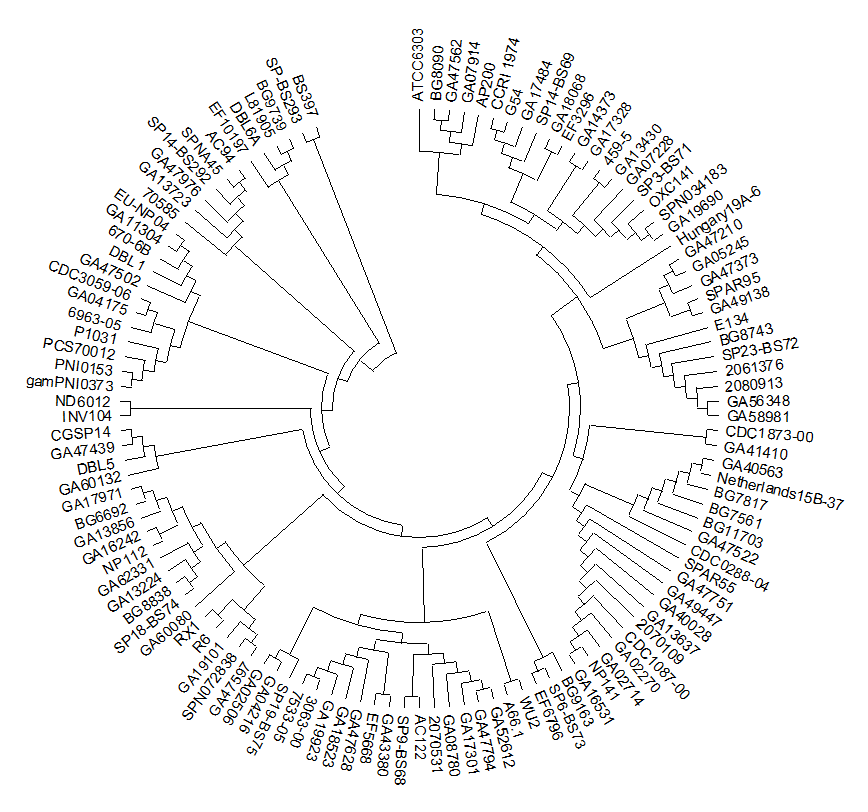


### Supplementary Figure S2.


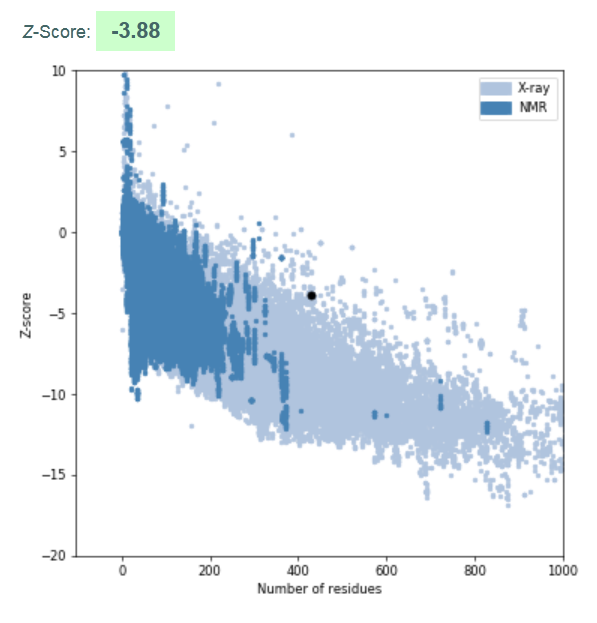

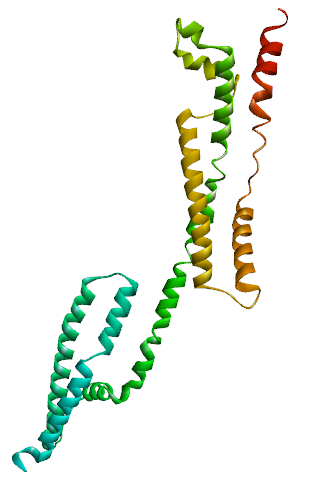


Supplementary Figure S2. 3D model of PspA strain AC122 and model validation. (A) The refined 3D structure was visualized by Discovery Studio Visualizer. (B) The Z-score of the structure was estimated to be -3.88, which is within the range of the score of natural protein structures.

**A B**

### Supplementary Figure S3.

Supplementary Figure S3. 3D models of the B regions of PspA1-5. The modeled structures of the B regions of PspA clades 1 to 5 (B1 to B5, respectively) were visualized by Discovery Studio Visualizer.


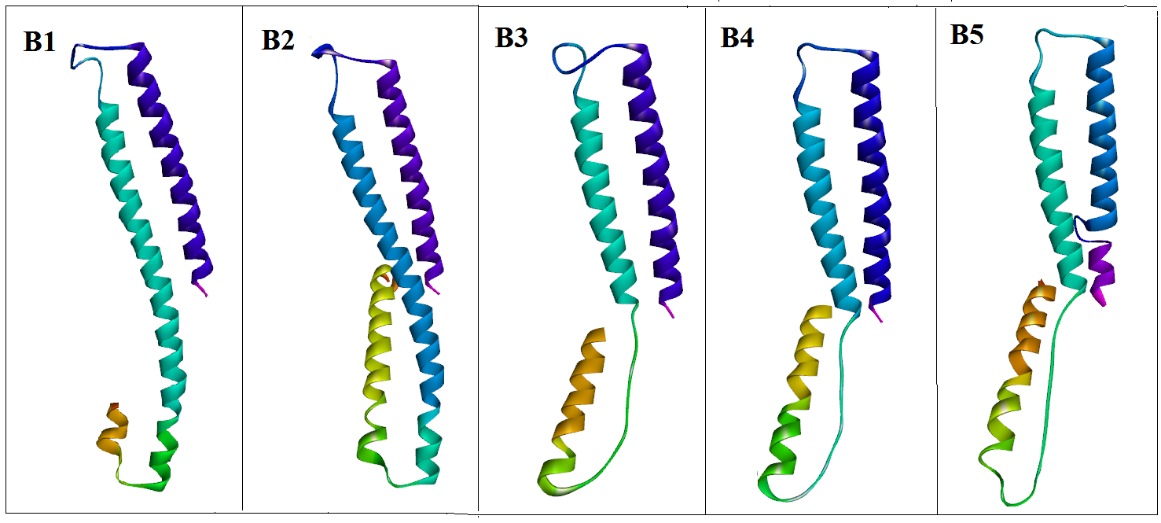


### Supplementary Figure S4.

**Supplementary Figure S4. 3D model of PhtD-C.** The predicted 3D structure was visualized by Discovery Studio Visualizer.


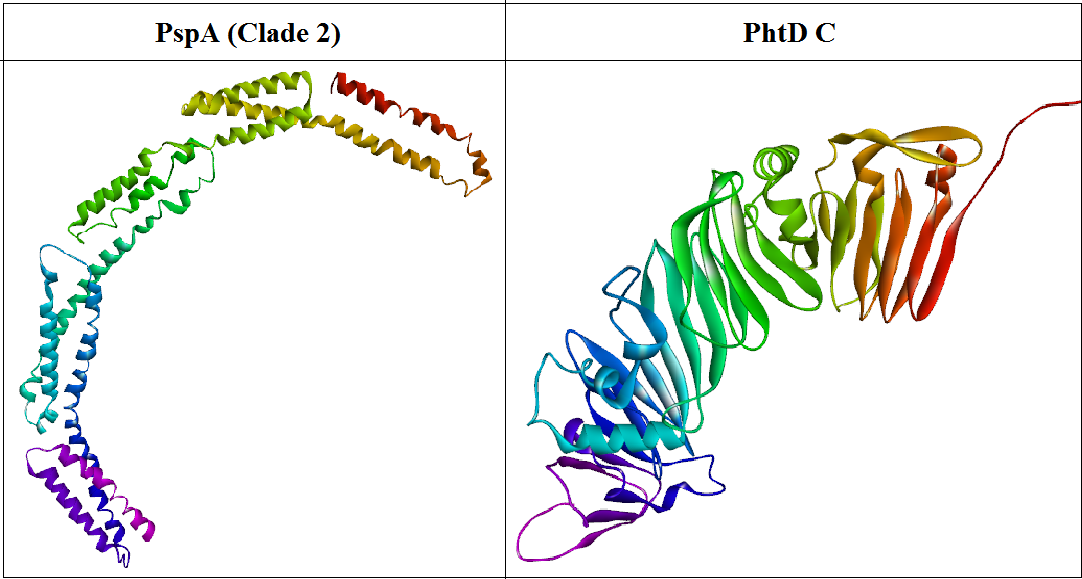


**PhtD**

### Supplementary Figure S5.


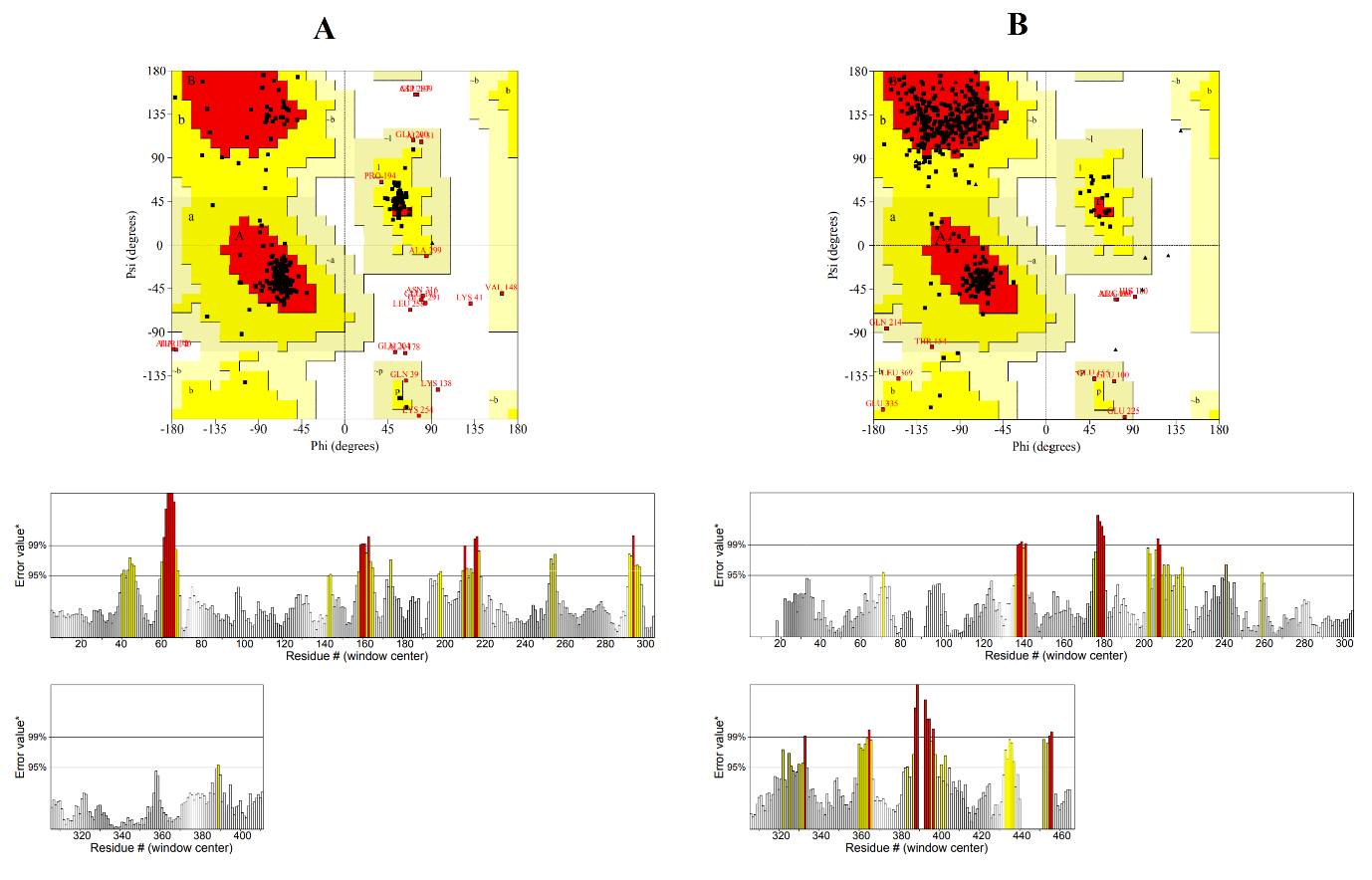


**Supplementary Figure S5. Validation of the refined PhtD-C model.** The Ramachandran plot shows that 87.2% of the residues are located in the favorable areas. In the ERRAT plot, the overall quality factor of the structure is 85.23%.

**A**

**B**

### Supplementary Figure S6.


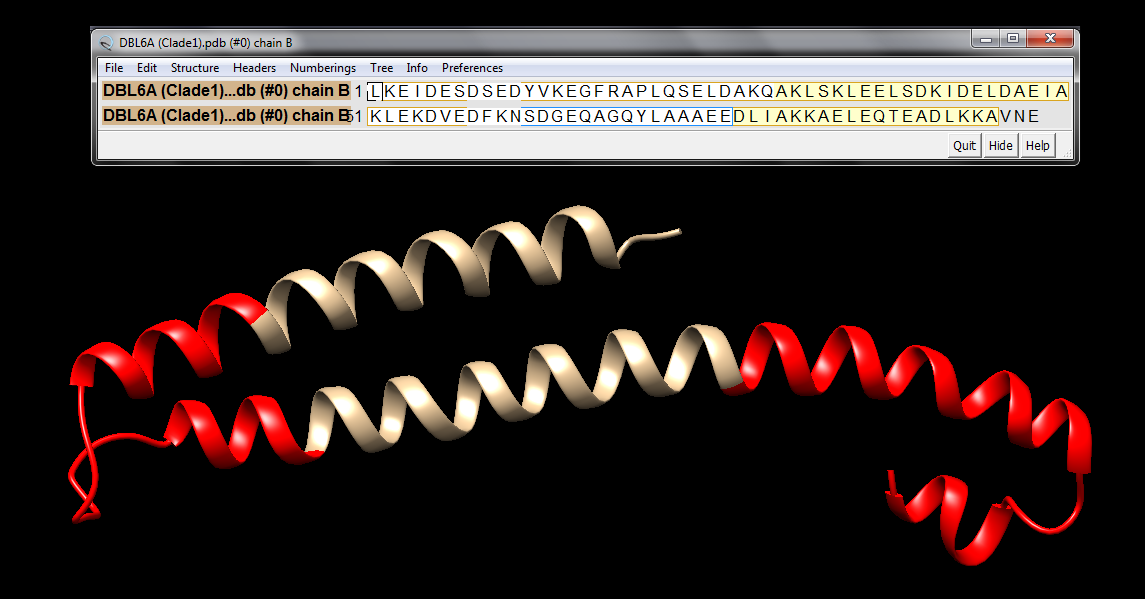


Supplementary Figure S6. Display of selected epitopes of B region from DBL6A strain (Clade 1) on 3D structure by Chimera software. the selected epitopes are shown in red on the structure.

### Supplementary Figure S7.


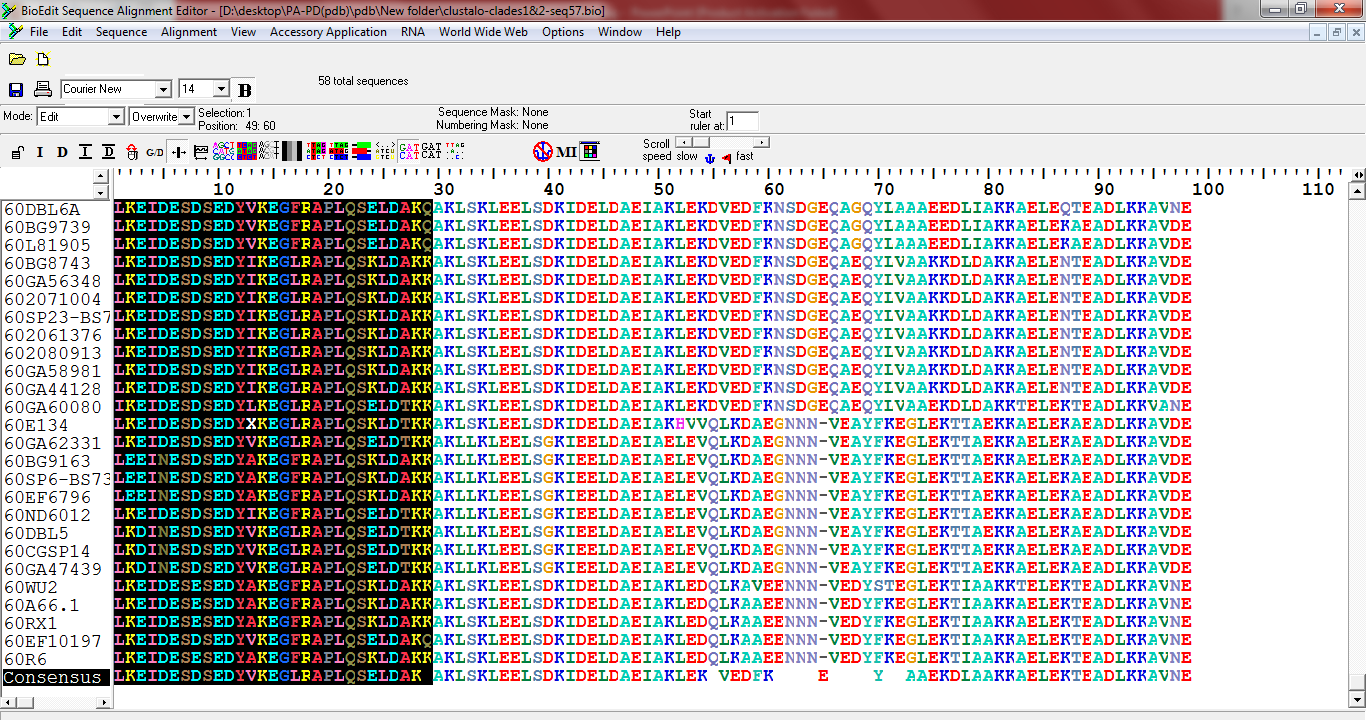

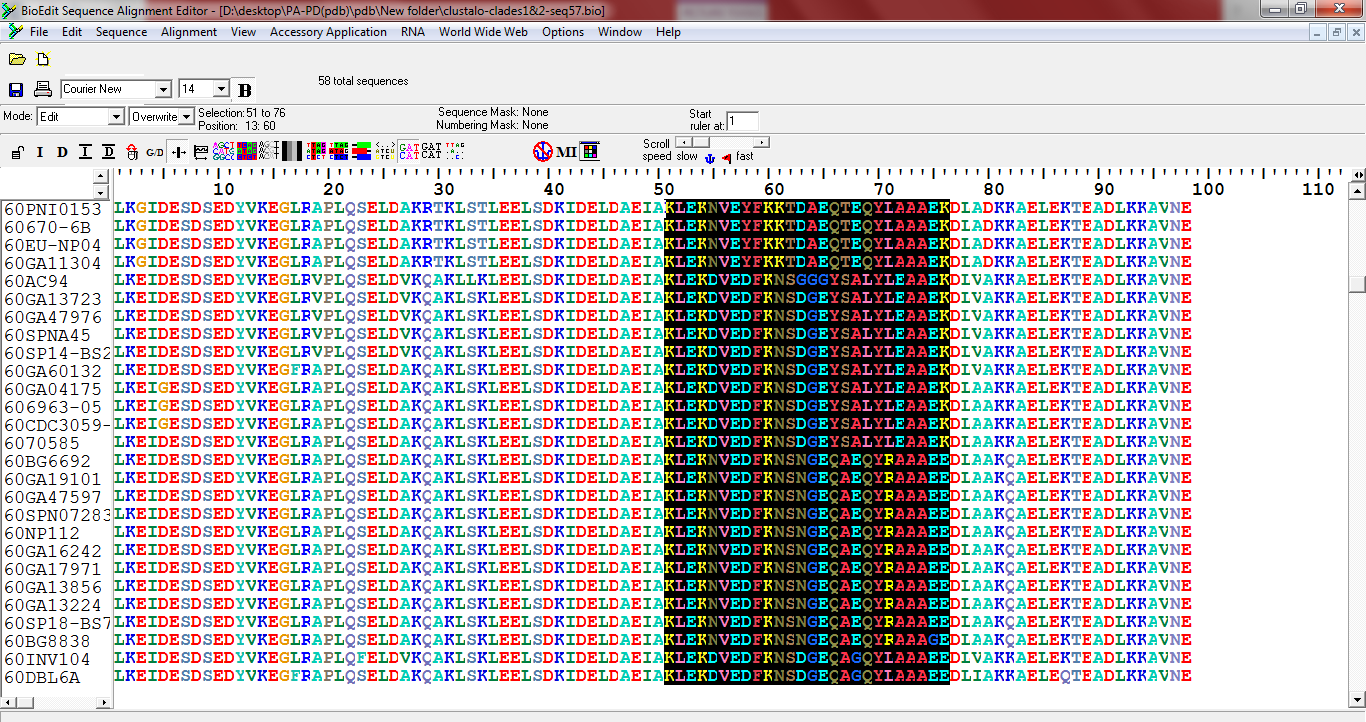


**A**

**B**

Supplementary Figure S7. Selection of consensus epitope sequence of region B for different strains using BioEdit software. (A) Selection of consensus sequence of B region for different strains of Clades 1 and 2. (B) Selection of consensus sequence of B region for different strains of Clade 1.

**LKEIDESDSEDYIKEGFRVPLQSELDAKR**

**KLEKDVEYFKNTDGEYTEQYLEAAEK**

**Consensus:**

**Consensus:**

### Supplementary Figure S8.


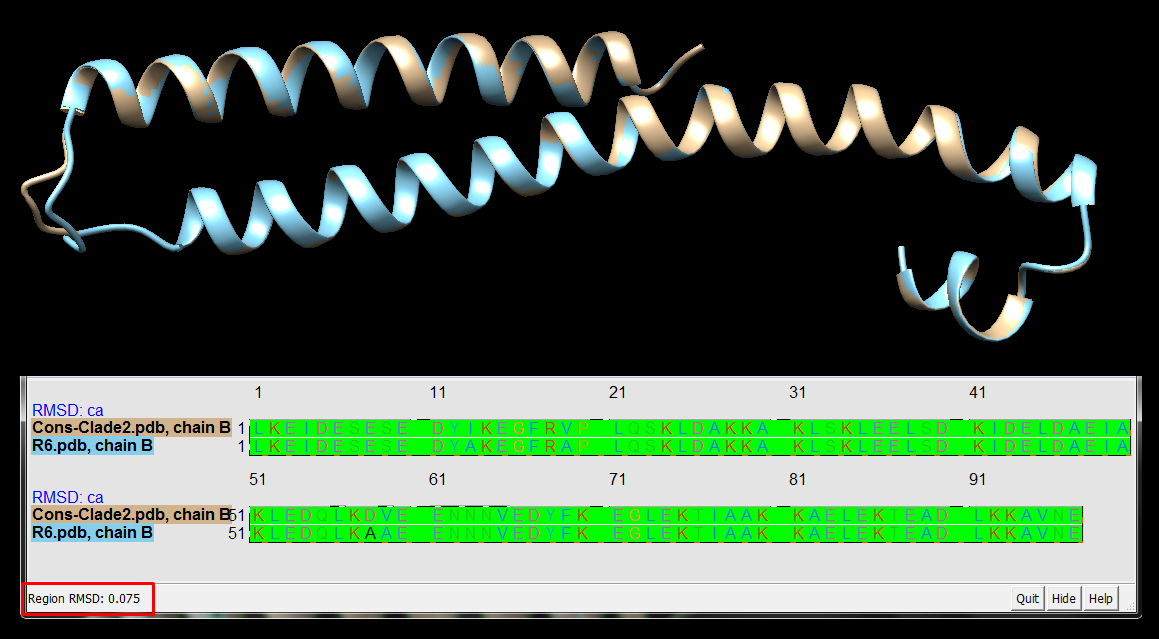


Supplementary Figure S8. Superimposition of two modeled structures of the primary protein (PspA2-B, blue color) and the peptide containing the consensus sequence (grey color). RMSD close to zero indicates that there is the overall structural similarity between the peptides and the structure and folding of the resulting antigen is reliable.

### Supplementary Figure S9.

**Supplementary Figure S9. Validation of the models before refinement.** (A) The Ramachandran plots show that in the initial models PAD, PA, and PD, 89.6%, 92.3,% and 84.9% of the residues are located in the favorable areas, respectively. (B) The ProSA Z-scores are found to be -5.38, -3.48, and -6.63 in the primary models PAD, PA, and PD, respectively. (C) In the ERRAT plots, the overall quality factors of the structures PAD, PA, and PD are 93.96, 95.14, and 89.24, respectively.


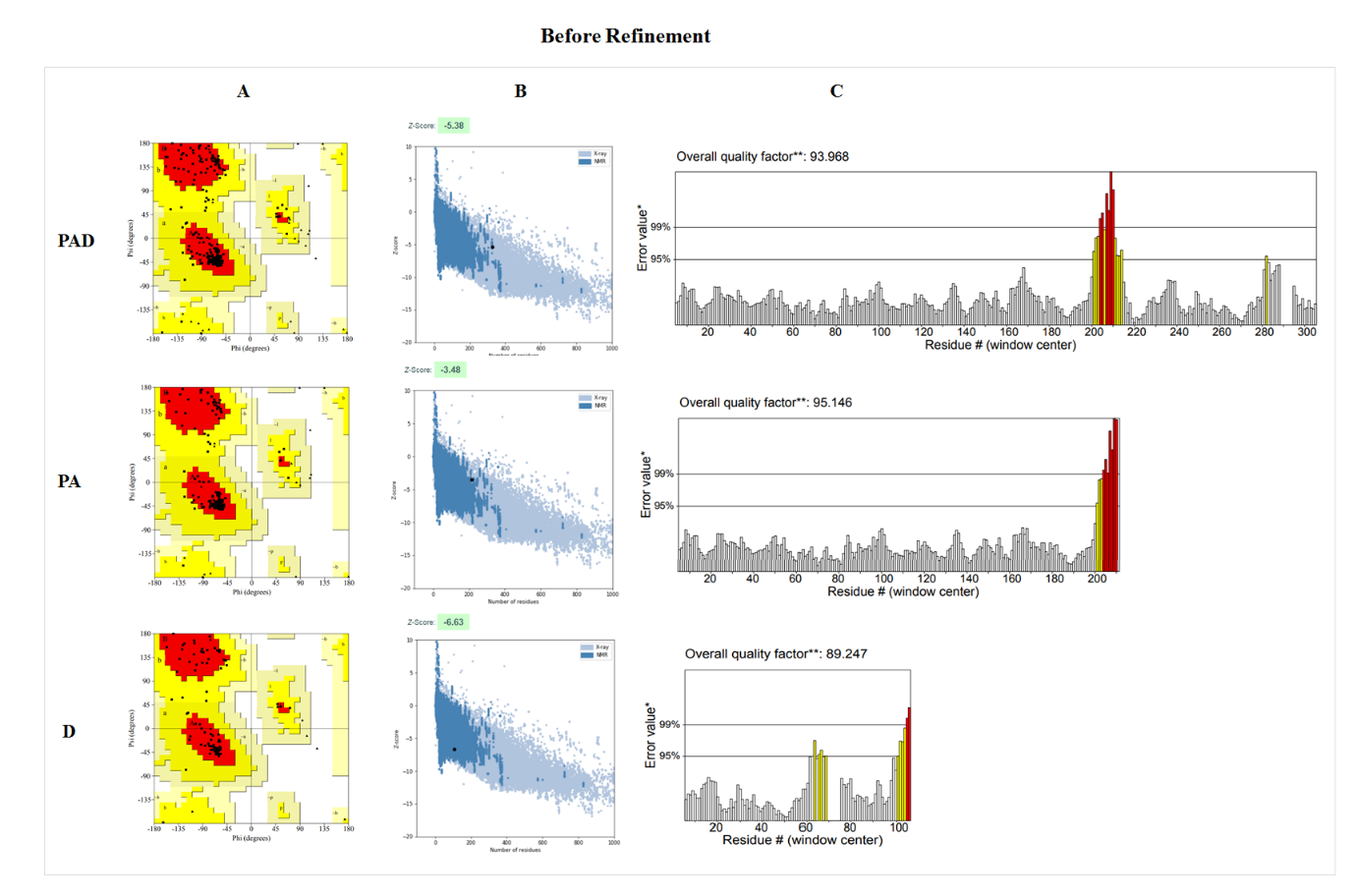


### Supplementary Figure S10.


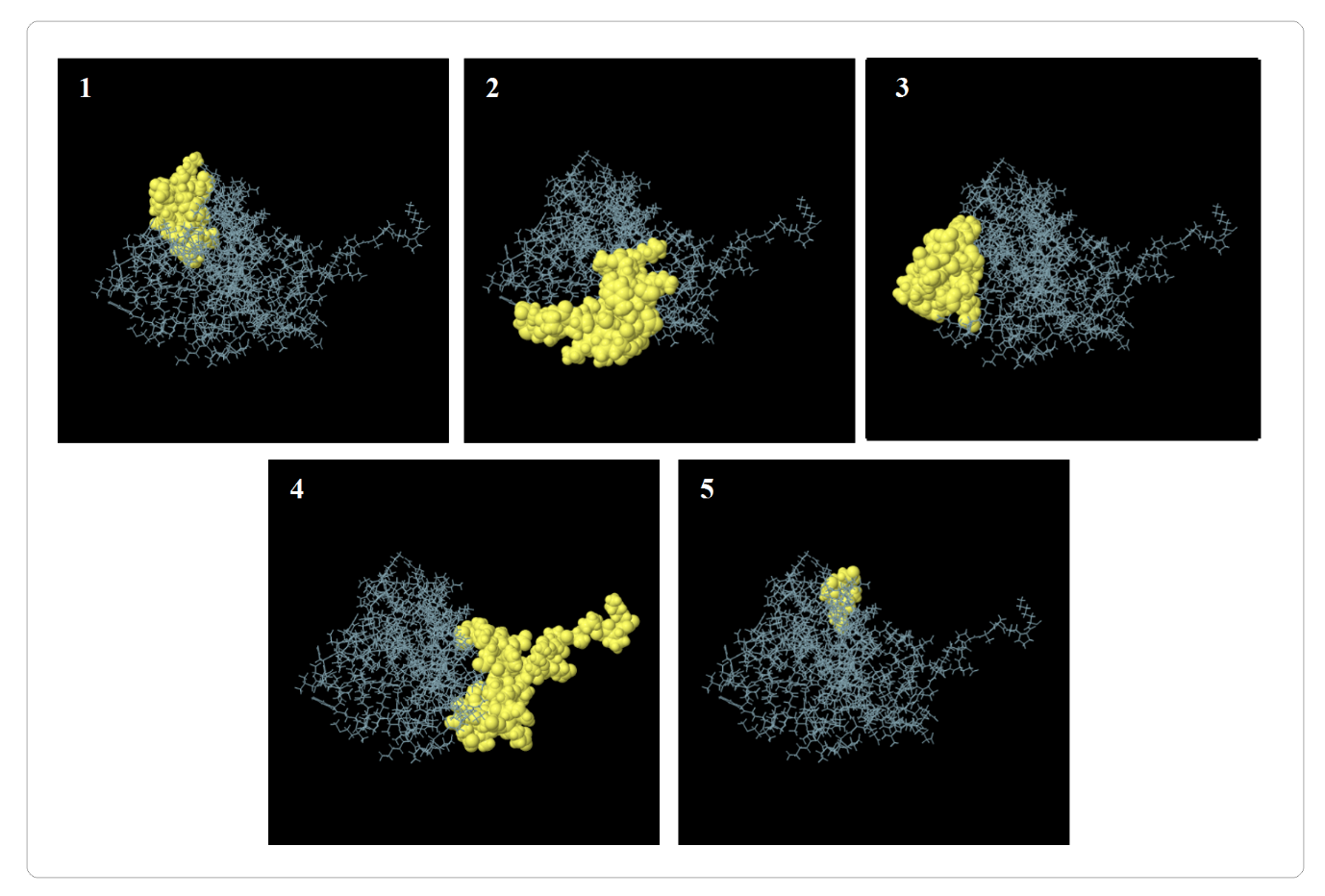


**Supplementary Figure S10. 3D images of structural B cell epitopes of the PA construct and 2D score chart.** The yellow and gray areas represent the structural epitopes and the other protein segments, respectively.

### Supplementary Figure S11.


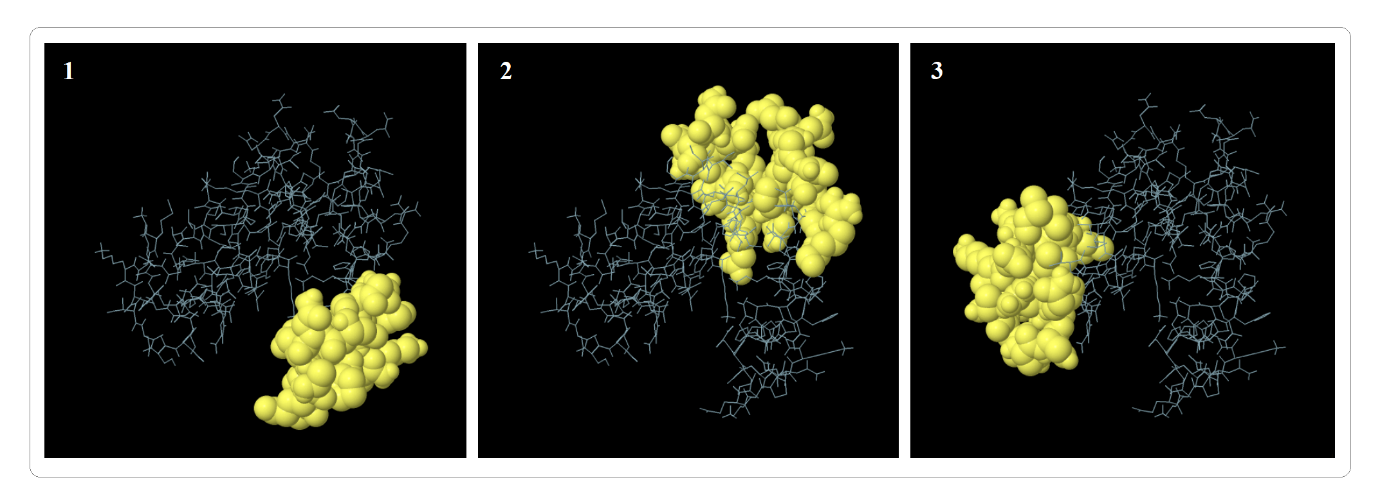


**Supplementary Figure S11. 3D images of structural B cell epitopes of the PD construct and 2D score chart.** The yellow and gray areas represent the structural epitopes and the other protein segments, respectively.

### Supplementary Figure S12.

**Supplementary Figure S12.** *In silico* immune simulation of the PA construct. (A) Immunoglobulin production in response to injection of the PA construct (antigens and immunoglobulin subclasses are represented as black and colored peaks, respectively). (B) Evolution of the B cell population after 4 injections. (C) Evolution of the T-helper cell population. (D) Levels of cytokines and interleukins after 4 injection steps.


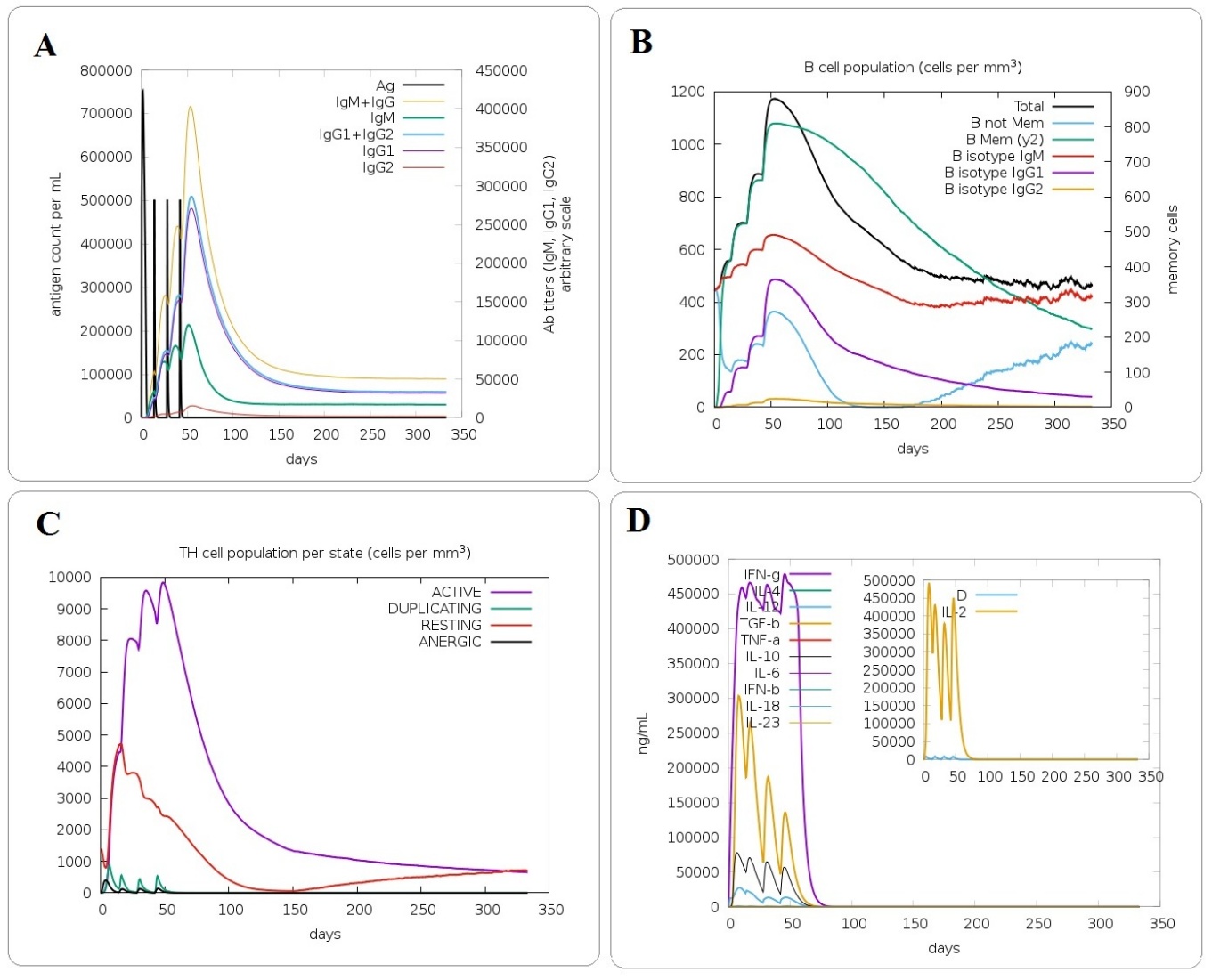


### Supplementary Figure S13.

**Supplementary Figure S13.** *In silico* immune simulation of the PD construct. (A) Immunoglobulin production in response to injection of the PD construct (antigens and immunoglobulin subclasses are represented as black and colored peaks, respectively). (B) Evolution of the B cell population after 4 injections. (C) Evolution of the T-helper cell population. (D) Levels of cytokines and interleukins after 4 injection steps.


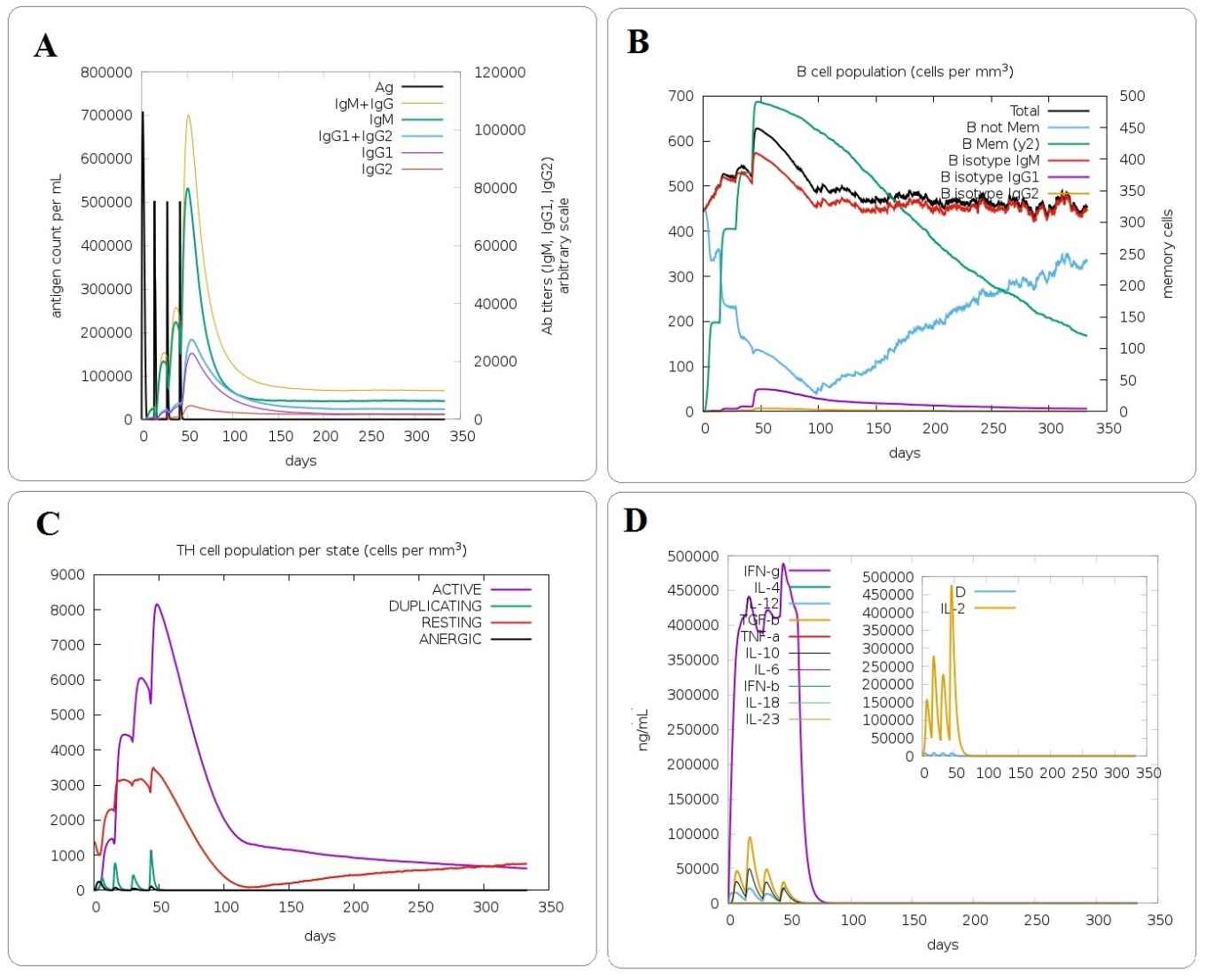


### Supplementary Figure S14.


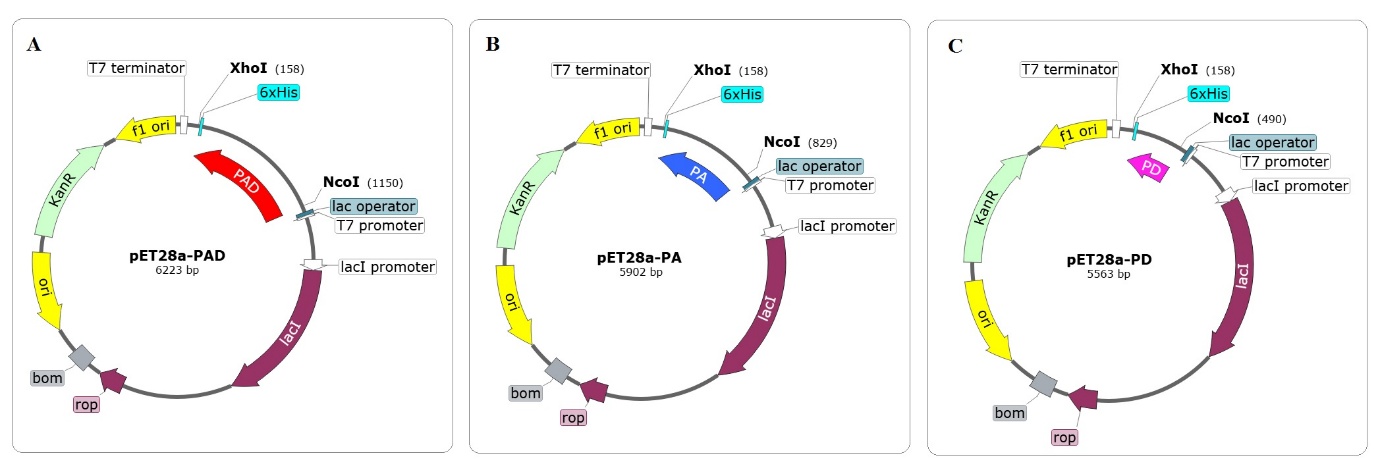


Supplementary Figure S14. *In silico* cloning of the gene sequences *PAD*, *PA* and *PD* in pET28a(+) plasmids. The developed plasmids were designated as pET28a-PAD (A), pET28a-PA (B), and pET28a-PD (C). The sequences of *PAD*, *PA* and *PD* between the restriction enzymes are shown in red, blue, and magenta, respectively. A His-tag sequence was added at the 3′ end of the insert, shown in cyan. The target gene is under the control of T7 promoter and terminator. The expression vector contains the gene for resistance to the antibiotic kanamycin.

### Supplementary Figure S15.


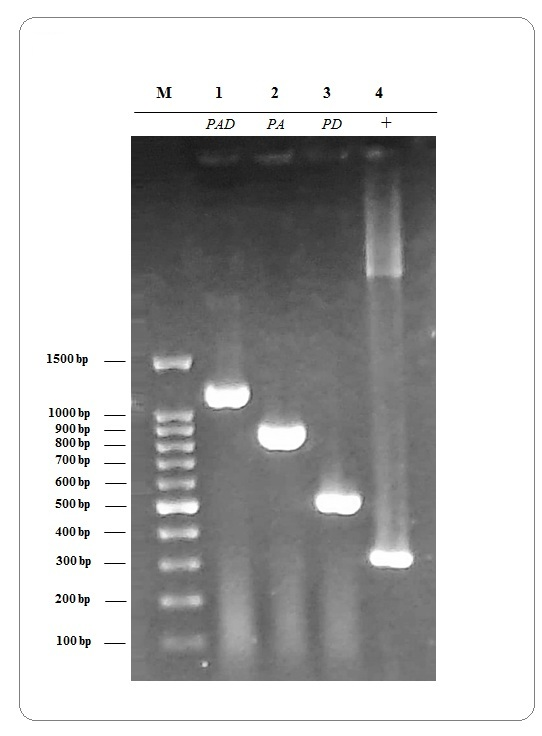


**Supplementary Figure S15. Verification of bacterial transformation.** Colony PCR on the positive clones using T7 universal primers. The sizes of the nucleotide sequences of the *PAD*, *PA* and *PD* genes are 1004, 683 and 344 bp, respectively. Since the amplifications were performed with the vector backbone-specific primers, the size of the amplified fragments were about 180 bp larger than the expected sizes (corresponding to the regions of the vector from the primer annealing sites to the restriction sites). Lane M: 1Kb DNA marker; Lanes 1 to 3: Amplified fragment of the recombinant pET28a vector including the sequences of *PAD*, *PA* or *PD*, respectively; Lane 4: A fragment of an empty plasmid pET28a without insert as control (318 bp).
